# CNRein: an evolution-aware deep reinforcement learning algorithm for single-cell DNA copy number calling

**DOI:** 10.1186/s13059-025-03553-2

**Published:** 2025-04-07

**Authors:** Stefan Ivanovic, Mohammed El-Kebir

**Affiliations:** 1 Department of Computer Science, University of Illinois Urbana-Champaign, Urbana, IL 61801 USA; 2 Cancer Center Illinois, University of Illinois Urbana-Champaign, Urbana, IL 61801 USA

## Abstract

Low-pass single-cell DNA sequencing technologies and algorithmic advancements have enabled haplotype-specific copy number calling on thousands of cells within tumors. However, measurement uncertainty may result in spurious CNAs inconsistent with realistic evolutionary constraints. We introduce evolution-aware copy number calling via deep reinforcement learning (CNRein). Our simulations demonstrate CNRein infers more accurate copy-number profiles and better recapitulates ground truth clonal structure than existing methods. On sequencing data of breast and ovarian cancer, CNRein produces more parsimonious solutions than existing methods while maintaining agreement with single-nucleotide variants. Additionally, CNRein shows consistency on a breast cancer patient sequenced with distinct low-pass technologies.

**Supplementary Information:**

The online version contains supplementary material available at 10.1186/s13059-025-03553-2.

## Background

Cancer results from an evolutionary process during which somatic mutations accumulate in a population of cells, resulting in intra-tumor heterogeneity, i.e., the presence of multiple cellular subpopulations, or *clones*, with distinct mutations [1]. Copy number aberrations (CNAs) are a very common form of mutation in cancer, on average affecting 44% of the genome in solid tumors [2–4]. Unlike single-nucleotide variants (SNVs), individual CNAs can simultaneously affect thousands of genes by amplifying or deleting large regions of the genome. Consequently, the identification of CNAs is vital for understanding cancer evolution. Additionally, identifying CNAs is of great clinical utility, since there exist certain drug therapies that target vulnerabilities in the cancer that arise as a result of specific CNAs [5–7]. However, the combination of intra-tumor heterogeneity and limitations of current sequencing technologies render copy number calling a challenging task.

Several works have focused on copy number calling from bulk DNA sequencing data [8–14]. Two key signals are used for copy number calling in these data. First, the number of reads, or *read depth*, of a genomic region is proportional to its total number of copies. Second, the *B-allele frequency* (BAF) of heterozygous germline single nucleotide polymorphisms (SNPs) is indicative of allelic imbalance, where a BAF of 0 (1) indicates the absence of B (A) alleles and a BAF of 0.5 indicates the same number of A and B alleles. Rather than providing measurement of individual cells, bulk DNA sequencing provides composite measurements of read depth and BAF of millions of cells in the bulk sample. This composite signal obscures intra-tumor heterogeneity and requires deconvolution, resulting in an inevitable loss of signal. Unlike bulk sequencing, single-cell sequencing technologies potentially enable the precise characterization of copy number in individual cells. Due to the popularity of single-cell RNA sequencing, a wide variety of algorithms exist for determining CNAs on these data such as Numbat [15], CopyKAT [16], HoneyBadger [17], and InferCNV [18]. However, as read depth in these data is additionally confounded by expression, it is extremely difficult to quantitatively determine integer copy numbers, and, instead, only the qualitative presence of gains, deletions and loss of heterozygosity (LOH) is predicted.

By contrast, single-cell DNA sequencing technologies have enabled the sequencing of hundreds of cells at relatively uniform coverage, suitable for copy number calling [19]. Algorithms such as HMMcopy [20], Ginkgo [21], SCICoNE [22], SCNA [23], SCOPE [24], and VICTree [25] have been utilized to determine total copy numbers of regions on the genome on this single-cell data. More recent technologies, including 10x Genomics CNV solution [26], DLP+ [27, 28], and ACT [29], have improved upon first-generation technologies to enable high-throughput single-cell DNA sequencing of thousands of cells with lower error rates. Three recent methods, CHISEL [30] SIGNALS [31], and Alleloscope [32], have enabled the determination of *haplotype-specific copy numbers* on this newer type of single-cell data, indicating the number of copies of both parental haplotypes phased within each chromosome. CHISEL works by globally clustering bins across cells by read depth and BAF, and then jointly inferring the copy number of these clustered bins. SIGNALS first applies HMMcopy to obtain total copy number estimates and then utilizes the BAF measurement to determine the haplotype-specific state. Alleloscope first performs segmentation on bulk or pseudobulk data and then performs phasing and copy number calling. These algorithms have detected additional patterns on single-cell sequencing data not observable from total copy numbers such. Such patterns include copy-neutral loss of heterozygosity as well as mirrored-subclonal CNAs in which two subsets of cells contain the same number of gains or losses in the same genomic regions but on different alleles. However, these algorithms do not take advantage of biological constraints imposed by the evolutionary nature of cancer to jointly estimate copy number profiles across cells. Our key premise is that one should not infer CNAs in the absence of clear evidence from data and rather opt for shared clonal structure. In particular, one should only infer cell-specific CNAs when there is strong evidence in the read depth or BAF data. The temporal structure of evolutionary constraints addresses this by acting as a regularizer, reducing the number of spurious CNAs predicted due to noise in the data, as discussed in [22, 25, 33, 34].

**Fig. 1:**
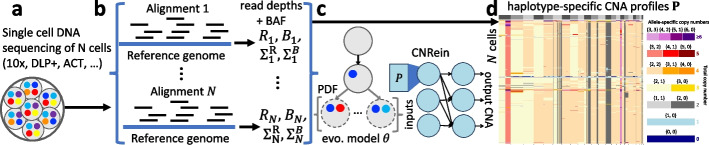
CNRein uses evolutionarily aware deep reinforcement learning to perform single-cell copy number calling. **a** Technologies such as 10x Genomics CNV solution [26], DLP+ [27, 28], and ACT [29] enable high-throughput single-cell DNA sequencing suitable for CNA calling. **b** Reads are aligned and processed to obtain CNRein inputs. **c** CNRein uses a generative model of CNA evolution parameterized by a convolutional neural network and optimized via reinforcement learning. **d** From the evolutionary model $\theta$, CNRein produces haplotype-specific copy number profiles **P** for each cell

We introduce CNRein, an evolution-aware deep reinforcement learning algorithm for haplotype-specific copy number calling. CNRein constrains predicted copy number profiles to result from a sequence of amplifications and deletions of copies of specific alleles in contiguous regions within chromosomes (Fig. 1). These sequences of CNA events form trajectories, starting from the normal cell, with a neural network constraining the likelihood of each CNA occurring. We use a deep reinforcement learning procedure to train the neural net to produce copy number profiles that maximize the probability of the observed read depth and BAF data. The predicted copy number profile for each cell balances fitting that cell’s read depth and BAF values with forming coherent evolutionary trajectories across all cells. Thus, the level of intra-tumor heterogeneity predicted by CNRein results from training on the data rather than the imposition of clonal structure via *post hoc* clustering of inferred copy-number profiles. This process is unsupervised in that it does not require any labeled data such as cells with known copy number profiles. Our deep reinforcement learning procedure has similarities with GFlowNets [35] in that both procedures learn a distribution over trajectories to overall fit the data rather than simply trying to maximize the expected reward as is done most typically in reinforcement learning. However, GFlowNets do not enable the optimization of our objective, namely maximizing the product of all cells’ read count probabilities rather than setting profile probabilities proportional to fixed, pre-specified rewards (Additional file 1: Section A.3.8). Additionally, CNRein does not rely on the presence of normal cells, enabling the analysis of data where only tumor cells have been sequenced through FACS sorting.

We compare CNRein with SIGNALS and CHISEL on simulated data generated using our own simulator as well as CNAsim [36]. We find that existing methods have a tendency to identify spurious CNAs whereas CNRein’s predictions better match ground truth due to its evolutionary constraints. On breast and ovarian cancers [27, 29], CNRein produces larger clones, fewer unique copy number profiles, and ultimately enables the inference of more parsimonious phylogenies than competing methods, while maintaining similar fit to read depth and agreement with orthogonal somatic single-nucleotide variant (SNV) data. Additionally, running CNRein on a breast cancer patient [29] sequenced with both 10x and ACT technologies demonstrates the consistency of CNRein’s predictions independent of sequencing technology. With the increasing availability of low-pass, high-throughput single-cell DNA sequencing data of tumors, we expect that CNRein’s more accurate copy number analyses will enable more precise analyses of intra-tumor heterogeneity.

## Results

### CNRein algorithm overview

The objective of CNRein is to estimate the parameters of an evolutionary model of CNAs that maximize the data probability of observed read counts. Then, given the model, we predict a copy number profile for each individual cell with maximum joint probability according to said evolutionary model and observed read counts.

In contrast to existing allele-specific methods, this joint estimation is evolution aware. Specifically, our algorithm generates paths to CNA profiles using an evolutionary model for copy number profiles that sequentially adds CNAs to clones starting from the normal clone. The probability of each CNA event in this trajectory is determined by a deep convolutional neural network. The reinforcement learning system implements the addition of CNA events to clones as actions and utilizes a reward that maximizes the probability of the observed read counts given the model. Intuitively, high rewards are given to copy number profiles that fit cells’ read depth and BAF data, especially when existing high probability copy number profiles do not yet fit those cells well. After training, inference is performed by selecting copy number profiles with the highest overall probability for each cell given both the observed read counts and evolutionary model probabilities. More details about the problem statement are given in the “Problem statement” section.

CNRein’s pipeline follows three stages: (i) data processing, (ii) initial copy number estimation, and (iii) final copy number estimation via deep reinforcement learning (Additional file 1: Fig. S1). First, in the data processing stage, the input is a BAM file with *N* read groups indicating cell barcodes and the outputs are read depths $\tilde{\textbf{R}}$, haplotype-specific read counts **A**, and variance estimates $\varvec{\Sigma }^R$ and $\varvec{\Sigma }^B$ for the read depth and B-allele frequency, respectively, of *L* segments in the genome (Additional file 1: Section A.1). To do so, we perform phasing and initially split the genome into *K* bins of length 100kb in order to perform GC bias and mappability correction and segmentation. Our phasing step utilizes bcftools [37] for detecting SNPs and SHAPE-IT 4 [38] together with the 1000 genomes reference panel [39] for phasing haplotype blocks. Second, our CNNaive algorithm performs initial copy number estimation separately for each cell, yielding approximate copy number numbers profiles $\tilde{\textbf{P}}$ (Additional file 1: Section A.2). To accomplish this for each cell, CNNaive uses low-noise regions to estimate ploidy or the cell-specific scaling factor and then determines the copy number profile that maximizes the probability of that cell’s read counts. Third, our evolution-aware deep reinforcement learning system inputs $\tilde{\textbf{R}}, \textbf{A}$, $\varvec{\Sigma }^R$, $\varvec{\Sigma }^B$, and $\tilde{\textbf{P}}$ in order to jointly estimate copy number profiles **P** for all cells in order to maximize the probability of the observed read count data (“CNRein algorithm details” section).

### Evaluation on simulated scDNA-seq data

We generated 20 simulation instances with $n=1000$ cells and $K=\text {27,283}$ bins of 100kb length that had varying levels of intra-tumor heterogeneity and half of which included a truncal whole-genome duplication (WGD). The noise levels were based on breast cancer patient S0 (Additional file 1: Fig. S2 and the “Evaluation on a breast cancer dataset sequenced with 10x Chromium CNV technology” section). Our simulator generates copy number profiles **P** in addition to read depths $\tilde{\textbf{R}}$ and haplotype-specific count measurements **A** by first generating an evolutionary tree starting with either a normal clone or a whole genome duplicated clone. Then, the simulator sequentially generates CNAs on existing clones, with each CNA potentially increasing the corresponding clone’s fitness and thus the number of cells in the sample that originate from that clone or its descendants. The probability of fitness increases controls the relative growth rates of clones and thus the overall level of intra-tumor heterogeneity including the number of cell-specific CNAs. This varying heterogeneity helps us evaluate if methods underestimate heterogeneity by missing legitimate CNAs or overestimate heterogeneity by inferring spurious CNAs. As our simulations generate intermediate data rather than BAM or FASTQ files, we were not able to include Alleloscope in our benchmarking and only compared CNRein to SIGNALS and CHISEL. Further details are provided in Additional file 1: Section A.4.3, and runtimes are provided in Additional file 1: Fig. S3.

**Fig. 2:**
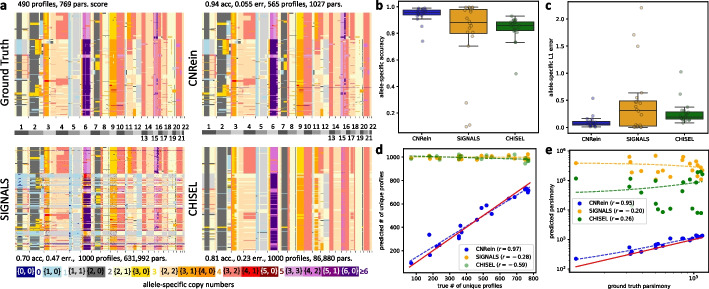
Results on simulated data. **a** Allele-specific copy number profiles from the ground truth as well as predictions by CNRein, SIGNALS, and CHISEL for a single simulation instance with a whole genome duplication at the trunk. **b** Accuracy values of unordered allele-specific copy numbers. **c** L1 errors of unordered allele-specific copy numbers. **d** The number of predicted unique copy number profiles (*y*-axis) vs. ground truth number of unique profiles (*x*-axis), with the $y=x$ line in red and Pearson correlations indicated in the key and dashed lines. A small amount of jitter (drawn from $\textrm{Unif}(-25,25)$) was added to predictions (*y*-axis) with exactly 1000 unique copy number profiles for clarity. **e** Parsimony scores for phylogenetic trees inferred from ground truth (*x*-axis) and predicted copy-number profiles (*y*-axis), with the ground truth $y=x$ line shown in red, and best-fit lines shown with dashes as well as Pearson correlations indicated in the key. The best-fit lines can appear slightly curved as a consequence of the plot’s log scale

Visually inspecting the copy number profiles of a single simulation instance with WGD (Fig. 2a), we find that CNRein’s predictions better match the ground truth compared to SIGNALS and CHISEL. To better quantify performance, we developed four different metrics. First, we defined *accuracy* as the average proportion of bins across all cells for which the ground truth allele-specific copy numbers were inferred (ignoring the ordering of alleles; see the “Evaluation details” section). For the simulation instances in Fig. 2a, CNRein’s accuracy was 0.948 compared to 0.704 for SIGNALS, and 0.816 for CHISEL (Fig. 2a). We see similar trends across all simulation instances, with CNRein achieving a median accuracy of 0.956, followed by 0.882 for SIGNALS, and 0.857 for CHISEL (Fig. 2b). Second, to better assess the magnitude of errors, we defined *error* as the mean absolute difference between predicted and ground truth copy numbers across all bins and cells (again allele-specific as mathematically defined in the “Evaluation details” section). For the simulation instance in Fig. 2a, CNRein achieved the smallest error of 0.055 and a median error of 0.064 across all simulation instances (Fig. 2c), followed by CHISEL (0.233 and 0.191, respectively) and then SIGNALS (0.470 and 0.315, respectively). In Additional file 1: Section B.3 and Fig. S4, we additionally analyzed sensitivity to focal CNAs (including CNAs smaller than 5 Mb) and error on haplotype-specific copy numbers, finding CNRein to have lower errors than SIGNALS or CHISEL in each circumstance.

Third, we compared the number of *unique copy number profiles* inferred by each method to that in the ground truth. The 1000 cells of the simulation instance shown in Fig. 2a correspond to 490 unique copy number profiles in the ground truth. While CNRein slightly overfit the data with 565 inferred unique copy number profiles, both SIGNALS and CHISEL inferred a unique copy number profile for each cell (Fig. 2a). Looking across all simulation instances (Fig. 2d), we find that CNRein better matched the true number of unique profiles with a Pearson correlation of $r=0.98$ and a median percentage error of 6.92% compared to SIGNALS ($r=-0.28$ and 110%) and CHISEL ($r=-0.59$ and 110%). We further confirm CNRein’s accurate reconstruction of intra-tumor heterogeneity by analyzing the number of cell-specific copy number profiles (Additional file 1: Fig. S5) and analyzing sets cells belonging to small clones (Additional file 1: Section B.4 and Fig. S6). We found CNRein’s predictions very closely matched the ground truth, in contrast to SIGNALS and CHISEL, which typically predicted cell-specific profiles independent of the ground truth.

Fourth, we utilized the copy number profiles inferred by each method to construct a phylogenetic tree as described in the “Evaluation details” section, defining the *parsimony score* as the number of CNA events on this tree using the zero-agnostic copy number transformation (ZCNT) distance inferred by Lazac [40]. For the simulation instance in Fig. 2a, CNRein predicted a far more parsimonious solution (with parsimony score 1346) than SIGNALS (769,219) or CHISEL (131,696), despite being less parsimonious than the ground truth (798). Note that high parsimony values may be reflective of homoplasy, i.e., identical CNAs occurring independently at many locations on the tree, due to a lack of evolutionary structure in the predictions. This can lead to high parsimony values even if the predictions do not look very visually noisy as is the case for CHISEL’s prediction. We find similar trends across all 20 simulation instances (Fig. 2e), with CNRein’s parsimony scores both lower and better correlated with the ground truth values (Pearson correlation $r=0.95$ and median value 1012) compared to SIGNALS ($r=-0.20$ and median value 248,891) and CHISEL ($r=0.26$ and median value 52,370). However, the median ground truth parsimony of 736 is even lower than CNRein’s parsimony, showing there still exists room for improvement.

In summary, CNRein infers more accurate copy-number profiles that better recapitulate ground truth clonal structure compared to SIGNALS and CHISEL. These findings are not unexpected, as unlike CNRein, neither SIGNALS nor CHISEL uses an evolutionary model to constrain inferred copy number profiles. In addition, we also compared against CHISEL’s *post hoc* clustered outputs and the state-of-the-art total copy number calling method VICTree [25], which directly infers clones. We find that CNRein outperforms these methods (Additional file 1: Section B.1 and Fig. S7). Finally, we performed additional benchmarking using an alternative simulator, CNAsim [36], finding that our method continues to outperform other methods (Additional file 1: Section B.2 and Fig. S8).

### Evaluation on an ovarian cancer dataset sequenced with DLP+ technology

Next, we benchmarked CNRein on 890 direct library preparation (DLP+) sequenced cells from three clonally-related cancer cell lines sourced from the same high-grade serous ovarian cancer OV2295 [27], with coverages ranging from $0.1\times$ to $0.4\times$ (Fig. S9). We compared CNRein to SIGNALS, CHISEL, and Alleloscope. As Alleloscope and SIGNALS applied additional filtering criteria, we restricted our analysis to the $n=617$ cells for which all methods were run and produced predictions.

**Fig. 3:**
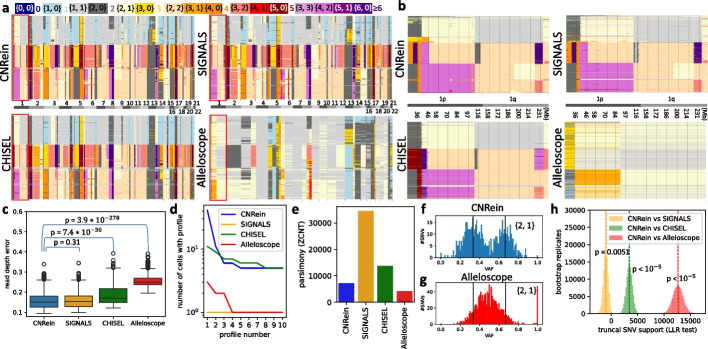
Results on high-grade serous ovarian cancer OV2295 [27]. **a** Allele-specific copy number profiles for CNRein, SIGNALS, CHISEL, and Alleloscope on $n=617$ cells. Unlike other methods, Alleloscope did not predict WGD for any cell (as indicated by ploidies above 3.0). **b** Allele-specific copy number profiles of chromosome 1, showing that while CNRein retains the ability to detect small CNAs also detected by SIGNALS. **c** SIGNALS and CNRein have a substantially closer fit to the raw read depth data than alternative methods. **d** The size of the 10 largest clones for each method, showing larger clones for CNRein and CHISEL than SIGNALS and Alleloscope. **e** CNRein’s predictions resulted in a more parsimonious phylogeny than SIGNALS or CHISEL, while Alleloscope resulted in the lowest parsimony score. **f**, **g** VAF values on the copy number 1, 2 for CNRein and Alleloscope. For CNRein but not Alleloscope, VAFs are concentrated around 1/3 and 2/3 (indicated with black vertical lines) as expected. **h** Log likelihood ratios (LLR) of truncal SNV support on bootstrapped replicates between our method and the alternative methods SIGNALS, CHISEL, and Alleloscope. CNRein outperforms CHISEL (LLR 3,418.66 and $p < 10^{-5}$) and especially Alleloscope (LLR 12,635.36 and $p < 10^{-5}$) while being slightly outperformed by SIGNALS (LLR $-1{,}033.42$ and $p = 0.0051$)

We find that the difference in predicted copy number profiles between Alleloscope and all other methods is very stark (L1 distances at least 1.35 compared to a distance of 0.14 between CNRein and SIGNALS shown in Additional file 1: Fig. S10), as a result of Alleloscope predicting no WGD while all other methods predicted WGDs on many cells (Fig. 3a). For the other methods, the differences are more subtle with the zoomed in plots of chromosome 1 showing (i) a lower level of variance in CNRein outputs, (ii) that CNRein’s and SIGNAL’s predictions are most similar, (iii) while CHISEL’s predictions have more substantial differences, especially towards telomeres of chromosomes (Fig. 3b). Looking at all $n=617$ cells and the complete genome, we assessed the fit of the predicted total copy number of each method to the input read depth (defined in Additional file 1: Section B.7). We found that CNRein has a statistically significant closer fit to the read depth data than CHISEL or Alleloscope, while having no statistically significant difference in fit relative to SIGNALS (Fig. 3c). Despite fitting the read depth data at least as well as the other methods, we find that CNRein predicted more shared clonal structure. That is, SIGNALS predicted a unique copy number profile for each cell, while the largest clones for CNRein, CHISEL, and Alleloscope contained 41, 9, and 2 cells, respectively (Fig. 3d). In total, CNRein, CHISEL, and Alleloscope predicted 468, 469, and 610 unique profiles, respectively.

To assess the downstream effects of overfitting and potentially inferring spurious CNAs, we constructed phylogenies from each method’s copy number profiles and evaluated the resulting parsimony scores via a similar procedure described in the “Evaluation on simulated scDNA-seq data” section. We find that CNRein’s tree had a parsimony score of 7163 (Fig. 3e), which is far more parsimonious than the trees produced from SIGNALS’ and CHISEL’s copy number profiles, achieving parsimony scores of 34,529 and 13,769, respectively. This difference in parsimony is visually clear in images of the full trees shown in Additional file 1: Fig. S11, showing long branches near the leaves indicative of many CNAs occurring on individual cells rather than on larger clones. We note that Alleloscope achieved an even lower parsimony score of 4149; however, we believe this is due to Alleloscope incorrectly predicting a lack of WGD.

To test the above hypothesis and orthogonally validate our CNRein’s predictions, we evaluated consistency with orthogonal SNV (single nucleotide variant) data reported by Laks et al. [27]. By SNVs we refer to somatic mutations, which, in contrast to germline SNPs, are not used by any method for inference. Specifically, we focused on a subset of 2,801 out of 14,068 SNVs that are likely *truncal* (as indicated by occurring in all three samples with precise details provided in the “Evaluation details” section). Truncal SNVs that occur on a segment with copy number $\{X^A, X^B\}$ must have a variant allele frequency (VAF) on those cells equal to some integer multiple of $1 / (X^A + X^B)$. We find that this is the case for allele-specific copy number $\{2,1\}$ for CNRein (Fig. 3f) as well as SIGNALS and CHISEL (Additional file 1: Fig. S12), showing bimodal distributions with peaks around 1/3 and 2/3, but not Alleloscope, showing a unimodal distribution with a single peak around 1/2 (Fig. 3g). Moreover, allelic mirroring for the CNA {2, 1} (demonstrated in Additional file 1: Fig. S13) is predicted by all methods and reflected in the VAFs of truncal SNVs for CNRein, SIGNALS, CHISEL but not Alleloscope, finding that SNVs with a VAF near 1/3 for (2, 1) have a VAF near 2/3 for (1, 2) and vice versa (Additional file 1: Fig. S14). We see similar trends for other CNAs (Fig. S15, Fig. S16, and Fig. S17), finding several cases where SNVs show evidence for LOH whereas Alleloscope inferred heterozygous CNAs.

To better quantify differences between methods, we developed a statistical test for goodness of fit with the SNV data, exploiting the fact that the vast majority of truncal SNVs seem to precede their coinciding CNAs and WGDs (Additional file 1: Section B.6). In such cases, we expect a VAF of either $X^B / (X^A + X^B)$ or $X^A / (X^A + X^B)$ depending on which allele the SNV occurred. We defined a log-likelihood ratio (LLR), measuring the relative probability of each truncal SNV’s observed reference and variant reads given the copy numbers predicted by CNRein when compared to each alternative method, with positive values supporting CNRein’s predictions and negative values supporting the method being compared against (Additional file 1: Section A.4.2). As shown in Fig. 3h, Alleloscope has by far the worst statistical support of truncal SNVs (LLR: 12,635.36, $p < 10^{-5}$), followed by CHISEL (LLR: 3418.66, $p < 10^{-5}$) whereas SIGNALS achieved slightly better SNV support than CNRein (LLR: $-1033.42$, $p = 0.0051$). The comparatively small log-likelihood ratio between SIGNALS and CNRein is reflective of general agreement with respect to truncal SNVs.

In summary, for this ovarian cancer sequenced with DLP+ technology, we find that CNRein fits the read depth data and orthogonal SNV data at least as well as all other methods while producing more parsimonious solutions with more shared clonal structure due to its evolution-aware model. In Additional file 1: Section B.5, and Fig. S18, we included additional analyses showing the SNV-based statistical analysis to have some sensitivity to cell-specific CNAs. We also show that read depth supports CNRein’s predicted cell-specific profiles and cell-specific CNAs (Additional file 1: Section B.7, Fig. S19 and Fig. S20). Finally, we performed additional comparisons with modified versions of CNRein, finding CNRein is relatively robust to modifying segment sizes or variance estimates (Additional file 1: Section B.5, Fig. S21 and Fig. S22).

### Evaluation on a breast cancer dataset sequenced with 10x Chromium CNV technology

For further benchmarking, we considered single-cell DNA sequencing data from breast cancer patient S0 sequenced with Chromium Single-Cell Copy Number Variation (CNV) Solution from 10× Genomics. A total of 10,202 cells, distributed across five spatial sections, were sequenced with coverage ranging from $0.01\times$ to $0.05\times$ (Additional file 1: Fig. S23). While both CHISEL and CNRein produced output for all cells, the SIGNALS output consisted of only 3540 cells due to additional filtering criteria, including removal of normal cells as well as high noise cells suspected to be actively replicating, doublets, or otherwise problematic. Additionally, we utilized publicly available predictions from Alleloscope on cells from one particular section of the tumor, amounting to a total of $n=785$ cells for which all methods produced predictions.

**Fig. 4:**
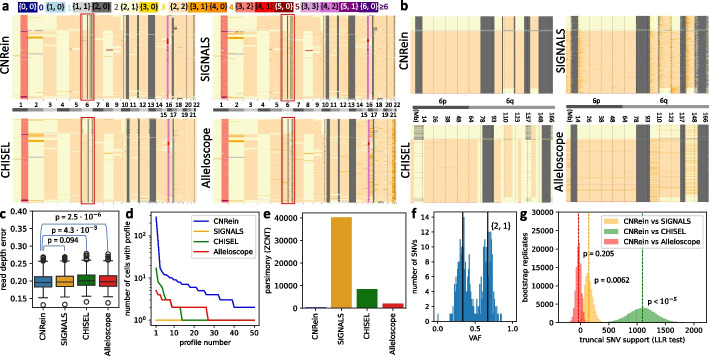
Results on breast cancer patient S0. **a** Allele-specific copy number profiles for CNRein, SIGNALS, CHISEL, and Alleloscope on $n=785$ cells. **b** Allele-specific copy number profiles of chromosome 6, showing that while CNRein retains the ability to detect small CNAs also detected by SIGNALS. **c** All methods have a similar fit to the read depth data, with SIGNALS and CNRein having a slightly better fit. **d** The size of the 50 largest clones for each method, showing much larger clones for CNRein than SIGNALS, CHISEL SIGNALS, and Alleloscope. **e** CNRein’s predictions resulted in a tree with a far lower parsimony score (397) than SIGNALS (40,222), CHISEL (8506), or Alleloscope (2076). **f** VAFs for copy number {2, 1} for CNRein, showing the expected peaks around 1/3 and 2/3. **g** Log likelihood ratios (LLR) of SNVs support on bootstrapped replicates between our method and the alternative methods SIGNALS, CHISEL, and Alleloscope. CNRein outperforms CHISEL (LLR 1093.83 and $p < 10^{-5}$ ) and somewhat outperforms SIGNALS (LLR 145.26 and $p=0.0062$) while being slightly outperformed by Alleloscope (LLR $-35.24$ and $p=0.205$)

In contrast to the DLP+ data, we find a general agreement between CNRein, SIGNALS, and Alleloscope other than additional small (around 1Mb or smaller) CNAs in SIGNALS predictions (Fig. 4a and Additional file 1: Fig. S24) and CHISEL predicting a smaller quantity of LOH, as can be seen in chromosome 6 (Fig. 4b). Looking at all $n=785$ cells and across the entire genome, we find CNRein had a statistically significant closer fit to the read depth data than CHISEL or Alleloscope, while having no statistically significant difference in fit relative to SIGNALS (Additional file 1: Section B.7 and Fig. 4c). Despite fitting the read depth data at least as well as other methods, CNRein identified large sets of cells with identical copy number profiles (see Additional file 1: Section A.4.1 for details). The largest such set identified by CNRein consists of 276 cells (Fig. 4d). On the other hand, the largest set of cells with identical profiles identified by SIGNALS, CHISEL, and Alleloscope consist of only 1, 17, and 5 cells, respectively. Additionally, CNRein determined fewer unique copy number profiles (206) compared to SIGNALS (785), CHISEL (737), and Alleloscope (747). This lower number is due to CNRein’s use of a shared evolutionary model; indeed, CNNaive, which has the same data processing pipeline and statistic model for the data as CNRein but lacks an evolutionary model, produced 628 unique copy number profiles (Additional file 1: Fig. S25). Similar comparisons on the set of 3540 cells for which CNRein, SIGNALS, and CHISEL were run are provided in Additional file 1: Fig. S26 and Fig. S27.

Additionally, we calculated phylogenies from the copy number profiles and evaluated their parsimony scores (Fig. 4e). We find that CNRein’s phylogeny is far more parsimonious (score: 397) than SIGNALS (40,222), CHISEL (8506), and Alleloscope (2076). This difference in parsimony is visually clear in images of the full trees shown in Additional file 1: Fig. S28, showing long branches near the leaves indicative of many CNAs occurring on individual cells rather than on larger clones. To assess whether CNRein’s increased robustness to spurious CNAs came at the expense of substantially decreased sensitivity to legitimate CNAs, we validated on a subset of 755 likely truncal SNVs (more precisely defined in the “Evaluation details” section). In Fig. 4f, we show CNRein’s VAF plot for copy number {2, 1}, showing agreement of truncal SNVs with this copy number (complete data for all methods shown in Additional file 1: Fig. S29). Using our previously defined statistical test for truncal SNVs, we find that CNRein provided a somewhat better fit than SIGNALS (LLR 145.26 and $p=0.0062$), a substantially better fit than CHISEL (LLR 1093.83 and $p<10^{-5}$), and a slightly worse but not significant fit than Alleloscope (LLR $-35.24$ and $p=0.205$). Additionally in Fig. S30, we show that CNRein, SIGNALS, and CHISEL predicted allelic mirroring on copy number {2, 1}, but this prediction is supported by orthogonal SNV data only for CNRein and SIGNALS.

In summary, similarly to the DLP+ ovarian cancer dataset, we find that for this breast cancer dataset sequenced with 10× CNV technology, CNRein fits the read depth and orthogonal SNV data at least as well as competing methods while outputting copy-number profiles with more shared clonal structure. This is in line with our central premise that one should avoid inferring excessive variation in copy numbers across cells without clear evidence in the data. In addition, we analyzed modified versions of CNRein, finding CNRein is relatively robust to modifying segment sizes or variance estimates (Additional file 1: Section B.5, Fig. S31 and Fig. S32).

### Consistency of CNRein on matched 10× and ACT sequencing samples

**Fig. 5:**
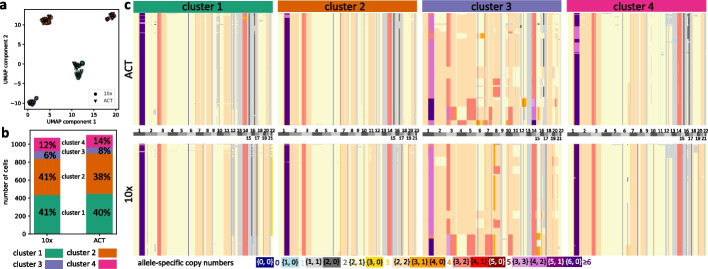
Analysis of breast cancer patient TN3 [29] for both ACT and 10× Chromium technologies. **a** A UMAP of copy number profiles shows 4 clear clusters on pooled copy number profiles for both technologies. **b** Similar proportions for each cluster exist for both technologies. For ACT sequenced cells there are 448, 451, 67, and 135 cells, respectively, in the four clusters, meanwhile for 10× sequenced cells there are 434, 404, 85, and 147 cells, respectively. **c** Copy number profiles in all four clusters are shown for both technologies (with cells in each cluster sorted by average ploidy)

In our final analysis, we assessed whether CNRein is able to produce consistent predictions across multiple sequencing technologies applied to the same tumors. To that end, we analyzed breast cancer patient TN3, which was sequenced using both ACT [29] and 10× sequencing technologies. The coverage for the $n=1101$ cells sequenced with ACT technology ranged from $0.005\times$ to $0.015\times$, whereas the coverage for the $n=1070$ cells sequenced with 10x technology ranged from $0.01\times$ to $0.07\times$ (Additional file 1: Fig. S33). CNRein inferred 119 unique copy number profiles for the ACT cells and 90 profiles for the 10× cells. Strikingly, when projecting the profiles to two dimensions using UMAP and clustering using DBSCAN, we find that the cells cluster into four common groups (Fig. 5a). Moreover, each cluster has a similar proportion of cells for the two technologies (Fig. 5b), with 448 (41%), 451 (41%), 67 (6%), and 135 (12%) cells respectively for the four clusters for ACT sequenced cells and 434 (40%), 404 (38%), 85 (8%), and 147 (14%) cells respectively for 10× sequenced cells. Phylogenies for both sequencing technologies with cells colored by cluster are shown in Additional file 1: Fig. S34, demonstrating general agreement. Copy number profiles for each cluster for the two sequenced technologies are shown in Fig. 5c, showing the similarity between the two technologies for each cluster. Thus, we find that CNRein successfully uncovers similar copy number profiles with similar proportions despite the use of two different sequencing technologies.

## Discussion

We introduced the method CNRein, a deep reinforcement learning based method for haplotype-specific copy number calling. Our central premise is that in the absence of clear signal from input data one should opt to infer shared CNAs to form an evolutionary coherent solution. To that end, CNRein utilizes a generative model of CNA evolution to model the probability of different copy number profiles jointly across cells. We note that our use of reinforcement learning for modeling a generative evolutionary process is conceptually similar to our previous work CloMu [41], an algorithm for modeling SNV evolutionary trees. While CloMu directly models evolutionary tree probabilities, CNRein models the probabilities of trajectories starting on the normal cell and ending on some copy number profile, allowing for a more flexible search while avoiding local minima. We also note that our deep reinforcement learning procedure bears some similarities with GFlowNets [35] such as finding a distribution over trajectories to fit the data rather than maximizing expected rewards.

On simulated data with varying levels of intra-tumor heterogeneity, we found CNRein predicted copy number profiles and numbers of unique copy number profiles that better fit ground truth values than SIGNALS, CHISEL and VICTree. As we do not have access to ground truth copy number profiles in real data and there exists no single perfect evaluation metric, we attempted to assess sensitivity and specificity using several criteria. We evaluated sensitivity to legitimate CNAs with an SNV-based orthogonal validation and fit to read depth. We evaluated specificity, i.e., the ability to avoid spurious CNAs, with parsimony-based scoring of inferred phylogenies. In our evaluation on real data of breast and ovarian cancer, CNRein produced copy number profiles that retained a close fit with read depth but yielded more parsimonious trees and had more realistic numbers of cells with shared profiles (clones) than existing methods. Specifically, CNRein’s findings of extensive heterogeneity in CNA profiles of ovarian cancer OV2295 are in line with previous analyses of high-grade serous ovarian cancer [42–45]. On the other hand, CNRein’s detection of more shared clonal structural in breast cancer S0 is in line with commonly observed punctuated evolution in breast cancer [46]. Additionally, the orthogonal SNV analysis showed CNRein achieved a better fit than CHISEL on both datasets, a much better fit than Alleloscope on ovarian cancer, and similar fits as SIGNALS overall. Finally, we found that CNRein inferred identical clonal structure of a breast cancer patient when run separately on the cells sequenced with 10× and ACT technologies.

Currently, CNRein’s optimization is guided by copy number profiles predicted by CNNaive. Consequently, another useful addition to CNRein would be allowing any copy number profiles to be provided for guiding CNRein, such as the copy number profiles predicted by SIGNALS. Although CNRein’s predictions yield more parsimonious trees than competitor methods, simulations show these parsimony values are still meaningfully higher than the ground truth. Consequently, further improving CNRein in order to produce a maximally parsimonious tree is an important direction for future work. One approach for this could be a post-processing local search to modify CNRein’s CNA tree and obtain improved copy number profiles. For instance, an MCMC optimization approach on CNA trees as performed in SCICoNE [22] could be applied. Another possible modification to our method’s optimization procedure could be adapting GFlowNets towards our problem statement rather than our current procedure of adapting policy gradients. One other natural extension of CNRein would be including the sex chromosomes currently excluded from our analysis.

Future work could also include the ability to detect doublets and S-phase cells. Currently, CNRein produces copy number profiles for such cells, but they are not specifically identified as doublets or S-phase. We also plan on updating our visualization tool [47] to support the interpretation of CNRein results. Utilizing ideas used in the orthogonal validation of CNAs using SNVs [48], we plan on performing integrative inference of SNVs and CNAs, improving upon previous work [49]. Finally, a broader more complex future direction would be allowing CNRein to share a subset of parameters across patients and analyzing a large cohort of patients. One essential advantage of deep learning is its ability to learn complex subtle patterns across large datasets. Consequently, we believe the advantages of CNRein’s deep reinforcement learning could be greatly extended if training parameters across a large cohort of patients were enabled. Such a system could potentially learn general patterns of CNA evolution while also learning the particular evolutionary trajectories of individual patients, akin to methods for identifying evolutionary trajectories of SNVs [41, 50–54].

## Conclusions

Recent low-pass, high-throughput single-cell DNA sequencing technologies enable researchers to characterize intra-tumor heterogeneity across thousands of cells per tumor. In particular these technologies allow one to detect copy-number aberrations (CNAs), a very common and clinically relevant type of somatic mutation in cancer [2–7], using haplotype-specific CNA callers [30–32]. Importantly, current methods to analyze these data may result in spurious CNAs inconsistent with realistic evolutionary constraints. To address this gap, we introduce evolution-aware copy number calling via deep reinforcement learning (CNRein), showing better performance than existing methods on simulated and real data. With the increasing availability of single-cell DNA sequencing data of tumors, we expect that CNRein’s more accurate copy number analyses will enable more precise analyses of intra-tumor heterogeneity.

## Methods

### Problem statement

Given a set of aligned sequencing reads from single-cell DNA sequencing data, we wish to identify a copy number profile $P=[P^{(1)}, P^{(2)}]^\top$ for each cell. These copy number profiles should fit each cells’ read count data well in addition to forming a coherent solution across all cells. The copy numbers that we seek are both allele specific and haplotype specific. That is, each copy number $(P^{(1)}_i,P^{(2)}_{i})$ indicates the number of copies of genetic region *i* of each of the two parental alleles. Additionally, the copy numbers are phased across each chromosome such that copy numbers $(P^{(1)}_{i},P^{(2)}_{i}) = (2, 1)$ and $(P^{(1)}_{j},P^{(2)}_{j}) = (1, 2)$ for two different positions *i* and *j* on the same chromosome indicate two distinct copy numbers. Although the raw input is a set of aligned sequencing reads for each cell, this needs to be further processed to produce the appropriate inputs for copy-number calling. Specifically, we must split the genome into *L* genomic regions, or *segments* such that the copy number is constant in each segment. Then, we determine read counts for each cell and segment, which are corrected for several biases, to produce read depths $\textbf{R} = [R_1, \ldots ,R_N]^\top \in \mathbb {R}^{N \times L}$ such that the read depth of a segment of a cell is proportional to the total copy number, i.e., $R_{s,i} \propto P^{(1)}_{s,i} + P^{(2)}_{s,i}$ Additionally, heterozyous, germline single-nucleotide polymorphisms (SNPs) must be detected, counted, phased into haplotype blocks, and phased across haplotype blocks in order to produce the number of reads matched to each haplotype for each segment. This then produces the B-allele frequencies (BAFs) $\textbf{B} = [B_1, \ldots , B_N]^\top \in [0,1]^{N \times L}$, which measure the proportion of reads coming from the second haplotype (typically set to be the haplotype with a lower average copy number in that chromosome), i.e., $B_{s,i} \approx P^{(1)}_{s,i} / (P^{(1)}_{s,i} + P^{(2)}_{s,i})$. We provide more details on determining the *L* segments and inputs **R** and **B** in Additional file 1: Section A.1.3.

Our measurements **R** and **B** not only depend on the copy number but include additional uncertainty specific to the cell and segment being considered. For instance, GC bias as well the number of germline SNPs in a segment can affect the variance in read depth and BAF, respectively, across segments (Additional file 1: Fig. S2). Additionally, differing coverage across cells can affect the variance across cells. Consequently, we define $\varvec{\Sigma }^R = [\Sigma ^R_1, \ldots , \Sigma ^R_N]^\top \in \mathbb {R}^{N \times L}$ as the cell and segment-specific variances for the read depths $\textbf{R} = [R_1,\ldots ,R_n]^\top$, and $\varvec{\Sigma }^B = [\Sigma ^B_1, \ldots , \Sigma ^B_N]^\top$ as the cell and segment-specific variances for the BAFs $\textbf{B} = [B_1, \ldots , B_N]^\top$. In Additional file 1: Section A.1.3, we describe how we estimate these values. Given these mean and variance values, we define $\Pr (R_s \mid \Sigma ^{R}_s, P )$ as the probability of the observed read depths $R_s$ given variances $\Sigma ^{R}_s$ and copy number profile *P* of a cell *s*. Similarly, we define $\Pr (B_s \mid \Sigma ^{B}_s, P)$ as the probability of the observed BAFs $B_s$ given the variances $\Sigma ^{B}_s$ and the copy number profile *P*. Precise definitions of the Gaussian probability distributions are given below and explanatory details are provided in Additional file 1: Section A.2.1.

1 $$\begin{aligned} \Pr (R_s \mid \Sigma ^{R}_s, P ) & = \prod \limits _{i=1}^L \frac{1}{ \sqrt{\Sigma ^{R}_{s,i} 2 \pi } } \exp \left( \frac{ -(R_{s,i} - c \cdot (P^{(1)}_{i} + P^{(2)}_{i}))^2 }{2 \Sigma ^{R}_{s,i} } \right) ,\end{aligned}$$

2 $$\begin{aligned} \Pr (B_s \mid \Sigma ^{B}_{s,i}, P ) & = \prod \limits _{i=1}^L \frac{1}{ \sqrt{ \Sigma ^{B}_{s,i} 2 \pi } } \exp \left( \frac{ -(B_{s,i} - \frac{P^{(2)}_{i}}{P^{(1)}_{i} + P^{(2)}_{i}} )^2 }{2 \Sigma ^{B}_{s,i} } \right) . \end{aligned}$$

This gives the below equation for the observed data given a copy number profile *P*.

3 $$\begin{aligned} \Pr (R_s, B_s \mid \Sigma ^R_s,\Sigma ^B_s, P) = \Pr (R_s \mid \Sigma ^{R}_s, P ) \Pr (B_s \mid \Sigma ^{B}_s, P ). \end{aligned}$$

**Fig. 6:**
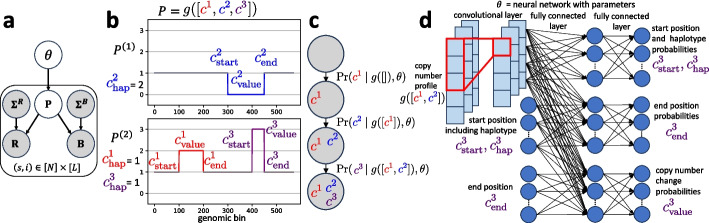
Overview of the CNRein algorithm. **a** The probability of read depths **R** and BAF values **B** is determined by the variance levels $\varvec{\Sigma }^{\textbf{R}}$ and $\varvec{\Sigma }^{\textbf{R}}$ as well as the copy number profile **P**. The probability of copy number profiles **P** then comes from the model parameters $\theta$. **b** A potential copy number profile $g([ c^1, c^2, c^3 ])$ generated by applying mutations $c^1$, $c^2$, and $c^3$ to a normal cell is shown. Specifically, the copy numbers for haplotype 1 and haplotype 2 are plotted and for each CNA tuple, the haplotype number, the start position, the end position, and the value are shown in the copy number profile. **c** In CNRein’s evolutionary model, a copy number profile is generated by the application of a series of CNAs such that the probability of each new CNA depends on the previous copy number profile. In this example, CNRein generates the copy number profile in panel b by sequentially applying the mutations $c^1$, $c^2$, and $c^3$ to a normal cell. **d** The probability of CNA events are determined by a neural network. $g([c^1, c^2])$ is the copy number profile generated by applying the two CNA tuples $c^1$ and $c^2$ to the normal cell. Then, the probability of the components of CNA tuple $c^3$ is generated. Ultimately, $\Pr (c^3 \mid g([c^1, c^2]), \theta )$ is the probability of the next CNA $c^3$ given that CNAs $c^1$ and $c^2$ have already been applied

While previous works infer a copy number profile $P_s$ of each cell *s* in isolation, here we specifically account for the joint evolution of all copy number profiles $\textbf{P} = [P_1,\ldots ,P_N]$. As such, we define $\Pr ( P \mid \theta )$ as the probability of the copy number profile *P* according to a model with tumor-specific parameters $\theta$. For our method CNRein, the parameters $\theta$ are the weights and biases of our deep neural network which we use in combination with an evolutionary model to calculate $\Pr (P \mid \theta )$. We define $\mathcal {P}$ as the set of all possible haplotype-specific copy number profiles with *L* segments. In practice, $\mathcal {P}$ will be bounded by setting a maximum per-haplotype copy number of $C_{\textrm{max}}$ (set to a default value of 19). As shown on the plate diagram in Fig. 6a, the probability of read depths $R_s$ and BAFs $B_s$ are derivable from $\varvec{\Sigma }^R$, $\varvec{\Sigma }^B$, and $\theta$. The probability of observed read depths $R_s$ and BAFs $B_s$ is

4 $$\begin{aligned} \Pr (R_s, B_s \mid \varvec{\Sigma }^R, \varvec{\Sigma }^B, \theta ) = \sum \limits _{P \in \mathcal {P}} \Pr (P \mid \theta ) \Pr (R_s, B_s \mid \Sigma ^R_s,\Sigma ^B_s, P). \end{aligned}$$

The probability of the read depths $\textbf{R} = [R_1, \ldots ,R_N]^\top$ and the BAFs $\textbf{B} = [B_1, \ldots ,B_N]^\top$ for *N* cells is then

5 $$\begin{aligned} \Pr (\textbf{R}, \textbf{B} \mid \varvec{\Sigma }^R, \varvec{\Sigma }^B, \theta ) = \prod \limits _{s=1}^N \Pr (R_s, B_s \mid \Sigma ^R_s, \Sigma ^B_s, \theta ). \end{aligned}$$

This leads to the following problem.

#### Problem 1

(Copy Number Profile Distribution). Given read depths $\textbf{R} = [R_1, \ldots ,R_N]^\top$ and B-allele frequencies $\textbf{B} = [B_1, \ldots , B_N]^\top$ and their variances $\varvec{\Sigma }^R$ and $\varvec{\Sigma }^B$ for *N* cells and *L* segments find the model parameters $\theta$ that maximize $\Pr (\textbf{R}, \textbf{B} \mid \varvec{\Sigma }^R, \varvec{\Sigma }^B, \theta )$.

Once we identify the parameters $\theta$ that maximize the probability of the observed measurement data, it is relatively straightforward to find a predicted profile $P_s$ for each cell *s* such that

6 $$\begin{aligned} P_s = \textrm{argmax}_{P \in \mathcal {P}} \Pr ( P \mid \theta ) \Pr ( R_s, B_s \mid \Sigma ^R_s,\Sigma ^B_s, P), \end{aligned}$$

provided one can sample copy number profiles *P* from $\Pr ( \cdot \mid \theta )$.

### CNRein algorithm details

#### Evolutionary model for computing profile probabilities

To solve the Copy Number Profile Distribution problem (Problem 1), we define an evolutionary model for probabilistically generating copy number profiles. The model is trained on each patient independently, and so trained model parameters $\theta$ describe the evolution of a single cancer and capture the probability of different copy number profiles within that tumor. The copy number profile of a normal cell is $P^{(1)}_i = P^{(2)}_{i} = 1$ for all segments $i \in [L] = \{1, \ldots ,L\}$. In our model, a tumor first starts with a single clone with the copy number profile of a normal cell. Then, a CNA occurs producing a new clone. The new clone has a copy number profile in which the values in either $P^{(1)}$ or $P^{(2)}$ are modified to new values for some interval of segments within a chromosome. This interval is allowed to overlap with other existing CNAs. Alternatively, a whole genome duplication can occur in which all copy numbers are doubled. In either case, this produces a new copy number profile which can then be modified by additional CNAs. This process repeats until terminating on some final CNA. As an example, if segments 30 through 60 are within some chromosome, a CNA could be amplifying $P^{(1)}$ in segments 35 to 50 to the value 2, i.e., $P^{(1)}_{i} = 2$ for segments $35 \le i \le 50$.

To model the evolution of CNAs, we define a *CNA tuple* $c = ( c_{\textrm{WGD}}, c_{\textrm{hap}}, c_{\textrm{start}}, c_{\textrm{end}}, c_{\textrm{value}})$. The value of $c_{\textrm{WGD}}$ is 1 if the mutation is a whole genome duplication and 0 otherwise. Assuming $c_{\textrm{WGD}}= 0$, then $c_{\textrm{hap}} \in \{1,2\}$ is the haplotype number of the CNA, $c_{\textrm{start}} \in [L]$ is the starting segment, $c_{\textrm{end}} \in [L]$ is the ending segment, which must be on the same chromosome as the starting segment, and $c_{\textrm{value}} \in \mathbb {Z}$ indicates the change in the copy number. Let $\mathcal {C}$ be the set of all such possible CNA tuples (with the restriction $-5 \le c_{\textrm{value}} \le 5$). We define a *generating sequence* *G* as any list of CNA tuples $[c^1, \ldots ,c^k] \subseteq \mathcal {C}$. We define *g*(*G*) as the copy number profile generated by applying the CNAs in *G* to the copy number profile of a normal clone. An example of a copy number profile being generated by a sequence of CNA tuples is shown in Fig. 6b, c.

To precisely define *g*(*G*), we first define a function $f_g(P, [c])$, which takes as input a copy number profile *P* and a single CNA tuple $c=(c_{\textrm{WGD}}, c_{\textrm{hap}}, c_{\textrm{start}}, c_{\textrm{end}}, c_{\textrm{value}})$ and outputs a new copy number profile $f_g(P, [c] ) = [Q^{(1)},Q^{(2)}]^\top$ such that

7 $$\begin{aligned} Q^{(1)}_i = \left\{ \begin{array}{ll} 2P^{(1)}_i, & \text {if}\ c_{\textrm{WGD}} = 1,\\ P^{(1)}_i, & \text {if}\ c_{\textrm{WGD}} = 0\ \text {and}\ c_{\textrm{hap}} = 2,\\ P^{(1)}_{i}, & \text {if}\ c_{\textrm{WGD}} = 0, c_{\textrm{hap}} = 1\ \text {and}\ i \not \in \{c_{\textrm{start}}, \ldots , c_{\textrm{end}}\},\\ P^{(1)}_{i} + c_{\textrm{value}}, & \text {if}\ c_{\textrm{WGD}} = 0, c_{\textrm{hap}} = 1\ \text {and}\ i \in \{c_{\textrm{start}}, \ldots , c_{\textrm{end}}\},\\ \end{array}\right. \end{aligned}$$

and

8 $$\begin{aligned} Q^{(2)}_i = \left\{ \begin{array}{ll} 2P^{(2)}_i, & \text {if}\ c_{\textrm{WGD}} = 1,\\ P^{(2)}_i, & \text {if}\ c_{\textrm{WGD}} = 0\ \text {and}\ c_{\textrm{hap}} = 1,\\ P^{(2)}_{i}, & \text {if}\ c_{\textrm{WGD}} = 0, c_{\textrm{hap}} = 2\ {\textbf {and}}\ i \not \in \{c_{\textrm{start}}, \ldots , c_{\textrm{end}}\},\\ P^{(2)}_{i} + c_{\textrm{value}}, & \text {if}\ c_{\textrm{WGD}} = 0, c_{\textrm{hap}} = 2\ {\textbf {and}}\ i \in \{c_{\textrm{start}}, \ldots , c_{\textrm{end}}\},\\ \end{array}\right. \end{aligned}$$

With this definition, we now inductively define

9 $$\begin{aligned} f_g(P, [c^1, \ldots ,c^k] ) = \left\{ \begin{array}{ll} P, & \text{ if } k=0\text{, }\\ f_g( f_g(P, [c^1, \ldots ,c^{k-1}] ) , [c^k] ), & \text{ if } k \ge 1\text{. } \end{array}\right. \end{aligned}$$

Finally, we define $g(G) = f_g(P_\emptyset , G)$, where $P_\emptyset = [P^{(1)}_\emptyset ,P^{(2)}_\emptyset ]^\top$ is the normal clone with $P^{(1)}_{\emptyset ,i} = P^{(2)}_{\emptyset ,i} = 1$ for each segment *i*.

Given an existing copy number profile *P*, model parameters $\theta$ can assign a probability to each new CNA tuple $c \in \mathcal {C}$ as well as a probability that no new CNAs will occur. We define this probability as $\Pr ( c \mid P, \theta )$. Additionally, we define $\Pr ( \textrm{stop} \mid P, \theta )$ as the probability that no new CNAs will occur in the cell. In practice, since $\mathcal {C}$ can be extremely large, we first assign a probability to each starting position $c_{\textrm{start}}$ given the existing profile *P*, then assign a probability to each ending position $c_{\textrm{end}}$ given the starting position $c_{\textrm{start}}$ and *P*, and finally assign a probability to the copy number value $c_{\textrm{value}}$ given the start position $c_{\textrm{start}}$, ending position $c_{\textrm{end}}$, and *P*. This is done using a deep convolutional neural network with an architecture shown in Fig. 6d, and described in the “Neural network architecture” section. Using this, we assign a probability to any generating sequence $G = [c^1, \ldots ,c^k]$ of CNA tuples as

10 $$\begin{aligned} \Pr ( G = [c^1, \ldots ,c^{k}] \mid \theta ) = \Pr ( \textrm{stop} \mid g( [c^1, \ldots ,c^{k}] ) , \theta ) \prod \limits _{\ell =1}^k \Pr (c^{\ell } \mid g( [c^1, \ldots ,c^{\ell -1}] ), \theta ). \end{aligned}$$

In the above equation *g* applied to the empty sequence, i.e., *g*([]), results in the normal copy number profile. The process of forming a generating sequence by repeatedly applying CNAs is shown in Fig. 6c. Putting together our definitions, we define the probability for any copy number profile *P* as

11 $$\begin{aligned} \Pr (P \mid \theta ) = \sum \limits _{ G \subseteq \mathcal {C}, g(G) = P } \Pr ( G \mid \theta ). \end{aligned}$$

Note that any $G \subseteq \mathcal {C}$ is a generating sequence. An example generated copy number profile is shown in Fig. 6b. Putting this probability into equation (4) then allows us to solve Problem 1 by optimizing CNA tuple probabilities.

#### Neural network architecture

We use a neural network as a generative model for copy number profiles. Each input copy number profile $P = [P^{(1)}, P^{(2)}]^\top$ first has its values capped at a maximum value of 19. In other words, if $P^{(h)}_{i}> 19$, then we set $P^{(h)}_{i} = 19$. Consequently, each element in $P^{(h)}_{i}$ can take 20 values ($\{0, 1, \ldots 19\}$). This value is one-hot encoded so that *P* becomes an $L \times 2 \times 20$ tensor. This tensor is then reshaped into an $L \times 40$ tensor by concatenating the one-hot encoded copy numbers for both haplotypes for each of the *L* segments. An embedding network then converts this to a 500 dimensional vector. Specifically, the embedding network applies convolutions of size 5 across the bins. There are a total of 400 such convolutions, changing the second dimension of the copy number profile representation from 40 to 10 (going from 40 channels to 10 channels). The copy number profile representation tensor is then flattened, and a hyperbolic tangent non-linearity is applied. Finally, a fully connected layer converts this tensor to a 500 dimensional embedding of the copy number profile. The starting and ending position of each CNA are encoded as one-hot L dimensional vector. A fully connected layer also converts these to 500 dimensional representations. When determining the end position, the embedding of the start position is added to the embedding of the copy number profile. When determining the copy number, the embeddings of both the start and end positions are added to the embedding of the copy number profile. For predicting the start position, the end position, or copy number, a hyperbolic tangent non-linearity is applied followed by a fully connected layer. Additionally, the softmax function is applied to convert the outputs into probabilities. Predicting whether or not to terminate the generating sequence is done in the same manner as predicting the start position.

#### Training: Copy number calling via reinforcement learning

To exactly solve Problem 1, we must maximize $\Pr (\textbf{R}, \textbf{B} \mid \varvec{\Sigma }^R, \varvec{\Sigma }^B, \theta )$, which requires calculating a sum over all $P \in \mathcal {P}$. The set $\mathcal {P}$ of all possible copy number profiles is too large for this to be feasible, so instead we must use sampling. Intuitively, we generate copy number profiles and highly reward profiles that nicely fit the observed data, yielding parameter updates that increase the probability of the observed data given the model. We maximize $\log \Pr (\textbf{R}, \textbf{B} \mid \varvec{\Sigma }^R, \varvec{\Sigma }^B, \theta )$, which is equivalent to maximizing $\Pr (\textbf{R}, \textbf{B} \mid \varvec{\Sigma }^R, \varvec{\Sigma }^B, \theta )$ while allowing for useful mathematical manipulations. To achieve this maximization, we calculate the gradient of $\log \Pr (\textbf{R}, \textbf{B} \mid \varvec{\Sigma }^R, \varvec{\Sigma }^B, \theta )$ with respect to $\theta$. As derived in Additional file 1: Section A.3.1, we have the equation

12 $$\begin{aligned} \frac{d}{d\theta } \log \Pr (\textbf{R}, \textbf{B} \mid \varvec{\Sigma }^R, \varvec{\Sigma }^B, \theta ) = \mathbb {E}_{G \sim \Pr ( \cdot \mid \theta )} \left[ \sum \limits _{s=1}^N \Pr (R_s, B_s \mid \theta )^{-1} \Pr (R_s, B_s \mid G) \frac{d}{d\theta } \log (\Pr (G \mid \theta ))\right] . \end{aligned}$$

In the above equation and in the following text, we use the shorthands $\Pr (R_s, B_s \mid G ) = \Pr (R_s, B_s \mid \Sigma ^R_s,\Sigma ^B_s ,g(G) )$ and $\Pr (R_s, B_s \mid \theta ) = \Pr (R_s, B_s \mid \Sigma ^R_s,\Sigma ^B_s, \theta )$. We define the reward function as

13 $$\begin{aligned} r(G) = \sum \limits _{s=1}^N \Pr (R_s, B_s \mid \theta )^{-1} \Pr (R_s, B_s \mid G). \end{aligned}$$

Intuitively, this function rewards trajectories that result in profiles that closely fit cells’ read count data as indicated by $\Pr (R_s, B_s \mid G)$. Additionally, more reward is given if those cells do not yet have high probability profiles that fit them well as indicated by $\Pr (R_s, B_s \mid \theta )^{-1}$. Without the term $\Pr (R_s, B_s \mid \theta )^{-1}$, reinforcement learning would result in 100% probability given to the single best copy number profile rather than finding a set of copy number profiles that fit all cells. Estimating $\Pr (R_s, B_s \mid \theta )$ for the reward function is accomplished via sampling as described in Additional file 1: Section A.3.2. A theoretical alternative to avoid calculating this normalization factor via sampling would be to use GFlowNets [35]. However, GFlowNets optimize for terminal state probabilities being proportional to rewards which would not allow for our problem statement of maximizing the overall probability of the observed dataset (additional details in Additional file 1: Section A.3.8).

With this reward function, we have a standard policy learning gradient [55] (assuming a reward is only given at the last time step)

14 $$\begin{aligned} \mathbb {E}_{G \sim \Pr ( \cdot \mid \theta ) } [r(G) \frac{d}{d\theta } \log (\Pr (G \mid \theta )) ]. \end{aligned}$$

Although this all could work in theory, solving this problem from scratch requires significant computational resources and may require substantial tuning of hyperparameters on each new dataset in order to ensure successful optimization. This would be acceptable if the goal of this paper was to successfully estimate a copy number profile for a single cancer. However, the goal is instead to provide a robust and fast tool for estimating copy number profiles in general. To do so, we guide the optimization with an initial set of copy number profiles $\tilde{\varvec{P}}$ provided by CNNaive and utilize importance sampling [56]. A simplified version of guiding the optimization with CNNaive’s predicted copy number profiles is described in Additional file 1: Section A.3.3 and modifications to improve CNRein’s accuracy are described in Additional file 1: Section A.3.4 and Section A.3.5. Additional mathematical details are provided in Additional file 1: Section A.3.6 and Section A.3.7.

### Evaluation details

#### Evaluation metrics for simulations

CNRein’s reinforcement learning optimization was run on a laptop with 96GB of RAM and a 3.6 GHz processor (with 12 cores), without the use of a GPU for all experiments. On 20 simulation instances we compared predicted copy number profiles with the ground truth copy number profiles. To do so, we compare the predictions of each method with the ground truth on 100kb bins. Since each method may yield different segmentations, we compare the predictions of each method with the ground truth on the fixed-size 100kb bins. Since we do not simulate the complexities of linkage disequilibrium and haplotype blocks and instead generate already-phased inputs, we define accuracy using unordered allele-specific copy numbers rather than distinguishing between maternal and paternal haplotypes. Specifically, we defined the accuracy as the average percentage of bins across cells with the unordered allele-specific copy number predicted exactly correctly. To be more precise, let

15 $$\begin{aligned} \delta _{\text {acc}}(a, b, a', b') = \left\{ \begin{array}{ll} 1, & \text {if}\ \max (a, b) = \max (a', b')\ \text {and}\ \min (a, b) = \min (a' b'),\\ 0, & \text {otherwise.} \end{array}\right. \end{aligned}$$

Let $P_1, \ldots P_N$ be the predicted copy number profiles and $\overline{P}_1, \ldots , \overline{P}_N$ be the true copy number profiles for *N* cells across *K* fixed-size bins in some simulation. Then the *accuracy* is as below

16 $$\begin{aligned} \frac{1}{NK} \sum \limits _{s=1}^N \sum \limits _{i=1}^K \delta _{\text {acc}}(P^{(1)}_{s,i}, P^{(2)}_{s,i}, \overline{P}^{(1)}_{s,i}, \overline{P}^{(2)}_{s,i}). \end{aligned}$$

An alternative error metric is the L1 error. Let

17 $$\begin{aligned} \delta _{\text {L1}}(a, b, a', b') = |\max (a, b) - \max (a', b')| + |\min (a, b) - \min (a', b')|. \end{aligned}$$

Then, the *L1 error* metric is defined as

18 $$\begin{aligned} \frac{1}{NK} \sum \limits _{s=1}^N \sum \limits _{i=1}^K \delta _{\text {L1}}(P^{(1)}_{s,i}, P^{(2)}_{s,i}, \overline{P}^{(1)}_{s,i}, \overline{P}^{(2)}_{s,i}). \end{aligned}$$

#### Inferring copy number phylogenies via maximum parsimony

On both simulated and real data, we generated phylogenies for each method and evaluated their parsimony values. Specifically, we used the Lazac algorithm [40] for computing trees and phylogenies, which utilizes the zero-agnostic copy number transformation (ZCNT) distance [40] .

#### Orthogonal SNV validation on real data

On real data, we performed validation by utilizing orthogonal SNV data. For ovarian cancer patient OV2295, we used the set of SNV reported in the paper [27]. For breast cancer patient S0, we ran Mutect2 [57] on two pseudobulk samples generated from the single-cell data, one composed of normal cells (4239 cells) and one composed of tumor cells (5963 cells), identifying 3044 SNVs using standard filtering criteria. We restricted to likely truncal SNVs for all analyses. Specifically, for ovarian cancer patient OV2295, we restricted to SNVs that were present in at least 5 cells for all three cell lines. For breast cancer patient S0, we restricted to SNVs present in at least 5 cells for at least 4 out of the 5 sections (noting that one section consists primarily of non-tumor cells).

We then generated VAF plots which showed consistency with truncal SNVs typically occurring prior to CNAs (Additional file 1: Section B.6). Specifically for any copy number $\{X^A, X^B\}$ we saw peaks in the VAF around $X^B / (X^A + X^B)$ and $X^A / (X^A + X^B)$ rather than other integer multiples of $1/(X^A+X^B)$, indicating that the SNV occurs on all copies of one of the two haplotypes. We utilized this tendency to produce a log-likelihood ratio test that measures the relative probability of an SNV’s observed (reference and variant) reads given the copy numbers predicted by CNRein when compared to each alternative method on all cells.

We provide a brief description of the test here with full mathematical details in Additional file 1: Section A.4.2. The probability of a single read being a variant read (as opposed to a reference read) for an SNV on haplotype A for copy number $(X^A, X^B)$ is $X^A / (X^A + X^B)$. A similar calculation gives these values for reference reads as well as for SNVs that occur on haplotype B. Utilizing these, we calculate the probability of all of the reads for a given SNV assuming it occurred on haplotype A, and the probability of all reads for that SNV assuming it occurred on haplotype B. Since we do not know which haplotype each SNV occurred on, we take the maximum of these two values to estimate the probability of the observed (variant and reference) reads of a given SNV (given the copy numbers of each cell in the position of the SNV). Taking the log of the probability of an SNV given the predictions of CNRein and subtracting the log of the probability of that SNV given the predictions of an alternative method gives a log-likelihood ratio for a single SNV. Summing across all truncal SNVs gives a cumulative measurement for truncal SNV support, with positive values supporting CNRein’s predictions and negative values supporting the method being compared against. Finally, we performed bootstrapping on the set of truncal SNVs to obtain statistical bounds. It is worth noting that there are certain copy numbers that this SNV-based test can not differentiate. Namely, the copy numbers $(X_1, X_2)$ and $(Y_1, Y_2)$ can not be differentiated if they differ by a constant factor *c* such that $X_2 = c X_1$ and $Y_2 = c Y_1$.

## Supplementary Information

Additional file 1. Supplemental figures and text.

Additional file 2. Review history.

## Data Availability

Ovarian cancer patient OV2295 [27] has single-cell FASTQ files available in the European Genome-phenome Archive under accession number EGAS00001003190 [58]. Breast cancer patient S0 is available at https://support.10xgenomics.com/single-cell-dna/datasets) [59]. Breast cancer patient TN3 [29] has FASTQ files for both ACT and 10× sequencing technologies available in the NCBI Sequence Read Archive under accession number PRJNA629885 [60]. Simulated data generated in this paper are publicly available at https://github.com/elkebir-group/CNRein [61] as well as on Zenodo [62] (DOI: https://doi.org/10.5281/zenodo.14946943) under the BSD 3-clause license. CNRein is implemented in Python using PyTorch and is available at https://github.com/elkebir-group/CNRein and Zenodo (DOI: https://doi.org/10.5281/zenodo.14946943) under the BSD 3-clause license.
