# Supplementary material for: CNRein: an evolution-aware deep reinforcement learning algorithm for single-cell DNA copy number calling
Source: Genome Biol. 2025 Apr 7;26:87. doi: 10.1186/s13059-025-03553-2 (PMC11974095; doi:10.1186/s13059-025-03553-2)
Supplement: Supplementary file 1 — Additional file 1. Supplemental figures and text. [file 13059_2025_3553_MOESM1_ESM.pdf]

Supplementary Materials: CNRein: an evolution-aware deep reinforcement learning algorithm for single-cell DNA copy number calling

Stefan Ivanovic[1] and Mohammed El-Kebir[1,2,†]

[1]Department of Computer Science, University of Illinois at Urbana-Champaign, IL 61801, USA

[2]Cancer Center Illinois, University of Illinois at Urbana-Champaign, IL 61801, USA

[†]Correspondence: melkebir@illinois.edu

Contents

A Supplementary methods **5**

A.1 Data processing . . . 5

A.1.1 Processing raw read counts . . . 5

A.1.2 Determining haplotype-specific read counts . . . 6

A.1.3 Estimating segments and noise levels . . . 10

A.2 CNNaive . . . 14

A.2.1 Defining measurement probabilities . . . 14

A.2.2 Estimating cell-specific scaling factor . . . 15

A.2.3 The predicted profiles of CNNaive . . . 18

A.3 CNRein . . . 19

A.3.1 Policy learning equation derivation . . . 19

A.3.2 Probability estimation via sampling . . . 20

A.3.3 Guiding the optimization with initial copy number profiles . . . 21

A.3.4 A modification to improve the removal of spurious CNAs . . . 22

A.3.5 Modifying the search space of copy number profiles . . . 22

A.3.6 Efficient generative sequences . . . 23

A.3.7 Restricted sampling probabilities 25
A.3.8 Comparison of our reinforcement learning method to existing approaches 25
A.4 Post-processing and validation 26
A.4.1 Determining clone sizes when not all cells have values for all bins 26
A.4.2 Calculating the probability of SNV counts given copy numbers 27
A.4.3 Simulation set-up 29

B Supplementary results **31**
B.1 VICTree and CHISEL with clonal predictions 31
B.2 CNAsim simulations 32
B.3 Detection of small CNAs 34
B.4 Accuracy in detecting small clones 35
B.5 Benchmarking modifications of CNRein 36
B.6 VAF analysis on additional copy numbers 38
B.7 Comparing copy numbers with read depth 39

List of Figures

S1 A diagram of the processes in CNRein 43
S2 A comparison of breast cancer patient S0 read depths and our simulated read depths 44
S3 Runtimes on 20 simulation instances for CNRein and CNNaive 45
S4 The error of CNRein and CNNaive on small, medium, and large CNAs on simulated data . . 45
S5 CNRein accurately reconstructs the number of cell-specific profiles on simulated data 46
S6 CNRein accurately reconstructs small clones on simulations 47
S7 Testing VICTree and clonal CHISEL on simulations 48
S8 Results on CNAsim simulations 49
S9 The coverage of cells of ovarian cancer patient 49
S10 L1 differences between copy number profiles predicted by CNRein, SIGNALS, CHISEL, and Alleloscope on ovarian cancer patient OV2295 50
S11 Full trees of each method for ovarian cancer patient OV2295 51
S12 VAF values for common copy numbers for ovarian cancer patient OV2295 52
S13 A diagram of how allelic mirroring affects VAFs 53

S14 VAFs demonstrating allelic mirroring for copy number $\{2, 1\}$ on ovarian cancer patient OV2295 . . . 53
S15 VAFs of truncal SNVs overlapping with various copy numbers inferred by CNRein on ovarian cancer patient OV2295 . . . 54
S16 VAFs of truncal SNVs overlapping with various copy numbers inferred by SIGNALS on ovarian cancer patient OV2295 . . . 55
S17 VAFs of truncal SNVs overlapping with various copy numbers inferred by CHISEL on ovarian cancer patient OV2295 . . . 56
S18 Truncal SNV analysis of CNRein post processed to remove cell-specific copy number profiles on ovarian cancer patient OV2295 . . . 56
S19 Cells with predicted cell-specific profiles have more distinct read depths . . . 57
S20 Distributions of scaled read depth for cell-specific CNAs with different total copy numbers . 57
S21 CNRein with incorrect variances on ovarian cancer patient OV2295 . . . 58
S22 CNRein predictions with modified segment sizes on ovarian cancer patient OV2295 . . . 58
S23 The coverage of cells of breast cancer patient S0 . . . 59
S24 L1 differences between copy number profiles predicted by CNRein, SIGNALS, CHISEL, and Alleloscope on breast cancer patient S0 . . . 59
S25 The number of cells for CNNaive for common copy number profiles on breast cancer patient S0 . . . 60
S26 A heatmap of allele-specific copy number predictions on 3,540 cells from breast cancer patient S0 . . . 60
S27 Clone sizes and parsimony on on 3,540 cells from breast cancer patient S0 . . . 60
S28 Full trees of each method on breast cancer patient S0 . . . 61
S29 VAFs of truncal SNVs overlapping with three common copy numbers for CNRein SIGNALS and CHISEL on breast cancer patient S0 . . . 62
S30 VAFs demonstrating allelic mirroring for copy number $\{2, 1\}$ on breast cancer patient S0 . . 62
S31 CNRein with incorrect variances on breast cancer patient S0 . . . 63
S32 CNRein predictions with modified segment sizes on ovarian cancer patient OV2295 . . . 63
S33 The coverage of cells of breast cancer patient TN3 . . . 64
S34 Phylogenies for breast cancer patient TN3 for both sequencing technologies . . . 64
S35 An example of a chromosome requiring our adjusted phasing algorithm . . . 65

List of Tables

S1 Glossary of notation 5
S2 Comparison of our reinforcement learning approach with existing approaches 26

notation	meaning
$\mathbf{R}$	the read depth
$\mathbf{B}$	the BAF (B-allele frequency)
$\mathbf{\Sigma^R}$	the estimated variance in the read depth
$\mathbf{\Sigma^B}$	the estimated variance in the BAF
$\mathbf{A}$	the read counts for both alleles
P	a copy number profile
θ	the parameters of our neural network model
c	a CNA (copy number abberation)
G	a generative sequence of CNAs
$r(G)$	the reward for the G
$g(G)$	the copy number profile generated by G
s	used to index cells
i or j	used to index segments
$P^{(1)}$	the first allele of the copy number profile P
$P^{(2)}$	the second allele of the copy number profile P
N	the number of cells
K	the number of initial 100kb bins
L	the number of segments

Table S1: Glossary of notation

A Supplementary methods

In Section A.1 we discuss our data processing steps, followed by a description of CNNaive (Section A.2) and then CNRein (Section A.3). Fig. S1 gives a diagram of these processes in the algorithm. Table S1 provides a glossary of our notation.

A.1 Data processing

A.1.1 Processing raw read counts

We calculated read counts for bins of size ℓ_{bin} (set to $\ell_{\text{bin}} = 100$kb by default). To do so, we removed duplicate reads and reads with a very low mapping quality (below 40) We then removed bins with a mappability below 0.8, which removes telomeres and centromeres. Additionally, we remove bins with extreme outlier

high read counts. To do so, we calculate the mean and standard deviation of the bottom 99% of bins by read count. Then, outlier bins with read depths above 6 standard deviations above the mean are removed.

The number of reads in a bin is not only a function of the copy number of that bin but also its GC content due to biases in Illumina sequencing, and the bin's mappability. We calculated the *GC content* of these bins, which is defined as the proportion of bases that are either guanine (G) or cytosine (C), and utilized existing estimates of mappability. We then applied LOWESS regression to correct read depths for GC bias and mappability bias. Specifically, we first predict the read depth from mappability and then divide by this value. Then for each cell, we predict the read depth from the GC content and again divide the read depth by this value. This type of bias correction utilizing LOWESS regression is common in copy number calling methods such as CHISEL [1]. After we removed outlier bins and applied GC bias correction, we obtained a read depth vector $\tilde{R}_s \in \mathbb{R}^K_{\geq 0}$ for each cell s where K is the number of 100kb bins after filtering. Additionally for mathematical convenience, we scaled $\tilde{R}_s$ to have an average value of 1 across all bins for each cell s. For our real data, the number K of bins was $26{,}033$ for ovarian cancer patient OV2295, $26{,}135$ for breast cancer patient S0, $26{,}041$ and $26{,}141$ for breast cancer patient TN3 sequenced with ACT and 10x technologies.

A.1.2 Determining haplotype-specific read counts

To be able to infer allele-specific and haplotype-specific copy numbers, we determined the numbers $A^{(1)}_{s,i}, A^{(2)}_{s,i} \in \mathbb{N}$ of reads in each bin i and cell s that can be mapped to the two parental haplotypes, respectively. To do so, we first pooled all of the single-cell BAM files together into a pseudobulk sample. Then, we ran bcftools' (1.9) [2] `mpileup` and `call` commands on this pseudobulk sample to obtain SNP positions and alleles. We then ran SHAPE-IT 4 [3] in combination with the 1000 genomes reference panel [4] to phase these SNPs into haplotype blocks. Next, we reapplied bcftools' `mpileup` and `call` commands to the individual cells in the positions of these phased SNPs to determine the SNP counts for individual cells. We then converted these to cumulative counts for the A allele and B allele in haplotype blocks for each cell. Finally, we phased these haplotype blocks with a novel algorithm with several theoretical advantages including the ability to phase haplotype blocks when the average copy number for the two alleles is balanced across cells. We discuss this algorithm in the following.

Description of the phasing algorithm: The simplest way of phasing haplotype blocks is to simply define the allele with the lower count across all cells in any haplotype block to be the B allele. However, as noted in the SIGNALS paper [5], this may be ineffective in the case where only a small subset of cells have an

imbalanced copy number between the two alleles. This is because in that case, imbalances in allele counts due to noise in cells with a balanced copy number may overwhelm imbalances in the small number of cells with imbalanced copy numbers. Additionally, it is possible for the average copy number across cells to be balanced despite individual cells having an imbalanced copy number. For instance, if some cells have a gain on one allele and other cells have a gain on the other allele. To overcome these challenges, we begin by describing a simple algorithm that would work well assuming the copy number is constant across each chromosome (but differs across cells). Then, we will describe a modification to this algorithm that allows for accurate haplotype phasing without that assumption.

Running SHAPE-IT yields a partition of the reference genome into haplotype blocks. Let $C^A_{s,\eta}$ and $C^B_{s,\eta}$ be the number of counts of the first and second allele respectively, for cell s and haplotype block η (in some fixed chromosome). Note that the first and second alleles are defined arbitrarily since the haplotype blocks are not yet phased. If there is no imbalance, then read counts on either allele are equally likely and so the counts $C^A_{s,\eta}$ and $C^B_{s,\eta}$ come from a binomial distribution with $p = 1/2$. Thus, assuming no imbalance, the probability of observing the values $C^A_{s,\eta}$ and $C^B_{s,\eta}$ is

$$\binom{C^A_{s,\eta} + C^B_{s,\eta}}{C^A_{s,\eta}} \frac{1}{2^{C^A_{s,\eta}+C^B_{s,\eta}}}. \tag{1}$$

Given this distribution, the imbalance quantity $C^A_{s,\eta} - C^B_{s,\eta}$ has a mean of zero and a variance equal to the total number of counts $(C^A_{s,\eta} + C^B_{s,\eta})$. Additionally, this binomial distribution can be closely approximated with a Gaussian distribution where the imbalance quantity $C^A_{s,\eta} - C^B_{s,\eta}$ has a mean of zero and a variance equal to the total number of counts $(C^A_{s,\eta} + C^B_{s,\eta})$. Consequently, dropping constant terms, observing the imbalance $C^A_{s,\eta} - C^B_{s,\eta}$ has a probability proportional to $\exp(-\frac{1}{2}(C^A_{s,\eta} - C^B_{s,\eta})^2(C^A_{s,\eta} + C^B_{s,\eta})^{-1})$. Thus, the log probability evidence for imbalance is $(C^A_{s,\eta} - C^B_{s,\eta})^2(C^A_{s,\eta} + C^B_{s,\eta})^{-1}$ (again dropping a constant factor). Including the sign of the imbalance (which is necessary to keep track of for phasing haplotype blocks) gives $(C^A_{s,\eta} - C^B_{s,\eta})|C^A_{s,\eta} - C^B_{s,\eta}|(C^A_{s,\eta} + C^B_{s,\eta})^{-1}$. Define

$$M_{s,\eta} = \frac{(C^A_{s,\eta} - C^B_{s,\eta})|C^A_{s,\eta} - C^B_{s,\eta}|}{(C^A_{s,\eta} + C^B_{s,\eta})}. \tag{2}$$

Intuitively, $|M_{s,\eta}|$ is proportional to the negative log probability of the absence of an imbalance towards the direction sign$(M_{s,\eta})$. In other words, if $|M_{s,\eta}|$ is large then there is very strong evidence of imbalance toward A allele if $M_{s,\eta} > 0$ and the B allele if $M_{s,\eta} < 0$.

For any two haplotype blocks η and η' in the same chromosome, we want to phase the haplotypes such that the evidence for imbalance in any cell s points in the same direction for both haplotype blocks. In other words, we want to phase the two haplotype blocks such that if cell s has strong evidence for an

imbalance in favor of the A allele in haplotype η then it should also be imbalanced in favor of the A allele in haplotype block η'. Mathematically, we want $M_{s,\eta} \cdot M_{s,\eta'}$ to be positive for any cell s and haplotype blocks η and η', especially if the magnitude of $M_{s,\eta}$ and $M_{s,\eta'}$ are large (indicating strong evidence for imbalance). Note that swapping the phasing of haplotype block η simply multiplies $M_{s,\eta}$ by -1 for all cells s. Define p_η as our final phasing, which is either 1 or -1 for any haplotype block η. We want to maximize $p_\eta M_{s,\eta} \cdot p_{\eta'} M_{s,\eta'}$ for all cells s and for all haplotype blocks η and η'. Theoretically, if there were zero noise then $M_{s,\eta}$ should simply be proportional to the actual copy number imbalance in cell s multiplied by the number of counts in the haplotype block η and perhaps multiplied by -1 due to incorrect phasing. Let $\mathbf{M} = [M_{s,\eta}]$ be the matrix with entries $M_{s,\eta}$ across all cells s and haplotype blocks η on some chromosome. We can remove much of the noise by replacing $\mathbf{M}$ with a rank 1 matrix approximation produced by a singular value decomposition. That is, we have $\mathbf{M} \approx XY^\top$ such that $M_{s,\eta} \approx X_s \cdot Y_\eta$ where X_s represents a scaled version of the actual imbalance in cell s and Y_η represents a multiplicative factor which contains information on the phasing and total count of the haplotype block η. Then, we are simply maximizing $p_\eta X_s Y_\eta \cdot p_{\eta'} X_s Y_{\eta'} = X_s^2 (p_\eta Y_\eta \cdot p_{\eta'} Y_{\eta'})$. However, this is very easily accomplished by setting $p_\eta = \text{sign}(Y_\eta)$ for all haplotype blocks η, so that $p_\eta Y_\eta \cdot p_{\eta'} Y_{\eta'}$ is always positive.

Adjustments to phasing algorithm to improve results on highly variable chromosomes: The approach described above far works perfectly if one assumes that the copy number mostly remains constant for many cells in each chromosome, i.e., most CNAs only affect entire chromosomes. However, this is not a realistic assumption as many tumors have chromosome-arm CNA events as well as smaller, focal CNAs. Fig. S35, shows an example of a chromosome which would be phased incorrectly by the simplified phasing algorithm but correctly once adjustments are taken into account. In this hypothetical example, the first 50 cells have the A allele duplicated across all of chromosome 1 and 2000 remaining cells have a duplication of the A allele on the first fourth of chromosome 1, and a duplication of the B allele on the last fourth of chromosome 1. Our simplified phasing algorithm would primarily use the imbalance on all 2000 cells for phasing, and would incorrectly phase the 2000 cells to have a duplication of the A allele on both the first fourth and last fourth of the chromosome. Consequently, it is necessary to use a more sophisticated phasing algorithm that can keep track of the phasing of multiple groups of cells as well as the relative positioning of bins.

The simplest modification of this approach is to apply it to individual bins where one assumes the copy number remains approximately constant in each bin. However, utilizing information across an entire chromosome to eliminate noise and determine which subsets of cells are truly imbalanced and in what regions is very useful. For instance, in our example, the first set of 50 cells is useful for phasing across

the chromosome but the remaining 2000 cells are only useful for phasing within the first and forth quarter of the chromosome. Thus, it is ideal to first determine patterns of imbalances that occur in subsets of the chromosome and then utilize these patterns to phase haplotype blocks. To accomplish this, we first performed a singular value decomposition to approximate $\mathbf{M} = [M_{s,\eta}]$ with a higher, rank 10 matrix $[\tilde{M}_{s,\eta}]$. The purpose of this approach is to remove noise utilizing information across the entire chromosome to find patterns of imbalances in subsets of cells while allowing for differences to exist in different subsets of the chromosome.

Intuitively, the different singular vectors represent different patterns of imbalance in different subsets of cells. Specifically, in the example of Fig. S35, one singular vector could represent the imbalance in the first 50 cells and another singular component can represent the imbalance in the remaining 2000 cells. Despite, the two sets of cells being useful for phasing different parts of the chromosome they can be found simultaneously by low rank matrix approximation. Then, using non-overlapping groups of 100 consecutive haplotype blocks, we approximated $\tilde{\mathbf{M}}$ with a rank 1 matrix for each group in order to phase each group of 100 haplotype blocks as previously described. In our example, the imbalance in the first 50 cells is used for phasing in all groups of haplotype blocks, whereas the remaining 2000 cells only help to phase groups within the first and last quarter of the chromosome. After phasing within each group, we phase the groups together. Specifically, we updated the phasing of these groups of haplotype blocks by setting their phase to best match an exponential moving average of previous sets of 100 haplotype blocks with a smoothing factor of 0.9. Intuitively, we select the phasing of each group of haplotype blocks to have similar imbalances as the previous groups of haplotype blocks. In our example, the first 50 cells allow us to phase the first quarter of the chromosome with the next half of the chromosome and then to phase this next half with the last quarter of the chromosome. Mathematically, we have the following. Let $M^{\text{set}}_{s,\eta}$ indicate the average value of $M_{s,\eta'}$ for η' values in the ηth set of 100 haplotype blocks. Assume $M^{\text{set}}_{s,\eta}$ has already been phased for $\eta \leq K_{\text{phase}}$. Let $M^{\text{avg}}_{s,(\eta-1)}$ be the exponential moving average of $M^{\text{set}}_{s,\eta'}$ for $\eta' < \eta$. Then the moving average $M^{\text{avg}}_{s,\eta}$ is defined as $M^{\text{avg}}_{s,\eta} = \frac{9}{10} M^{\text{avg}}_{s,(\eta-1)} + \frac{1}{10} M^{\text{set}}_{s,\eta}$. Additionally, $M^{\text{set}}_{s,(\eta+1)}$ is phased by either multiplying by 1 or -1 depending on which phasing maximizes $\sum_{s=1}^{N} M^{\text{set}}_{s,(\eta+1)} M^{\text{avg}}_{s,\eta}$.

Once all of the phasing is completed, we determined the vector p_η such that p_η is either -1 or 1 for each haplotype block η depending on the phasing. Using this we defined the phased haplotype block counts as

$$C^{(1)}_{s,\eta} = \begin{cases} C^{A}_{s,\eta}, & \text{if } p_\eta = 1, \\ C^{B}_{s,\eta}, & \text{if } p_\eta = -1, \end{cases} \tag{3}$$

and

$$C_{s,\eta}^{(2)} = \begin{cases} C_{s,\eta}^{B}, & \text{if } p_\eta = 1, \\ C_{s,\eta}^{A}, & \text{if } p_\eta = -1. \end{cases} \tag{4}$$

Finally, we can define the haplotype-specific count vectors $A_s^{(1)}, A_s^{(2)} \in \mathbb{N}^K$. Specifically, $A_{s,i}^{(1)}$ is defined as the sum of $C_{s,\eta}^{(1)}$ for all haplotype blocks η in the bin i. Similarly, $A_{s,i}^{(2)}$ is defined as the sum of $C_{s,\eta}^{(2)}$ for all haplotype blocks k in the bin i.

Once $A_s = (A_s^{(1)}, A_s^{(2)})$ has been determined, we must correct for one additional issue. Specifically, invalid haplotype block phasing can occur when all cells have a complete loss of heterozygosity. Specifically, if all cells have a loss of heterozygosity for some bin (and no normal cells are provided) then haplotype blocks cannot be determined, and there will be near-zero counts for both haplotypes. To correct this, if some bin has less than one haplotype-specific count per cell (across all cells) then a pseudo-count of five is added to the first haplotype. Thus, regions with a complete loss of heterozygosity across all cells have a BAF near zero, rather than having a random BAF determined by noise and incorrect reads.

A.1.3 Estimating segments and noise levels

Given read depths $\tilde{\mathbf{R}} \in \mathbb{R}^{N \times K}$ and haplotype-specific counts $\mathbf{A}^{(1)}, \mathbf{A}^{(2)} \in \mathbb{N}^{N \times K}$ for N cells and K fixed-size bins, we now describe how we segmented the K bins into L segments and obtained read counts and corresponding variances $\mathbf{R}, \mathbf{\Sigma}^R \in \mathbb{R}^{N \times L}$ and BAFs and corresponding variances $\mathbf{B}, \mathbf{\Sigma}^B \in \mathbb{R}^{N \times L}$ for N cells and L segments. Our segmentation algorithm relies on estimates of the mean and variance of the read depth and BAF of consecutive bins to identify breakpoints. Therefore, before describing the segmentation algorithm, we will first discuss how to estimate the mean and variance of the read depth and BAF of a set Λ of consecutive fixed-size bins.

Estimating noise in segments: We are given a set Λ of fixed-size bins i for a cell, each of which has a read depth value $\tilde{R}_i$ and a number $A_i^{(1)}, A_i^{(2)}$ of counts of each haplotype. Additionally, the read depth vector is scaled to have an average value of 1 for mathematical convenience. We then estimate an average read depth and BAF for the set Λ of bins as well as a noise level in these estimates. The noise level refers to an estimated standard deviation of these average values. Additionally, this noise level can be squared to give estimated variances.

Calculating the average is trivial for the read depth (taking the mean value across bins) and finding the average BAF can be done by first summing haplotype counts across bins and then calculating the BAF from

this sum. More specifically, the average read depth is as below

$$R(\Lambda) = \frac{1}{|\Lambda|} \sum_{i \in \Lambda} \tilde{R}_i. \tag{5}$$

Additionally, the average BAF is given below

$$B(\Lambda) = \frac{\sum_{i \in \Lambda} A_i^{(2)}}{\sum_{j \in \Lambda} (A_j^{(1)} + A_j^{(2)})}. \tag{6}$$

For calculating the noise in the read depth, we first start with a simple statistical estimate of the noise in the set Λ of bins. This simple estimate starts with the assumption of independent noise across the bins in Λ. Define R_i^{diff} as $\tilde{R}_i$ minus the mean value of $\tilde{R}$ in the segment.

$$R_i^{\text{diff}} = \tilde{R}_i - \frac{1}{|\Lambda|} \sum_{j \in \Lambda} \tilde{R}_j. \tag{7}$$

The simplest way of calculating the read depth noise is by utilizing the standard error of the Λ measurements given below

$$\sqrt{\sum_{i \in \Lambda} \frac{1}{|\Lambda| - 1} (R_i^{\text{diff}})^2}. \tag{8}$$

In practice, there might be locations-specific noise for bins in Λ even if they have identical copy numbers and even after GC-bias correction. Therefore, we modify the simple estimate with a heuristic in order to achieve better performance in the case where the noise across bins is correlated. Specifically, our heuristic is approximately proportional to the standard error in the case that the noise is uncorrelated across bins, but increases substantially when the noise is highly correlated across bins. To accomplish this, we use the fact that the Fourier transform of independent noise is uniform across the frequency spectrum, whereas the Fourier transform of spatially localized noise (with high auto-correlation) is disproportionately low frequency. We define $F = [F_1, \dots, F_{|\Lambda|}]^\top$ as the Fourier transform of R^{diff} across the bins $i \in \Lambda$. The Fourier transform preserves the sum of squares such that the squared standard error is also calculated below

$$\sum_{i=1}^{|\Lambda|} \frac{1}{|\Lambda| - 1} F_i^2. \tag{9}$$

If the noise was uncorrelated across bins, we would expect an equal amplitude across the different frequencies. However, a higher amplitude of low frequency values would indicate auto-correlation in the noise values. Lower frequency values in the noise result in a higher error in the estimated average read depth. As a simple example, imagine the first $|\Lambda|/2$ bins all have one value, and the second $|\Lambda|/2$ bins all have some other value. Imagine this is due to the error in the read depth coming from some unknown bias which affects the first $|\Lambda|/2$ bins differently than the second $|\Lambda|/2$ bins. Then, the error in our estimate of the average

read depth would be equivalent to the scenario in which we only had two bins that we are averaging, since we only have two independent samples of the noise. Consequently, the squared error in our average read depth measurement is proportional to the inverse frequency of the noise. Specifically, we sum the square of each component in the Fourier transform divided by $|\Lambda|$ times the frequency of that component to get the square of the error. If the frequency were equal to $1/|\Lambda|$, this would give the standard error, however, the error increases as the frequency of the noise decreases. Define Λ_i as $|\Lambda|$ multiplied by the frequency of the ith component of the Fourier transform. Note that there is no zero-frequency component since R^{diff} has an average value of 0 by definition. Thus Λ_i is at most $|\Lambda|$. Then our heuristic estimate of the variance is the below slight modification to equation (9)

$$\Sigma^R(\Lambda) = \sum_{i=1}^{|\Lambda|} \frac{1}{\Lambda_i - 1} F_i^2. \tag{10}$$

The simplest way of estimating the noise in the average BAF is to only consider the total SNP counts across all bins. Each count can be treated as its own independent random variable with a value of either 0 for the A allele or 1 the B allele. Let C_a be the number of counts of the A allele and C_b be the number of counts of the B allele. Then, the BAF is

$$B_{\text{mean}} = \frac{C_b}{C_a + C_b} \tag{11}$$

and the variance in the BAF is

$$\frac{(C_b(B_{\text{mean}} - 1)^2 + C_a B_{\text{mean}}^2)}{(C_a + C_b)^2}. \tag{12}$$

To avoid zero noise in the case where the BAF is either 0 or 1, we add one pseudocount to C_a and C_b. This estimate is generally accurate, however, it assumes all counts are sampled from the same distribution across the Λ bins. This assumption may be broken due to incorrect phasing of haplotype blocks, very small focal CNAs within a segment or other errors in determining the counts of individual SNPs. To correct for this, we also note that the average BAF is equivalently calculated as the weighted average of the BAF of all Λ bins, weighted by the total SNP count in each bin. Let $\tilde{B}_i$ be the BAF of each of the bins $i \in \Lambda$ as below

$$\tilde{B}_i = \frac{A_i^{(2)}}{(A_i^{(1)} + A_i^{(2)})} \tag{13}$$

and let $C_i = A_i^{(1)} + A_i^{(2)}$ be the total count for both alleles in each bin i. The variance in the average BAF is then approximately

$$\frac{\sum_{i \in \Lambda} (\tilde{B}_i - B_{\text{mean}})^2 C_i^2}{(\sum_{j \in \Lambda} C_j)^2} \tag{14}$$

assuming the counts are spread across a large number of bins. The unbiased estimator has a slightly different denominator causing the error to go to infinity rather than 0 if there is a single bin. However, this estimate is only needed for a corrective term to add to the noise in the case where different bins appear to have SNP counts drawn from different distributions. Consequently, we achieve an effective estimate of the noise by using

$$\Sigma^B(\Lambda) = \frac{C_b(B_{\text{mean}} - 1)^2 + C_a B_{\text{mean}}^2}{(C_a + C_b)^2} + \frac{\sum_{i \in \Lambda}(\tilde{B}_i - B_{\text{mean}})^2 C_i^2}{(\sum_{j \in \Lambda} C_j)^2}. \tag{15}$$

As an example, in the case where only one bin has a non-zero count, the second term disappears and we simply estimate the noise from the allele counts in that bin. However, in the case where one bin has allele counts of $(100, 0)$ and another bin has allele counts of $(0, 100)$ the second term prevents us from falsely assuming we have a very low noise BAF of 0.5 with variance $1/800$, resulting in an adjusted variance of $1/800 + 1/8$.

Estimating segments: Our system starts with K bins of size ℓ_{bin} (set to $\ell_{\text{bin}} = 100$kb), and then merges these together into L segments where it is believed each cell has a constant copy number within each of these segments. To determine the boundary of these segments, one must find which positions in the genome have substantial evidence for a breakpoint in the copy number for some cells. Specifically, the evidence for a breakpoint is determined within each cell, and then this evidence is summed across cells. To determine the evidence for a breakpoint within a cell, we must determine the evidence that the last Γ bins prior to the breakpoint have a different read depth or BAF than the next Γ bins after the breakpoint. Larger values of Γ allow evidence to be accumulated across more bins, yet smaller values of Γ allow for the detection of smaller copy number aberrations. Consequently, we utilized $\Gamma \in \{10, 20, 40\}$. We determine a breakpoint to exist if there is sufficient evidence for any of these Γ values individually and do not sum evidence across multiple gamma values. Using these Γ values allows for the detection of small 1Mb copy number aberrations, while also allowing for evidence to be accumulated across 4Mb regions when detecting the boundary of larger copy number aberrations that may occur in only a small subset of cells.

Let $\Lambda_{\text{before}}, \Lambda_{\text{after}}$ be the set of Γ bins before and after some possible breakpoint position, respectively. Then the evidence for a breakpoint at that position in that cell (from the read depth and utilizing that value of Γ) is

$$\frac{|R(\Lambda_{\text{after}}) - R(\Lambda_{\text{before}})|}{\sqrt{\Sigma^R(\Lambda_{\text{before}}) + \Sigma^R(\Lambda_{\text{after}})}}. \tag{16}$$

The analogous computation is true for BAF:

$$\frac{|B(\Lambda_{\text{after}}) - B(\Lambda_{\text{before}})|}{\sqrt{\Sigma^B(\Lambda_{\text{before}}) + \Sigma^B(\Lambda_{\text{after}})}}. \tag{17}$$

This computation is done for all possible breakpoints, for all cells, for each of the three values of Γ, and for both the read depth and BAF. The evidence is summed across cells, giving 6 values for the evidence of each possible breakpoint. Our segmentation algorithm works by iteratively picking breakpoints with the most evidence but not allowing for two breakpoints to be close to each other and thus relying on the same evidence. For instance, if there is a large amount of evidence for a breakpoint at position x, then there will likely also be a large amount of evidence at position $x+1$, since the set of bins before and after the breakpoint are very similar for position x and position $x+1$. Thus, if one breakpoint uses Γ_1 bins, and another uses Γ_2 bins, then they must at least be a distance of $\min(\Gamma_1, \Gamma_2)$ bins apart. With this restriction in mind, the eligible breakpoints with the most evidence are iteratively added until no breakpoint has an evidence value of at least 3 times the number of cells. Note that this cutoff must be proportional to the number of cells since breakpoints will receive an evidence value near the number of cells just due to random chance. To avoid bins larger than 20Mb, this process is then applied again with the value of $\Gamma = 200$, iteratively adding breakpoints until there are no eligible breakpoints left (and thus all breakpoints are within 20Mb of some other breakpoint).

Once the set of segments is determined we set $R_{s,i}, B_{s,i}, \Sigma^R_{s,i}$ and $\Sigma^B_{s,i}$ to be the values of $R(\Lambda(i)), B(\Lambda(i)), \Sigma^R(\Lambda(i))$, and $\Sigma^B(\Lambda(i))$ for cell s where $\Lambda(i)$ is the set of fixed-size bins comprising segment i.

A.2 CNNaive

The goal of the CNNaive pipeline is to produce the inputs required by CNRein. CNNaive takes as input measurements $\mathbf{R}, \mathbf{B}, \mathbf{\Sigma}^R, \mathbf{\Sigma}^B \in \mathbb{R}^{N \times L}$ obtained using the previously described data processing steps and produces preliminary copy numbers $\tilde{\mathbf{P}}^{(1)}, \tilde{\mathbf{P}}^{(2)} \in \mathbb{N}^{N \times L}$ for N cells and L segments. These copy numbers help guide CNRein's reinforcement learning search. In this section, we describe the process of generating these preliminary copy number profiles.

A.2.1 Defining measurement probabilities

In this section, we define the probability $\Pr(R_s \mid \Sigma^R_s, P)$ of read depth values R_s and the probability $\Pr(B_s \mid \Sigma^B_s, P)$ of BAF values B_s given a copy number profile P and estimates of the variances Σ^R_s, Σ^B_s of these values of a cell s. This is utilized in producing copy number profiles for both CNNaive and CNRein. Given the copy number profile $P = (P^{(1)}, P^{(2)})$, we can predict the read counts to be proportional to $P^{(1)} + P^{(2)}$. Thus, if we predict the correct copy number profile, we predict $c \cdot (P^{(1)} + P^{(2)})$ to match R_s on expectation for some scaling constant c. Given the central limit theorem, it is reasonable to assume

measurement values come from a Gaussian distribution. Then, the probability $\Pr(R_s \mid \Sigma^R_s, P)$ of observing R_s given P and Σ^R_s is the following product of Gaussians with variances $\Sigma^R_{s,i}$.

$$\Pr(R_s \mid \Sigma^R_s, P) = \prod_{i=1}^{L} \frac{1}{\sqrt{\Sigma^R_{s,i} 2\pi}} \exp\left(\frac{-(R_{s,i} - c \cdot (P_i^{(1)} + P_i^{(2)}))^2}{2\Sigma^R_{s,i}} \right). \tag{18}$$

Note that this expression is minimized by minimizing the below total squared error.

$$\sum_{i=1}^{L} \frac{(R_{s,i} - c \cdot (P_i^{(1)} + P_i^{(2)})^2}{\Sigma^R_{s,i}}. \tag{19}$$

For evaluating a copy number profile P as used by CNRein, we set c to $(\sum_{i=1}^{L} R_{s,j} \Sigma^R_{s,j}) / (\sum_{i=1}^{L} (P_i^{(1)} + P_i^{(2)}) \Sigma^R_{s,i})$ in order to minimize this error and maximize the probability. For CNNaive, we set $c = \gamma^{-1}$ where γ is the cell-specific scaling factor discussed in Section A.2.2.

The expected value of the B-allele frequency in bin i given the copy number profile P is $P_i^{(2)} / (P_i^{(1)} + P_i^{(2)})$. Similarly as for the read count, for the B-allele frequency, we have the below equation.

$$\Pr(B_s \mid \Sigma^B_{s,i}, P) = \prod_{i=1}^{L} \frac{1}{\sqrt{\Sigma^B_{s,i} 2\pi}} \exp\left(\frac{-(B_{s,i} - \frac{P_i^{(2)}}{P_i^{(1)} + P_i^{(2)}})^2}{2\Sigma^B_{s,i}} \right). \tag{20}$$

Again, maximizing this probability is equivalent to minimizing the below total squared error

$$\sum_{i=1}^{L} \frac{(B_{s,i} - \frac{P_i^{(2)}}{P_i^{(1)} + P_i^{(2)}})^2}{\Sigma^B_{s,i}}. \tag{21}$$

A.2.2 Estimating cell-specific scaling factor

Given a ground truth profile P, the ground truth cell-specific scaling factor γ_{true} is defined as the average total copy number $(1/L) \sum_{i=1}^{L} (P_i^{(1)} + P_i^{(2)})$. In this section, we utilize variance aware segmentation to estimate the cell-specific scaling factor given an allele counts vector $A = [A^{(1)}, A^{(2)}]^\top \in \mathbb{N}^{2 \times K}$ and a read depth vector $\tilde{R} \in \mathbb{R}^K$. These vectors are modified to use 1Mb bins formed out of merging our original K bins of fixed size 100kb. These larger bins allow for a more efficient exhaustive search of all possible segments within each chromosome. Additionally, any loss of precision due to using large bins is not relevant since very small copy number aberrations are not relevant for determining the cell-specific scaling factor.

Determining low variance segments: Before describing our approach, we give a brief conceptual motivation. If one had perfect noise-free read depth and B-allele frequency measurements, then determining the cell-specific scaling factor would be trivial. One could simply select the smallest cell-specific scaling factor

such that integer copy numbers can result in the observed read depth and allelic imbalance values. Similarly, if one had a list of highly accurate pairs of read depth and B-allele frequency measurements, one could easily find the cell-specific scaling factor that best fits the measurements. In reality, read depth and B-allele frequency measurements are very noisy. As such, our method works by first finding low-noise segments that provide highly accurate measurements of the B-allele frequency and the mean read depth within those segments. Then we evaluate if those high-quality read depth and BAF values would be possible for a given value of γ. Finally, we determine which value of γ best matches these observed measurements.

We will define $V(\tilde{R}, A, i, j)$ as a function that inputs the read depth vector $\tilde{R}$, the allele-specific count vector A, some starting bin i and some ending bin j, and outputs a measurement of how accurately we know the mean $\tilde{R}$ and BAF value within that segment. If $V(\tilde{R}, A, i, j)$ is high, then this implies an accurate measurement of a mean read depth and B-allele frequency value pair that must be possible to observe for a given cell-specific scaling factor. The goal is to split the $\tilde{R}$ and A vectors into segments that maximize this value. We define $\mu_R(\tilde{R}, i, j)$ as the mean value of $\tilde{R}$ on the interval from i to j, i.e.,

$$\mu_R(\tilde{R}, i, j) = \frac{1}{j - i + 1} \sum_{i'=i}^{j} \tilde{R}_{i'}. \tag{22}$$

We define $\sigma_R(\tilde{R}, i, j)$ as the standard error of this mean value, i.e.,

$$\sigma_R(\tilde{R}, i, j)^2 = \frac{1}{(j - i)^2} \sum_{i'=i}^{j} (\tilde{R}_{i'} - \mu_R(\tilde{R}, i, j))^2. \tag{23}$$

We define $\mu_B(A, i, j)$ as the B-allele frequency of the total allele-specific counts in A on the interval i to j, i.e.,

$$\mu_B(A, i, j) = \frac{\sum_{i'=i}^{j} A_{i'}^{(2)}}{\sum_{j'=i}^{j} (A_{j'}^{(1)} + A_{j'}^{(2)})}. \tag{24}$$

Finally, we define $\sigma_B(A, i, j)$ as the noise estimate for $\mu_B(A, i, j)$ as described in Section A.1.3.

Let $L_{\min}$ be the minimum length segment (in terms of $\ell_{\text{bin}} = 100\text{kb}$ bins) we consider acceptable for giving an estimated error in the average read depth and B-allele frequency values within that segment. We use $L_{\min} = 5$ as a default setting. Taking the inverse of our error estimate gives a measurement of precision. Thus, adding together an inverse of these error values for the read depth and B-allele frequency gives

$$V(\tilde{R}, A, i, j) = \frac{1}{\sigma_R(\tilde{R}, i, j)} + \frac{1}{\sigma_B(A, i, j)}. \tag{25}$$

Now that we have a measurement of the accuracy of read depth and BAF values provided by some segments, we can move on to defining the problem of determining an optimal variance-aware segmentation. Define A_S as the set of all possible lists of non-overlapping segments of length at least $L_{\min}$

on L bins such that each segment is contained within a chromosome. More precisely, define $A_S = \{\{(i^1, j^1), \ldots, (i^{K_{\text{seg}}}, j^{K_{\text{seg}}})\} \mid 1 \leq i^\kappa, i^\kappa + L_{\min} - 1 \leq j^\kappa < i^{\kappa+1}, i^{\kappa+1} + L_{\min} - 1 \leq j^{\kappa+1} \leq L$, for all $\kappa \in \{1, \ldots, K_{\text{seg}} - 1\}$, and i^κ and j^κ are on the same chromosome for all $\kappa \in \{1, \ldots, K_{\text{seg}}\}\}$. We then have the following problem of variance-aware segmentation.

Problem 1 (LOW VARIANCE SEGMENTS). *Given a read depth vector $\tilde{R}$ and a allelic imbalance vector B, find the list of segments $\{(i^1, j^1), \ldots, (i^{K_{seg}}, j^{K_{seg}})\} \in A_S$ that maximize $\sum_{\kappa=1}^{K_{seg}} V(\tilde{R}, B, i^\kappa, j^\kappa)$.*

Solving Problem 1 with an exhaustive search is computationally infeasible. Therefore, we use a greedy approach as an approximate solution. Specifically, within each chromosome, we first find segment i to j of length at least $L_{\min}$ that maximize $V(\tilde{R}, B, i, j)$. Then, iteratively, we find the segment i to j of length at least $L_{\min}$ that maximizes $V(\tilde{R}, B, i, j)$ without overlapping with any existing segment. The process stops when it is not possible to add any additional intervals of length at least $L_{\min}$ without having overlapping segments. The values of $\sigma_R(\tilde{R}, i, j)$ and $\sigma_B(A, i, j)$ can be calculated efficiently for each segment once the appropriate cumulative sums of input vectors and their squares have been stored. For instance, once the cumulative sum across the genome of $\tilde{R}$ and $\tilde{R}^2$ have been stored, $\sigma_R(\tilde{R}, i, j)$ can be calculated in constant time independent of the length of the segment.

Determining cell-specific scaling factor from low variance segments: After finding an approximate solution to Problem 1, we have a list of segments $\{(i^1, j^1), \ldots, (i^{K_{\text{seg}}}, j^{K_{\text{seg}}})\}$. For each of these segments, we have an estimated mean read depth and allelic imbalance as well as an estimated error in that estimate. Define $\overline{R}^\kappa = \mu_R(\tilde{R}, i^\kappa, j^\kappa)$, $\overline{B}^\kappa = \mu_B(A, i^\kappa, j^\kappa)$, for $\kappa \in \{1, \ldots, K_{\text{seg}}\}$. Also define $\overline{B}^\kappa_{\text{error}} = \sigma_B(A, i^\kappa, j^\kappa)$, and $\overline{R}^\kappa_{\text{error}} = \sigma_R(\tilde{R}, i^\kappa, j^\kappa)$.

Given a cell-specific scaling factor γ, possible values for the read depth and allelic imbalance are of the form $(N_1 + N_2)/\gamma$ and $\min(N_1, N_2)/(N_1 + N_2)$, respectively, where N_1 and N_2 are both non-negative integers representing haplotype copy numbers. Define $f_R(N_1, N_2, \gamma) = (N_1 + N_2)/\gamma$, and $f_B(N_1, N_2) = N_2/(N_1 + N_2)$. We define the equation for the minimum error of a read depth and allelic imbalance measurement given a cell-specific scaling factor as

$$M_{\text{error}}(\gamma, \overline{R}^\kappa, \overline{B}^\kappa) = \min_{N_1, N_2} \left(\frac{f_R(N_1, N_2, \gamma) - \overline{R}^\kappa}{\overline{R}^\kappa_{\text{error}}} \right)^2 + \left(\frac{f_B(N_1, N_2) - \overline{B}^\kappa}{\overline{B}^\kappa_{\text{error}}} \right)^2. \quad (26)$$

We note that the number of possible $f_R(N_1, N_2, \gamma)$ and $f_B(N_1, N_2)$ values while keeping $f_R(N_1, N_2, \gamma)$ within some range (for instance 0 to 1) is proportional to γ. Therefore, for random read depth and allelic imbalance values, one would expect the squared error $M_{\text{error}}(\gamma, \overline{R}^\kappa, \overline{B}^\kappa)$ to scale proportional to γ^{-2}. Consequently, our goal is to minimize $M_{\text{error}}(\gamma, \overline{R}^\kappa, \overline{B}^k)\gamma^2$ rather than simply minimizing $M_{\text{error}}(\gamma, \overline{R}^\kappa, \overline{B}^\kappa)$.

As further intuition, note that $M_{\text{error}}(\gamma, \overline{R}^{\kappa}, \overline{B}^{\kappa})$ can be made arbitrarily small for any $\overline{R}^{\kappa}$, $\overline{B}^{\kappa}$ within the range of possible values by setting γ arbitrarily large. Putting this all together, our goal is to select γ to minimize the total error

$$\gamma^* = \text{argmin}_{\gamma} \sum_{\kappa=1}^{K_{\text{seg}}} M_{\text{error}}(\gamma, \overline{R}^{\kappa}, \overline{B}^{\kappa})\gamma^2. \tag{27}$$

Additionally, we apply the constraint $\gamma \geq 1$ to avoid the solution of setting γ extremely low while predicting copy numbers of all zeros for all bins. This single variable minimization is accomplished in our code by a simple 1-dimensional grid search (in our implementation the grid search starts at 1 and ends at 10 with exponential increments of $\exp(0.02)$ followed by a secondary local search around the optimal solution with increments of $\exp(0.002)$). As a slight caveat, if there exists an additional value of γ that approximately minimizes $M_{\text{error}}(\gamma, \overline{R}^{\kappa}, \overline{B}^{\kappa})\gamma^2$ (off by at most 10), and is not within a ratio of 20% of γ^*, then this value of γ is also reported. For most cells, this does not occur, but for a few cells there exists multiple similar quality cell-specific scaling factors.

A.2.3 The predicted profiles of CNNaive

In Section A.2.2 we gave a method for predicting cell-specific scaling factors. These scaling factors can then be utilized to give a basic prior estimate of the copy number profiles $\tilde{\mathbf{P}}^{(1)}, \tilde{\mathbf{P}}^{(2)} \in \mathbb{N}^{N \times L}$ for N cells and L segments. As derived in Section A.2.1, the log probability $\log \Pr(R_s \mid \Sigma_s^R, P)$ of a read depth vector R_s given variances Σ_s^R the copy number profile P is proportional to

$$-\sum_{i=1}^{L} \frac{(R_{s,i} - c \cdot (P_i^{(1)} + P_i^{(2)}))^2}{\Sigma_{s,i}^R} \tag{28}$$

where c is a constant. In the case that we know the cell-specific scaling factor γ, this becomes the below expression.

$$-\sum_{i=1}^{L} \frac{(R_{s,i} - \gamma^{-1}(P_i^{(1)} + P_i^{(2)}))^2}{\Sigma_{s,i}^R} \tag{29}$$

Similarly, the log probability of the BAF vector B_s given the copy number profile P is proportional to

$$-\sum_{i=1}^{L} \frac{\left(B_{s,i} - \frac{P_i^{(2)}}{P_i^{(1)}+P_i^{(2)}}\right)^2}{\Sigma_{s,i}^B}. \tag{30}$$

Consequently, the optimal copy number profile $\tilde{P} = (\tilde{P}^{(1)}, \tilde{P}^{(2)})$ for maximizing $\Pr(R_s, B_s \mid \Sigma_s^R, \Sigma_s^B, P)$ minimizes the below expression.

$$\sum_{i=1}^{L} \left[\frac{(R_{s,i} - \gamma^{-1}(P_i^{(1)} + P_i^{(2)}))^2}{\Sigma_{s,i}^R} + \frac{\left(B_{s,i} - \frac{P_i^{(2)}}{P_i^{(1)}+P_i^{(2)}}\right)^2}{\Sigma_{s,i}^B} \right]. \tag{31}$$

Fortunately, the error term for each bin i only depends on the copy numbers for that bin $P_i^{(1)}$ and $P_i^{(2)}$ so we simply select these values to minimize the error for each bin. Applying this to the BAF vector and read depth vector for each cell (given the cell-specific scaling factor for this cell) gives CNNaive's predicted copy number profiles $\tilde{\mathbf{P}}^{(1)}, \tilde{\mathbf{P}}^{(2)} \in \mathbb{N}^{N \times L}$ for each of the N cells and L segments.

A.3 CNRein

A.3.1 Policy learning equation derivation

In the main text, we state the equation

$$\frac{d}{d\theta} \log(\Pr(\mathbf{R}, \mathbf{B} \mid \mathbf{\Sigma}^R, \mathbf{\Sigma}^B, \theta)) = \mathbb{E}_{G \sim \Pr(\cdot \mid \theta)} \left[\sum_{s=1}^{N} \Pr(R_s, B_s \mid \theta)^{-1} \Pr(R_s, B_s \mid G) \frac{d}{d\theta} \log(\Pr(G \mid \theta)) \right]. \tag{32}$$

As stated in the main text, we use the shorthands $\Pr(R_s, B_s \mid G) = \Pr(R_s, B_s \mid \Sigma_s^R, \Sigma_s^B, g(G))$ and $\Pr(R_s, B_s \mid \theta) = \Pr(R_s, B_s \mid \Sigma_s^R, \Sigma_s^B, \theta)$. Below is the derivation of this equation.

$$\frac{d}{d\theta}\log(\Pr(\mathbf{R},\mathbf{B}\mid \mathbf{\Sigma}^R,\mathbf{\Sigma}^B,\theta)) \tag{33}$$

$$= \frac{d}{d\theta}\sum_{s=1}^{N}\log(\Pr(R_s,B_s\mid\theta)) \tag{34}$$

$$= \sum_{s=1}^{N}\frac{d}{d\theta}\log(\Pr(R_s,B_s\mid\theta)) \tag{35}$$

$$= \sum_{s=1}^{N}\Pr(R_s,B_s\mid\theta)^{-1}\frac{d}{d\theta}\Pr(R_s,B_s\mid\theta) \tag{36}$$

$$= \sum_{s=1}^{N}\Pr(R_s,B_s\mid\theta)^{-1}\frac{d}{d\theta}\sum_{P\in\mathcal{S}}\Pr(P\mid\theta)\Pr(R_s,B_s\mid P) \tag{37}$$

$$= \sum_{s=1}^{N}\Pr(R_s,B_s\mid\theta)^{-1}\frac{d}{d\theta}\sum_{G\subseteq\mathcal{C}}\Pr(G\mid\theta)\Pr(R_s,B_s\mid G) \tag{38}$$

$$= \sum_{s=1}^{N}\Pr(R_s,B_s\mid\theta)^{-1}\sum_{G\subseteq\mathcal{C}}\Pr(R_s,B_s\mid G)\frac{d}{d\theta}\Pr(G\mid\theta) \tag{39}$$

$$= \sum_{s=1}^{N}\Pr(R_s,B_s\mid\theta)^{-1}\sum_{G\subseteq\mathcal{C}}\Pr(R_s,B_s\mid G)\Pr(G\mid\theta)\frac{d}{d\theta}\log(\Pr(G\mid\theta)) \tag{40}$$

$$= \sum_{G\subset A_C}\Pr(G\mid\theta)\sum_{s=1}^{N}\Pr(R_s,B_s\mid\theta)^{-1}\Pr(R_s,B_s\mid G)\frac{d}{d\theta}\log(\Pr(G\mid\theta)) \tag{41}$$

$$= \mathbb{E}_{G\sim\Pr(\cdot|\theta)}\left[\sum_{s=1}^{N}\Pr(R_s,B_s\mid\theta)^{-1}\Pr(R_s,B_s\mid G)\frac{d}{d\theta}\log(\Pr(G\mid\theta))\right]. \tag{42}$$

A.3.2 Probability estimation via sampling

In several steps of our method, we need to estimate probabilities using sampling. Specifically, we need to estimate $\Pr(R_s, B_s \mid \theta)$ for the reward function calculation, and we need to estimate $\Pr(P \mid \theta)$ for copy number profile prediction. Let $\mathcal{A}(P)$ be the set of generating sequences that efficiently generate P, as defined in Section A.3.6. Let $P_1, \ldots, P_M$ be the copy number profiles used to guide our search as described in Section A.3.3. Note $g(G) = P$ if $G \in \mathcal{A}(P)$. To estimate $\Pr(R_s, B_s \mid \theta)$, we use

$$\Pr(R_s,B_s\mid\theta) = \mathbb{E}_t\left[\mathbb{E}_{G\sim\Pr(\cdot|\mathcal{A}(P_t),\theta)}\left[\frac{\Pr(G\mid\theta)M}{\Pr(G\mid\mathcal{A}(P_t),\theta)}\Pr(R_s,B_s\mid G,\theta)\right]\right] \tag{43}$$

where t is sampled uniformly from $\{1,\ldots,M\}$. Note that this value changes during training and so we re-estimate it after each gradient update to the model's parameters θ. Define $f_{\text{same}}(P,P') = 1$ if $P = P'$

and 0 otherwise. To estimate $\Pr(P \mid \theta)$, we use

$$\Pr(P \mid \theta) = \mathbb{E}_t \left[\mathbb{E}_{G \sim \Pr(\cdot \mid \mathcal{A}(P_t), \theta)} \left[\frac{\Pr(G \mid \theta) M}{\Pr(G \mid \mathcal{A}(P_t), \theta)} f_{\text{same}}(P, g(G)) \right] \right]. \tag{44}$$

A.3.3 Guiding the optimization with initial copy number profiles

In order to guide the optimization, we start with a reasonable prior on generating sequences of CNA tuples. In this section, we describe a slightly simpler version of this prior, which is then improved on in Section A.3.4 and A.3.5. We start with a set $P_1, \dots, P_M$ of plausible copy number profiles. Specifically, we utilize the unique profiles predicted by the CNNaive, as well as a set of additional copy number profiles determined during training as described in Section A.3.5. As such, $M \geq N$. Then, we restrict our generating sequences to efficiently generate copy number profiles in $\{P_1, \dots, P_M\}$. We define $\mathcal{A}(P)$ as a set of generating sequences that efficiently generate P. Intuitively, generative sequences in $G \in \mathcal{A}(P)$ generate the profile P ($g(G) = P$) without needless additional steps such as adding CNA events only to remove those exact same CNA events (mathematical technicalities of this definition are provided in Section A.3.6). Note that $g(G) = P$ if $G \in \mathcal{A}(P)$. Define $\mathcal{A} = \cup_{t=1}^{M} \mathcal{A}(P_t)$. We define $\Pr(G \mid \mathcal{A}(P), \theta)$ as the probability of sampling G when restricting to $\mathcal{A}(P)$ which is defined precisely in A.3.7. To sample when restricted to $\mathcal{A}$, we first uniformly sample t from $\{1, \dots, M\}$ and then sample G from $\Pr(G \mid \mathcal{A}(P_t), \theta)$. Thus, $\Pr(G \mid \mathcal{A}, \theta) = (1/M) \sum_{t=1}^{M} \Pr(G \mid \mathcal{A}(P_t), \theta)$.

Define $B(G, \mathcal{A}) = 1$ if $G \in \mathcal{A}$ and 0 otherwise. For completeness, we repeat the definition of the reward function $r(G)$ given in the main text.

$$r(G) = \sum_{s=1}^{N} \Pr(R_s, B_s \mid \theta)^{-1} \Pr(R_s, B_s \mid G). \tag{45}$$

To restrict generating sequences to $\mathcal{A}$, we multiply $r(G)$ (defined in equation (45)) by $B(G, \mathcal{A})$ in the original objective function. Since we are modifying our sampling procedure, we must also modify our training to compensate for that.

$$\mathbb{E}_{G \sim \Pr(\cdot \mid \theta)} [r(G) B(G, \mathcal{A}) \log(\Pr(G \mid \theta))] \tag{46}$$

$$= \mathbb{E}_t \left[\mathbb{E}_{G \sim \Pr(\cdot \mid \mathcal{A}(P_t), \theta)} \left[\frac{\Pr(G \mid \theta) M}{\Pr(G \mid \mathcal{A}(P_t), \theta)} r(G) \log(\Pr(G \mid \theta)) \right] \right]. \tag{47}$$

Define $r'(G, t) = \Pr(G \mid \theta) M \Pr(G \mid \mathcal{A}(P_t), \theta)^{-1} r(G)$, as our new reward function for our new sampling procedure. For those familiar, note, the term $\Pr(G \mid \theta) M \Pr(G \mid \mathcal{A}(P_t), \theta)^{-1}$ is the adjustment used for importance sampling [6] (since $\Pr(G \mid \mathcal{A}(P_t), \theta)/M$ is the sampling probability). We now have the

objective function

$$\mathbb{E}_t \left[\mathbb{E}_{G \sim \Pr(\cdot | \mathcal{A}(P_t), \theta)} \left[r'(G, t) \log(\Pr(G \mid \theta)) \right] \right]. \tag{48}$$

After learning the model parameters θ, we utilize sampling to estimate which copy number profile P maximizes $\Pr(R_s, B_s \mid P) \Pr(P \mid \theta)$ for each cell s as described in Section A.3.2.

A.3.4 A modification to improve the removal of spurious CNAs

The prior copy number profile estimates provided by CNNaive are likely to contain fake CNAs that only appear to exist due to noise. Our method described thus far should correct for this so long as $P_1, \dots, P_M$ contains most of the correct copy number profiles in addition to some profiles with fake CNAs. However, in high noise data sets it is possible for most or all of the CNNaive estimates to contain fake CNAs. To correct for this, our method must delete fake CNAs. To accomplish this, we allow our method to predict copy number profiles $g([c^1, \dots, c^k])$ for any subsequence $[c^1, \dots, c^k]$ of $[c^1, \dots, c^k, \dots, c^{k+r}] \in \mathcal{A}$. Note, $g([c^1, \dots, c^k])$ is the copy number profile $g([c_1, \dots, c_k, \dots, c_{k+r}])$ but with the last r CNAs removed. Thus, more intuitively but less precisely, we allow our model to predict copy number profiles which are like some profile in $P_1, \dots, P_M$ but with some CNAs removed. The details of this modified sampling procedure are described in Section A.3.7. Additional modifications to the search space to allow for other copy number profiles is described in Section A.3.5.

A.3.5 Modifying the search space of copy number profiles

To guide our reinforcement learning, our model is restricted to generating copy number profiles in some set of given copy number profiles (in addition to modifications of those copy number profiles by removing CNAs). CNNaive provides this set of initial copy number profiles. However, for the sake of improving the search, we add additional copy number profiles to the set of profiles that are allowed to be generated. During any iteration, for each cell s, the copy number profile that maximizes $\Pr(P \mid \theta) \Pr(R_s, B_s \mid P)$ is calculated. We refer to this as the best-fit copy number profile for cell s in iteration t. For the next iteration, this copy number profile is included in the list of copy number profiles to be generated. Specifically, this changes the list of copy number profiles to be generated if this best-fit profile P was originally generated by removing CNAs from some copy number profiles originally in the list of profiles to be generated (described in Section A.3.4). Additionally, for each best-fit copy number profile, a new copy number profile is generated by randomly changing the copy numbers for one chromosome to the copy numbers of some other best-fit copy number profile. These copy number profiles are also added to the list of profiles to be generated

in the next iteration. This increases exploration during training. Additionally, another set of modified copy number profiles is added in order to avoid local minima in the cases of certain bins having very low haplotype-specific read counts. Specifically, the set of best fit profiles for each cell is copied, and then their haplotype-specific copy numbers are modified to better fit their haplotype-specific counts. To do so, for each bin and each copy number, the algorithm identifies all cells with that given copy number in that bin. The haplotype-specific read count of all of those cells in that bin is then calculated giving values C^A, C^B. Then, cell specific copy number (X^A, X^B) is modified to best fit C^A, C^B while retaining the same total copy number. Let $B^{\text{tweak}} = 0.9(X^B/(X^A + X^B)) + 0.05$ be the BAF implied by the copy numbers (X^A, X^B) with a slight adjustment to avoid 0 or 1 probabilities. The algorithm then selects haplotype specific copy numbers and maximizes the log probability $C^A \log(1 - B^{\text{tweak}}) + C^B \log(B^{\text{tweak}})$. For many bins, this procedure will likely not modify the copy number profile. However, in cases with extremely low haplotype-specific read counts, the profiles may be improved due to this procedure of pooling counts together across cells. Note that these modified profiles are simply added to the set of possible profiles during training and are not required to be used by CNRein.

A.3.6 Efficient generative sequences

We previously mentioned the concept of sets of generative sequences that efficiently generate a copy number profile. Intuitively, these are simply sequences of copy number aberrations that result in a copy number profile without needless additional events such as adding CNAs only to remove those exact added CNAs. However, the concept is defined with mathematical precision here. To do so, we first determine a set of binary criteria for a CNA event efficiently moving some existing profile towards some output profile. Specifically, we define B_E^{start}, B_E^{end}, and B_E^{error} to detect if a CNA matches some start position and end position in the desired copy number profile in addition to moving the existing profile closer to this profile. Define $B_E(P', P, c)$ as a function that inputs two copy number profiles P', P, and one CNA tuple c, and outputs either 0 or 1. We will define $B_E(P', P, c)$ to output 1 if c efficiently modifies P' towards P, and 0 otherwise. At first, we will ignore the existence of whole genome duplications, and then describe the modification to allow whole genome duplications.

Intuitively, define $B_E^{\text{start}}(P', P, c_{\text{hap}}, c_{\text{start}}) = 1$ if c_{start} is the start position of a region of constant copy

number in $P^{(c_{\text{hap}})}$ and 0 otherwise. Mathematically, we have

$$B_E^{\text{start}}(P', P, c_{\text{hap}}, c_{\text{start}}) = \begin{cases} 1, & \text{if } P^{(c_{\text{hap}})}_{c_{\text{start}}} \neq P^{(c_{\text{hap}})}_{c_{\text{start}}-1} \,, \\ 1, & c_{\text{start}} \text{ is the first bin of a chromosome,} \\ 0, & \text{otherwise.} \end{cases} \tag{49}$$

Similarly, we define $B_E^{\text{end}}(P', P, c_{\text{hap}}, c_{\text{end}}) = 1$ if c_{end} is the end of a region of constant copy number in $P^{c_{\text{hap}}}$ and 0 otherwise. Mathematically, we have

$$B_E^{\text{end}}(P', P, c_{\text{hap}}, c_{\text{end}}) = \begin{cases} 1, & \text{if } P^{(c_{\text{hap}})}_{c_{\text{end}}} \neq P^{(c_{\text{hap}})}_{c_{\text{end}}+1} \,, \\ 1, & c_{\text{end}} \text{ is the last bin of a chromosome,} \\ 0, & \text{otherwise.} \end{cases} \tag{50}$$

These two functions help ensure that our CNA tuple has a c_{start} at the start and c_{end} at the end for the CNA in P. Intuitively, let $B_E^{\text{error}}(P', P, c) = 1$ if the copy number aberration c results in P' being more similar to P. Define $\text{Dist}(P, P')$ as the L1 distance between any two copy number profiles P and P'. As defined in Main text: "Evolutionary model for computing profile probabilities", let $f_g(P, G)$ be a function that inputs a copy number profile P and a list G of CNA tuples and applies those CNAs to the profile. Mathematically, we have

$$B_E^{\text{error}}(P', P, c) = \begin{cases} 1, & \text{if } \text{Dist}(P, P') > \text{Dist}(P, f_g(P', c)) \,, \\ 0, & \text{otherwise.} \end{cases} \tag{51}$$

This function ensures that adding the CNA c to P' results in a copy number profile that is closer to P. Utilizing these we have the below equation

$$B_E(P', P, c) = B_E^{\text{start}}(P', P, c_{\text{hap}}, c_{\text{start}}) B_E^{\text{end}}(P', P, c_{\text{hap}}, c_{\text{end}}) B_E^{\text{error}}(P', P, c). \tag{52}$$

This equation then determines if the CNA c efficiently moves the profile P' towards the desired profile P. If the median total copy number in P is 4 or more then we ensure efficient generating sequences include a whole genome duplication (WGD). Specifically, if a whole genome duplication has not occurred yet, we replace $B_E(P', P, c)$ with $B_E(P', P/2, c)$, anticipating the duplication which will occur in P'. Additionally, we allow a whole genome duplication to occur once per cell in the generative process. Once the whole genome duplication has already occurred, then $B_E(P', P, c)$ is utilized as normal (since the doubling of P' has already occurred). Note that while we do not allow a WGD to occur multiple times in a cell's evolutionary history (as modeled by G), we do allow distinct cells to undergo distinct WGDs in their evolutionary

histories. Effectively, this supports the existence of potentially, multiple subclonal WGDs as well as a single truncal WGD.

Given our function $B_E(P', P, c)$, we have the following constructive definition of the set $\mathcal{A}^*(P)$ of generative sequences that efficiently generate P and its intermediates. Initially, let $\mathcal{A}^*(P)$ contain the empty set $\{\}$. Inductively, define $[c^1, \dots, c^k] \in \mathcal{A}^*(P)$ if $[c^1, \dots, c^{k-1}] \in \mathcal{A}^*(P)$ and $B_E(g([c^1, \dots, c^{k-1}]), P, c^k) = 1$. Finally, define the set $\mathcal{A}(P)$ of efficient generative sequences that generate exactly P as $\mathcal{A}(P) = \{G \in \mathcal{A}^*(P) \mid g(G) = P\}$.

A.3.7 Restricted sampling probabilities

In this section, we describe sampling restricted to $\mathcal{A}(P)$. Let $\mathcal{A}^*(P)$ be as defined in Section A.3.6. First, define $\mathcal{B}(P, [c^1, \dots, c^k]) = \{c^{k+1} \mid [c^1, \dots, c^{k+1}] \in \mathcal{A}^*(P)\}$. In other words $\mathcal{B}(P, [c^1, \dots, c^k])$ defines the set of subsequent CNA tuples that can occur after the application of $[c^1, \dots, c^k]$ to the normal profile $P_\emptyset$ in the process of efficiently generating P. This allows us to define the sampling probability of each new CNA tuple given existing CNA tuples as

$$\Pr(c \mid g(G), \mathcal{A}(P), \theta) = \frac{\Pr(c \mid g(G), \theta)}{\sum_{c' \in \mathcal{A}(P,G)} \Pr(c' \mid g(G), \theta)}. \tag{53}$$

We recursively define a probability for any generating sequence $[c^1, \dots, c^k] \in \mathcal{A}^*(P)$ as

$$\Pr([c^1, \dots, c^k] \mid \mathcal{A}(P), \theta) = \Pr(c^k \mid [c^1, \dots, c^{k-1}], \mathcal{A}(P), \theta) \Pr([c^1, \dots, c^{k-1}] \mid \mathcal{A}(P), \theta). \tag{54}$$

If $G = [c^1, \dots, c^k] \in \mathcal{A}(P)$, then $\Pr(G \mid \mathcal{A}(P), \theta)$ is the sampling probability of G (prior to the modification for removing fake CNAs).

In Section A.3.4 we state that the sampling procedure is modified to help remove fake CNAs. Specifically, we allow the model to predict any $[c^1, \dots, c^k]$ with $[c^1, \dots, c^k, \dots, c^{k+r}] \in \mathcal{A}$, where $\mathcal{A} = \cup_{t=1}^{M} \mathcal{A}(P_t)$ for preliminary copy number profiles $P_1, \dots, P_M$ guiding the optimization. In practice, our implementation first samples some $[c^1, \dots, c^r] \in \mathcal{A}$(P) (for some P in our set of allowed copy number profiles) and then automatically samples all $[c^1, \dots, c^k]$ with $k \leq r$. The probability of such a generating sequence $[c^1, \dots, c^k]$ is still proportional to $\Pr(c^1, \dots, c^k \mid \mathcal{A}(P), \theta)$ (but with some normalization constant).

A.3.8 Comparison of our reinforcement learning method to existing approaches

We utilize the policy gradients approach with changing rewards during training in order to maximize the overall probability $\Pr(\mathbf{R}, \mathbf{B} \mid \mathbf{\Sigma}^R, \mathbf{\Sigma}^B, \theta)$ of all cells observed read counts data $\mathbf{R}$ and $\mathbf{B}$ given the model parameters θ. This approach balances the probabilities of different trajectories (generative procedures G)

Method	GFlowNets [7]	Our policy gradients approach	Standard policy gradients
**Goal**	Terminal state prob. $\Pr(P \mid \theta)$ proportional to rewards $R(P)$	Maximize overall prob. $\Pr(\mathbf{R}, \mathbf{B} \mid \mathbf{\Sigma}^R, \mathbf{\Sigma}^B, \theta)$ across cells	Maximize expected reward $\sum_G P(G \mid \theta) R(G)$
**Optimization approach**	Optimizing flows	Policy gradients with changing rewards	Policy gradients with fixed rewards
**Learned model**	Flows proportional to transition probabilities between states	Action prob. given a state yielding transition prob. between states	Action prob. given a state yielding transition prob. between states

Table S2: Comparison of our reinforcement learning approach with existing approaches

and terminal states (copy number profiles P) rather than maximizing the probability of the highest reward trajectory. One existing approach for balancing the probabilities of multiple beneficial trajectories is GFlowNets [7]). However, instead of maximizing some overall data probability, GFlowNets optimize the probability of terminal states to be proportional to some fixed reward (assuming rewards only occur on terminal states as is the case for our problem). Instead of having reward functions known ahead of time for each copy number profile output, we have an objective of maximizing cell read count probabilities and must adapt the rewards during training to maximize this objective. Consequently, although GFlowNets share conceptual similarities with our objective, they do not directly apply. Instead, we choose to alter standard policy learning to have adaptive reward functions, enabling the maximization of our objective. We provide Table S2 comparing our policy gradients approach with GFlowNets and standard policy gradients.

A.4 Post-processing and validation

A.4.1 Determining clone sizes when not all cells have values for all bins

The results of SIGNALS on both datasets and CHISEL on the ovarian cancer dataset do not contain copy number values for some cells in each bin. Instead, some bins may have no copy number value for some cells due to having no SNPs. As suggested by the SIGNALS team, for most of our comparisons we fill in these missing values using neighboring bins. However, for analyzing clone sizes as well as the number of unique copy number profiles, we allow two cells to be in the same clone as long as they agree on all copy numbers for all bins for which values are provided. Specifically, to determine the set of clones, we initialize

the set of clones as empty and the set of cells to be put into clones as the full set of cells. Then, iteratively, if some cell does not match any existing clone (in terms of the copy numbers on bins for which there exist copy number values), a new clone is formed by that cell. If some cell does match an existing clone (in terms of the copy numbers on bins which have copy number values for both the cell and clone), then that cell is added to the clone. When a cell is added to a clone, the clone then has a value for any bin for which either the clone originally had a value, or the new cell being added had a value. This process is repeated until all cells are within clones. The number of unique copy number profiles is then equal to the number of clones. For SIGNALS predictions on both datasets, there exists no pair of cells that agree on the values of all bins for which both cells have values. Consequently, for SIGNALS, there exist no clones containing more than 1 cell, and each cell has a unique copy number profile.

A.4.2 Calculating the probability of SNV counts given copy numbers

The goal of this section is to describe a method for orthogonally validating CNAs using single-nucleotide variants (SNVs) that were present in the *most recent common ancestor* (MRCA) of all tumor cells. Such SNVs are also known as *truncal*, as they occur on the trunk of the tumor phylogeny transforming a normal cell into the MRCA. As stated in the main text, we determine an SNV to be truncal if it occurred on at least 4 of 5 sections on breast cancer patient S0, or if it occurred on all three samples of the ovarian cancer patient.

We distinguish two types of truncal SNVs. The first type is a truncal SNV that occurred prior to any CNA affecting its genomic locus. In other words, such a truncal SNV was introduced in a cell with copy number $(1, 1)$ at the SNV's locus. Consequently, if subsequent CNAs occurred at that locus resulting in a final copy number of $(X^{(1)}, X^{(2)})$ in the MRCA then one expects the probability of variant reads and reference reads for that SNV to either be $X^{(1)}/(X^{(1)} + X^{(2)})$ or $X^{(2)}/(X^{(1)} + X^{(2)})$, depending on which allele the SNV occurred on. The second type is a truncal SNV that occurred after the introduction of CNAs affecting its genomic locus. Let $(X^{(1)}, X^{(2)})$ be the final copy number of the SNV's locus in the MRCA. Then, the probability of variant reads and reference reads for that SNV is $x/(X^{(1)} + X^{(2)})$ where $x \in \{1, \ldots, X^{(1)} + X^{(2)}\}$. As discussed in the main text, and shown in Section B.6, the majority of truncal SNVs are of the first type in our data.

Let an SNV occur on some segment, and let the allele-specific copy number of that segment for cell s be $(X_s^{(1)}, X_s^{(2)})$ for the first and second allele, respectively. If one knew the SNV occurred on the first allele then one could estimate the probability of variant reads to be $X_s^{(1)}/(X_s^{(1)} + X_s^{(2)})$, and the probability of reference reads to be $X_s^{(2)}/(X_s^{(1)} + X_s^{(2)})$. Let v_s be the number of variant reads for cell s and r_s be the number of reference reads for cell s observed at the SNV locus. Then, the probability of observing these

reference reads and variant reads for cell s is the below equation assuming the SNV is on the A-allele

$$\Pr(v_s, r_s \mid X_s^{(1)}, X_s^{(2)}, \text{A-allele}) = \left(\frac{X_s^{(1)}}{X_s^{(1)} + X_s^{(2)}}\right)^{v_s} \left(\frac{X_s^{(2)}}{X_s^{(1)} + X_s^{(2)}}\right)^{r_s} c(r_s, v_s). \tag{55}$$

Here $c(r_s, v_s) = \binom{v_s}{v_s+r_s}$ is the binomial coefficient only dependent on r_s and v_s, but not the copy numbers of the cells. The probability of a set of variant reads and reference reads for all N cells for a given SNV then becomes

$$\Pr(\mathbf{v}, \mathbf{r} \mid \mathbf{X^{(1)}}, \mathbf{X^{(2)}}, \text{A-allele}) = \prod_{s=1}^{N} \left(\frac{X_s^{(1)}}{X_s^{(1)} + X_s^{(2)}}\right)^{v_s} \left(\frac{X_s^{(2)}}{X_s^{(1)} + X_s^{(2)}}\right)^{r_s} c(r_s, v_s). \tag{56}$$

Similarly, if we assume the SNV occurred on the B-allele we have the following.

$$\Pr(\mathbf{v}, \mathbf{r} \mid \mathbf{X^{(1)}}, \mathbf{X^{(2)}}, \text{B-allele}) = \prod_{s=1}^{N} \left(\frac{X_s^{(2)}}{X_s^{(1)} + X_s^{(2)}}\right)^{v_s} \left(\frac{X_s^{(1)}}{X_s^{(1)} + X_s^{(2)}}\right)^{r_s} c(r_s, v_s). \tag{57}$$

Since we do not know which allele the SNV occurred on, we take the maximum as below.

$$\Pr(\mathbf{v}, \mathbf{r} \mid \mathbf{X^{(1)}}, \mathbf{X^{(2)}}) = \max(\Pr(\mathbf{v}, \mathbf{r} \mid \mathbf{X^{(1)}}, \mathbf{X^{(2)}}, \text{A-allele}), \Pr(\mathbf{v}, \mathbf{r} \mid \mathbf{X^{(1)}}, \mathbf{X^{(2)}}, \text{B-allele})). \tag{58}$$

Thus, while we do not know if the SNV occurs on the maternal or paternal haplotype, we do enforce that the SNV must be either consistently on the maternal or consistently on the paternal haplotyple across all cells.

Taking the log probability ratio, i.e.,

$$\log \left[\frac{\Pr(\mathbf{v}, \mathbf{r} \mid \mathbf{X}^{\mathbf{(1)}}_{\text{CNRein}}, \mathbf{X}^{\mathbf{(2)}}_{\text{CNRein}})}{\Pr(\mathbf{v}, \mathbf{r} \mid \mathbf{X}^{\mathbf{(1)}}_{\text{Other}}, \mathbf{X}^{\mathbf{(2)}}_{\text{Other}})}\right] \tag{59}$$

between our method and another method determines how much an SNV supports our method vs another method. Note that the binomial coefficients $c(r_s, v_s)$ cancel out and do not need to be computed. Summing the log probability ratios across all truncal SNVs gives the total evidence for either method over the other, such that positive values indicate stronger support for our method versus the other method. We additionally perform bootstrapping on the set of SNVs to be summed, allowing us to obtain confidence intervals and quantify statistical significance.

However, one complication is that if a single read is said to come from an allele with copy number 0, i.e., either $X^{(1)} = 0$ or $X^{(2)} = 0$. In that case, all the probabilities go to 0 and the log probability ratio becomes infinite. To address this, we set a minimum probability of any reference of variant read to be 0.05 (and consequently the maximum probability to be 0.95) even if there is LOH.

A.4.3 Simulation set-up

The noise levels in the simulation are based on breast cancer patient S0. Consequently, the total number of 100kb bins and the number of 100kb bins in each chromosome is set to the same number $K = 27{,}283$ as in the real data. For each simulation, we define p_{fit} as the probability that each new clone will have increased fitness. Let P_1 be the normal copy number profile with $P_{1,i}^{(1)} = P_{1,i}^{(2)} = 1$ for all bins i. Alternatively in simulations with whole genome duplication, let $P_{1,i}^{(1)} = P_{1,i}^{(2)} = 2$ for all i. Define the fitness of this starting clone as $F_1^{\text{fit}} = 1$. We then iterative define our procedure of adding CNAs to clones. Given the existing clones with profiles $P_1, \ldots, P_k$ and fitness values $F_1^{\text{fit}}, \ldots, F_k^{\text{fit}}$, we first select the clone in which the CNA occurs. Specifically, we select clone s with probability $F_s^{\text{fit}}/(\sum_{s=1}^{k} F_s^{\text{fit}})$. Let a_{k+1} be the index number of the selected clone. Since the copy $(0, 0)$ occurs very rarely in cancer, we set the fitness F_{k+1}^{fit} of the new clone to $\exp(-1000)$ if the new clone contains the copy number $(0, 0)$. Otherwise, we randomly set F_{k+1}^{fit} to either $F_{a_k}^{\text{fit}}$ with probability $1 - p_{\text{fit}}$ or $2F_{a_k}^{\text{fit}}$ with probability p_{fit}. The higher the probability p_{fit}, the more likely the simulated tumor is to be dominated by a small set of large high fitness clones. With low values of p_{fit}, most cells tend to come from distinct small clones. We varied $p_{\text{fit}} \in \{1/6, 1/8, 1/10, 1/12, 1/14, 1/16, 1/20, 1/50, 1/100, 1/1000\}$ in our simulations, with lower values of p_{fit} resulting in more clonal expansion and thus larger clones and fewer unique copy number profiles. We initialize $P_{k+1} = P_{a_k}$ prior to applying the CNA. The chromosome that the CNA occurs on is selected with a probability proportional to the size of the chromosome. The parental haplotype on which the CNA occurs is selected uniformly at random. In breast cancer patient S0, it is common for CNAs to start or end at the start or end of a chromosome. Consequently, in our simulation, the start position has a 50% probability of being automatically set to the beginning of the chromosome. If the starting position is not automatically set to the beginning of the chromosome, the start position is instead selected uniformly at random within the chromosome. The end position has a 50% probability of being automatically set at the end of the chromosome. If the end position is not automatically set to the end of the chromosome, it is randomly selected from bins within the chromosome at or after the start position bin.

Let $c_{\text{hap}}, c_{\text{start}}, c_{\text{end}}$ be the haplotype number selected, the starting position, and the ending position, respectively. The CNA is randomly set to be either a deletion or an amplification (gain), each with 50% probability. If it is an amplification (gain), we set $P_{k+1,i}^{(c_{\text{hap}})} = P_{a_k,i}^{(c_{\text{hap}})} + 1$ for all i with $c_{\text{start}} \leq i \leq c_{\text{end}}$. Let ReLU be the ReLU function, such that $\text{ReLU}(x) = x$ for $x \geq 0$ and $\text{ReLU}(x) = 0$ for $x < 0$. If it is a deletion, set $P_{k+1,i}^{(c_{\text{hap}})} = \text{ReLU}(P_{a_k,i}^{(c_{\text{hap}})} - 1)$ for all i with $c_{\text{start}} \leq i \leq c_{\text{end}}$ to prevent negative copy numbers. With this, we have generated F_{k+1}^{fit} and P_{k+1} from $P_1, \ldots, P_k$ and $F_1^{\text{fit}}, \ldots, F_k^{\text{fit}}$. We continue this

procedure for 4000 CNAs and thus generate $P_1, \dots, P_{4001}$ and $F_1^{\text{fit}}, \dots, F_{4001}^{\text{fit}}$.

After the copy number profiles have been generated, we generate $n = 1000$ cells and their read depth and BAF measurements. Specifically, each cell is assigned to clone s with probability $F_s^{\text{fit}}/(\sum_{s'=1}^{4001} F_{s'}^{\text{fit}})$. The noise-free read depth for a cell with copy number profile $(P^{(1)}, P^{(2)})$ is then defined below where it is scaled to have an average value of 1.

$$\tilde{R}^{\text{true}} = \frac{P^{(1)} + P^{(2)}}{\sum_{i=1}^{K}(P_i^{(1)} + P_i^{(2)})} \tag{60}$$

The noise-free BAF is defined as $\tilde{B}^{\text{true}} = P^{(2)}/(P^{(1)} + P^{(2)})$, where the division is calculated element wise.

To generate the measured read depth and BAF, we must add noise. We do this based on the noise observed in breast cancer patient S0. For the BAF, the number $A_{i,s}^{(1)} + A_{i,s}^{(2)}$ of observed haplotype specific reads for each bin i in each cell s is set to the average number of reads in the corresponding bin i in breast cancer patient S0. Specifically, the number of reads for each of the two alleles is drawn from a binomial distribution, with the probability of B-allele reads set to the true BAF $\tilde{B}_{i,s}^{\text{true}}$, and the total number of binomial trials set to the total number $A_{i,s}^{(1)} + A_{i,s}^{(2)}$ of haplotype specific reads. Consequently, some bins have a higher level of noise as a result of having fewer haplotype specific reads, which occurs in real data due to differing numbers of SNPs in different bins of the genome.

Simulating noise in the read depth is made slightly more complex by the fact that there is known to be overdispersion relative to what one would expect given a simple Poisson distribution. Specifically, if one assumes the reads are drawn from a Poisson distribution with the number of reads proportional to the total copy number then the simulated noise levels would be extremely low and unrealistic. Instead, we model the noise level based on breast cancer patient S0 noise levels directly. However, properly estimating which variations in read depth are due to noise also requires removing variations in read depth due to changes in copy number. To avoid this complexity, we estimate noise levels utilizing normal cells. Specifically, section A in breast cancer patient S0 consists primarily of normal cells. After filtering out 10% of cells (221 cells) with read depths that appear to be non-normal, we are left with 1970 normal cells. As previously mentioned, $\tilde{R}_s$ is scaled to have an average value of 1. Consequently, for normal cells $\tilde{R}_s - 1$ isolates changes in read depth due to noise. We calculate D_i^R as the standard deviation in bin i as below.

$$D_i^R = \sqrt{\frac{1}{N}\sum_{s=1}^{N}(\tilde{R}_s - 1)^2} \tag{61}$$

The average per bin per cell read count for the breast cancer patient S0 R_{avg} is then calculated. Then, for each bin i and each cell s, we use a negative binomial distribution with mean $R_{\text{avg}}\tilde{R}_{s,i}$ and standard

deviation $D_i^R R_{\text{avg}}$. Since the number of successes parameter is an integer, we technically can not exactly match a given mean and standard deviation, so instead, we exactly match the mean and pick the parameters that closest match the desired standard deviation. This distribution then allows us to generate measured read depths $\tilde{R}_s$ for each cell.

B Supplementary results

B.1 VICTree and CHISEL with clonal predictions

In this section, we utilize our simulated data to benchmark VICTree and CHISEL's clonal predictions (in contrast to CHISEL's standard individual cell predictions). Specifically, CHISEL has an optional post-processing step of clustering cells into clones and then providing copy number predictions for these clones. For VICTree, we ran VICTree with default settings and bins of size 500kb. However, VICTree requires ploidy corrected read counts rather than raw read counts as an input. In practice, one would use VICTree in combination with some ploidy estimation method. However, to ensure VICTree is not limited by the accuracy of our choice of ploidy estimation algorithm, we simply provide VICTree with ground truth ploidy corrected read depth data. This provides an upper bound on the accuracy of VICTree assuming one has a perfect ploidy estimation algorithm (which is very much non-trivial).

As shown in Fig. S7a, on our simulations clonal CHISEL has a lower median accuracy of 0.847 than CNRein (median accuracy error of 0.957) while performing similarly as the original CHISEL (median accuracy error of 0.858). Similarly in Fig. S7b, clonal CHISEL has a higher median L1 error of 0.217 than CNRein (median L1 error of 0.0644) while performing similarly as the original CHISEL (median L1 error of 0.191). Fig. S7c shows the predicted number of unique copy number profiles is lower than the original CHISEL predictions but has near zero correlation with the ground truth number of unique copy number profiles. Specifically, clonal CHISEL's number of unique copy number profiles has a median percentage error of 0.686 and a correlation of 0.04 with the ground truth in contrast to CNRein's median percentage of 0.069 and correlation of 0.97.

Fig. S7d shows that clonal CHISEL produces less parsimonious solutions than the ground truth. Specifically, clonal CHISEL has a median parsimony of $110{,}829$ in comparison to the ground truth median parsimony of 736. Additionally, clonal CHISEL's parsimony values are negatively correlated with ground truth parsimony (Pearson correlation -0.07) in contrast to the 0.95 correlation between CNRein parsimony and the ground truth parsimony. In summary, on simulated data clonal produces much more simplified parsimonious solutions than ordinary CHISEL, however, it does not better match the ground truth data.

To perform comparisons with VICTree, we utilize total copy number predictions of each method. The extensions of accuracy and L1 error to the total copy number are very natural. For completeness, we repeat the original definition of accuracy.

$$\frac{1}{NK}\sum_{s=1}^{N}\sum_{i=1}^{K}\delta_{\text{acc}}(P_{s,i}^{(1)}, P_{s,i}^{(2)}, \overline{P}_{s,i}^{(1)}, \overline{P}_{s,i}^{(2)}). \tag{62}$$

The accuracy becomes the below modification of equation (62)

$$\frac{1}{NK}\sum_{s=1}^{N}\sum_{i=1}^{K}\mathbf{1}_{(P_{s,i}^{(1)}+P_{s,i}^{(2)})\neq(\overline{P}_{s,i}^{(1)}+\overline{P}_{s,i}^{(2)})}. \tag{63}$$

In this equation $\mathbf{1}_{(P_{s,i}^{(1)}+P_{s,i}^{(2)})\neq(\overline{P}_{s,i}^{(1)}+\overline{P}_{s,i}^{(2)})}$ equals 1 if $(P_{s,i}^{(1)}+P_{s,i}^{(2)})\neq(\overline{P}_{s,i}^{(1)}+\overline{P}_{s,i}^{(2)})$, and 0 otherwise.

Similarly, we repeat the definition of L1 error given in the main text.

$$\frac{1}{NK}\sum_{s=1}^{N}\sum_{i=1}^{K}\delta_{\text{L1}}(P_{s,i}^{(1)}, P_{s,i}^{(2)}, \overline{P}_{s,i}^{(1)}, \overline{P}_{s,i}^{(2)}). \tag{64}$$

For the L1 error metric we have the below modification of equation (64)

$$\frac{1}{NK}\sum_{s=1}^{N}\sum_{i=1}^{K}|P_{s,i}^{(1)}+P_{s,i}^{(2)}-\overline{P}_{s,i}^{(1)}-\overline{P}_{s,i}^{(2)}|. \tag{65}$$

On our simulations as shown in Fig. S7e-f, VICTree has a much lower median accuracy (0.700) and much higher median L1 error (0.472) than CNRein (0.958 and 0.064, respectively). VICTree especially struggles with whole genome duplications as analyzed in Section B.2. On the 10 simulation instances without WGD, VICTree has a median accuracy and L1 error of 0.887 and 0.115, respectively, which is closer to CNRein's accuracy and L1 error of 0.967 and 0.056, respectively, on these instances. Fig. S7c shows that VICTree underestimates the level of intra-tumor heterogeneity, similarly to clonal CHISEL but in contrast with CNRein, SIGNALS, or CHISEL. Specifically, the median percentage error for VICTree is 99% in contrast to CNRein's median percentage error of 6.93%. Fig.. S7d shows that VICTree produces extremely parsimonious solutions which is expected as a result of VICTree's evolutionary model. However, VICTree produces a much more parsimonious solution (median parsimony 196) than the ground truth (median parsimony 736) and misses true intra-tumor heterogeneity as also reflected by its underestimation of the number of unique copy number profiles. Additional benchmarking of VICTree on CNAsim simulations is performed in Section B.2.

B.2 CNAsim simulations

Our primary simulation results are based on our own simulator, which is designed to generate simulations with varying levels of intra-tumor heterogeneity and realistic noise levels. However, it is worth also vali-

dating our method on independent existing simulation software. For this purpose, we chose the CNAsim simulator [8]. Like our simulator, CNAsim generates a phylogeny starting with a normal cell by sequentially adding CNAs to clones. Unlike our simulations, CNAsim generates the total read counts as the sum of allele-specific read counts. However, on real data most reads do not contain SNPs resulting in the allele-specific read counts being far lower than the total read count. Additionally, unlike CNAsim simulations, our simulations contain a fitness parameter that easily allows for variation in intra-tumor heterogeneity without while maintaining realistic copy number profiles. Finally, CNAsim tends to have large numbers of small focal CNAs with fewer large CNAs (even when chromosomal CNA parameters are increased).

Using CNAsim, we generated simulations with five different levels of coverage $0.01\times$, $0.02\times$, $0.05\times$, $0.1\times$, and $0.2\times$. Additionally, we generated half of the simulations with placement type 0 (i.e., '`--placement-type 0`') such that the expected number of CNAs on each edge is constant. The other half of our simulations use placement type 1 and placement parameter 0.1 (i.e., '`-placement-type 1 --placement-param 0.1`') such that the number of events are scaled according to edge lengths generated under the coalescent model with the mean number of events above leaf edges equal to the placement-param. We also generated half of the simulations with a whole genome duplication and half without a whole genome duplication. In total, there are $5 \cdot 2 \cdot 2 = 20$ combinations of these parameters, resulting in 20 simulation instances. As was used for our own simulations, 100kb bins were used to generate the CNAsim simulations.

We measure the allele-specific L1 error on a simulation instance exactly as was done on our simulation instances. Fig. S8a shows the L1 error for CNRein, SIGNALS, CHISEL, and VICTree on all CNAsim simulation instances. The error calculation for VICTree uses the total copy number not allele-specific copy number as defined in Section B.1. CNRein has a median L1 error of 0.049, somewhat outperforming CHISEL (median L1 error of 0.123), and greatly outperforming SIGNALS (median L1 error 0.471) and VICTree (median L1 error 0.990). The VICTree L1 errors clearly cluster into a group of high and low error simulation instances (Fig. S8a) with high errors corresponding to simulations with whole genome duplications. Consequently, we also measured L1 error on the subset of 10 simulations without whole genome duplication as shown in Fig. S8b. On these simulations, CNRein has a median L1 error of 0.0406, slightly outperforming VICTree (median L1 error of 0.0556) and while greatly outperforming SIGNALS (median L1 error of 0.1832) and CHISEL (mean L1 error of 0.2129). Also, note that CNRein errors include allele-specific errors while VICTree L1 errors only contain total copy number errors. Finally, Fig. S8c shows the average L1 error across CNAsim simulations for differing levels of coverage for each method. In all cases, CNRein achieves the lowest L1 error. However, the error of most methods including CNRein

increases as the coverage decreases. In summary, CNRein outperforms existing methods on independent CNAsim simulations confirming the advantages found on our novel simulations.

B.3 Detection of small CNAs

In Main text: "Evaluation on simulated scDNA-seq data", we analyzed the general accuracy of CNRein and CNNaive on simulated data. However, here we specifically analyze the accuracy of these methods in detecting small focal CNAs. These small CNAs can easily be overwhelmed by noise, causing copy number calling to be difficult to accomplish. Let $\overline{P_1}, \ldots, \overline{P_N}$ be the ground truth copy number profiles. We define the length of a CNA segment as the number of 100kb bins for which the copy number remains constant. Mathematically, let $L^{\text{after}}(s,i) = \text{argmin}_{j>i}(\overline{P}_{s,i} \neq \overline{P}_{s,j})$. Let $L^{\text{before}}(s,i) = \text{argmax}_{j<i}(\overline{P}_{s,i} \neq \overline{P}_{s,j})$. The length of the copy number segment containing i in cell s is $L^{\text{length}}(s,i) = L^{\text{after}}(s,i) - L^{\text{before}}(s,i) - 1$.

We now define the set of bins in small CNAs, medium CNAs, and large CNAs. Small CNAs are defined as having a length under 5Mb. Medium CNAs are defined as having a length at least 5Mb but under 20Mb. Large CNAs are defined as having a length at least 20Mb. We now mathematically define the sets of bins belonging to small, medium, and large CNAs for each simulation instance. For simulation number ζ, let $S_\zeta^{\text{small}} = \{(s,i) \mid L^{\text{length}}(s,i) < 50\}$, $S_\zeta^{\text{medium}} = \{(s,i) \mid 50 \leq L^{\text{length}}(s,i) < 200\}$, and $S_\zeta^{\text{large}} = \{(s,i) \mid \leq L^{\text{length}}(s,i) \geq 200\}$. Then S_ζ^{small} contains bins in CNAs smaller than 5Mb, S_ζ^{medium} contains bins in CNAs between 5Mb and 20Mb, and S_ζ^{large} contains bins in CNAs at least 20Mb. We then measure the L1 error on these sets of bins. As defined in Main text: "Evaluation details", $\delta_{\text{L1}}(a,b,a',b') = |\max(a,b) - \max(a',b')| + |\min(a,b) - \min(a',b')|$. We then calculate the average L1 error across all bins belonging to CNAs of each size across all simulation instances. Pooling across all 20 simulations gives the following unordered allele-specific error measurement on small CNAs.

$$\frac{\sum_{\zeta=1}^{20} \sum_{(s,i) \in S_\zeta^{\text{small}}} \delta_{\text{L1}}(P_{s,i}^{(1)}, P_{s,i}^{(2)}, \overline{P}_{s,i}^{(1)}, \overline{P}_{s,i}^{(2)})}{\sum_{\zeta=1}^{20} |S_\zeta^{\text{small}}|}. \tag{66}$$

Identical equations but with "small" replaced by "medium" and "large" give the error on medium and large CNAs respectively.

As shown in Fig. S4a, CNRein achieves an unordered allele-specific copy number error of 0.61, 0.27, and 0.084 on small, medium, and large CNAs respectively. In contrast, SIGNALS has a much higher error of 1.25, 0.97, and 0.42 on small, medium and large CNAs respectively. CHISEL has a larger error than CNRein on medium and large CNAs (0.54 and 0.24, respectively) but a similar error on small CNAs (0.62).

We then calculate the haplotype-specific error which considers the order of the two haplotypes. For this error metric, the copy numbers (X_1, X_2) and (X_2, X_1) are considered distinct with an L1 distance between

them of $2|X_2 - X_1|$. More precisely, the haplotype-specific error measurements are calculated as follows.

$$\frac{\sum_{\zeta=1}^{20} \sum_{(s,i)\in S_{\zeta}^{\text{small}}} (|P_{s,i}^{(1)} - \overline{P}_{s,i}^{(1)}| + |P_{s,i}^{(2)} - \overline{P}_{s,i}^{(2)}|)}{\sum_{\zeta=1}^{20} |S_{\zeta}^{\text{small}}|}. \tag{67}$$

However, since the existing methods SIGNALS and CHISEL modify the phasing of their inputs, evaluating them directly on this haplotype-specific error is unfair. Consequently, we post-process the outputs of both of these existing methods to match the ground truth phasing before comparisons. This gives a lower bound of their haplotype-specific error. Specifically, to accomplish this, for each bin i we swap the values of $P_{s,i}^{(1)}$ and $P_{s,i}^{(2)}$ for all cells s if that reduces the L1 error. CNRein's predictions do not receive this modification or any information from the ground truth copy number profiles. As shown in Fig. S4b, CNRein achieves the lowest errors for small, medium, and large CNAs (0.62, 0.27, and 0.084, respectively), whereas CHISEL has larger errors (0.68, 0.59 and 0.28, respectively) and SIGNALS has the highest errors (1.27, 0.98 and 0.43, respectively).

B.4 Accuracy in detecting small clones

CNRein accurately reconstructs the level of intra-tumor heterogeneity on simulations as demonstrated in Main text: "Evaluation on simulated scDNA-seq data". Here we further analyze CNRein's ability to reconstruct small clones, including clones of size 1 (cell-specific copy number profiles). For each cell s in simulation ζ define $C_{\text{size}}(s,\zeta)$ as the ground truth size of the clone cell s belongs to. Specifically, this is equal to the number of cells with the same exact copy number profile as s (including s itself). For each cell s in simulation ζ similarly define $C_{\text{size}}^{\text{pred}}(s,\zeta)$ as the size of the clone cell s belongs to according to the predicted copy number profiles. We can then define the set of predicted clone size for each ground truth clone size C as follows.

$$S_{\text{clone}}(C) = \{C_{\text{size}}^{\text{pred}}(s,\zeta) \mid \text{for all cells } s \text{ and simulation instances } \zeta \text{ with } C_{\text{size}}(s,\zeta) = C\} \tag{68}$$

We calculate the median of this set for ground truth clone sizes from 1 to 10 for CNRein, SIGNALS, and CHISEL. If CNRein missed small clones and cell-specific events in order to produce more parsimonious solutions, these values would be vast overestimated for small clone sizes. However, as shown in Fig. S6a, CNRein accurately reconstructs the ground truth small clone sizes including cell-specific events. Specifically, for all analyzed clone sizes other than 8, the median predicted clone size is exactly equal to the ground truth clone size. For ground truth clone size 8, the median predicted clone size is 7. In stark contrast, the median predicted clone size is always 1 for SIGNALS and CHISEL, independent of the ground truth. Although CNRein predicts the correct clone size on average, CNRein is not perfect resulting in some variation

around the ground truth clone size. Specifically, Fig. S6b shows the distribution of predicted clone sizes $S_{\text{clone}}(C)$ for each ground truth clone size from 1 to 10.

B.5 Benchmarking modifications of CNRein

In this section, we benchmark modifications to CNRein to measure the sensitivity of CNRein to modifications of its algorithm as well as the ability of our benchmarking to measure these effects. First, we analyze the effect of inaccurate noise estimates on CNRein's predictions for breast cancer patient S0 and ovarian cancer patient OV2295. Then, we analyze the sensitivity of our SNV-based orthogonal validation to cell-specific copy number profiles. Specifically, we do so by arbitrarily removing all cell-specific copy number profiles from CNRein's predictions (on ovarian cancer patient OV2995 which has by far the most cell-specific predictions) and observing how that impacts fit to SNVs. Finally, we evaluate the impact on CNRein of excessively small segments induced by reducing the minimum segment size in our segmentation algorithm (on breast cancer patient S0 and ovarian cancer patient OV2295).

CNRein uses estimates Σ^B and Σ^R of the variance in the read depth and BAF in each segment in order to calculate the probability of different copy number profiles. Our model aims to balance fitting each cell's read count data with realistic evolutionary constraints. Specifically, our model learns to balance these factors starting from initial estimates we refer to as CNNaive that do not consider evolutionary constraints. On ovarian cancer patient OV2295, CNRein predictions have a median L1 difference of 0.0868 with CNNaive predictions, which is similar to the median L1 distance of 0.08769 between CNRein and SIGNALS. Inaccurately high variance estimates may disrupt learning of CNRein predictions from CNNaive predictions, specifically put higher emphasis on finding coherent trajectories across at the sacrifice of fitting individual cells data. To investigate this, we utilize a modified version of CNRein that artificially multiplies all variance estimates by a factor of 10, and appy this approach to breast cancer patient S0 and ovarian cancer patient OV2295. As shown in Fig. S21a and Fig. S31a, modified CNRein performs slightly worse than the original CNRein on both patients. On ovarian cancer patient OV2295, the original log likelihood ratios between CNRein and SIGNALS, CHISEL and Alleloscope ($-1{,}033.42$, 3,418.66, and 12,635.36, respectively) very slightly worsen to $-1{,}346.08$, 3,105.99, and 12,403.77, respectively (with p values $2 \cdot 10^{-5}$, 10^{-5}, and 10^{-5}, respectively). On breast cancer patient S0, the original log likelihood ratios between CNRein and SIGNALS, CHISEL and Alleloscope (145.26, $1,093.83$, and -35.24, respectively) very slightly worsen to 79.94, 1018.90, and -61.38, respectively (with p values 10^{-5}, 0.125, and 0.067, respectively). As shown in Fig. S21b, and Fig. S31b, the parsimony score for the modified CNRein is 2,100 and 209 on ovarian cancer patient OV2295 and breast cancer patient S0 respectively. As expected, this parsimony is reduced

compared to the original parsimony scores of 7,163 and 397 on ovarian cancer patient OV2295 and breast cancer patient S0 respectively. Overall, artificially increasing the variance estimates puts more emphasis on forming a coherent solution across cells (as reflected by parsimony) but slightly worsens the fit to the data (as reflected by SNV-based analysis).

SNV-based orthogonal validation is a major source of benchmarking on our real data so its worth evaluating its sensitivity to cell-specific copy number profiles. Relatively low SNV counts make it difficult to evaluate subtle changes in copy number with SNV-based orthogonal validation. Therefore, we use patient OV2295 (which has the most cell-specific profiles) and perform the substantial modification to CNRein's predictions of removing all cell-specific profiles (rather than subtly reducing the number of cell-specific profiles). Specifically, after we remove all cell-specific copy number profiles, we reassign cells that formerly had cell-specific copy number profiles to the closest L1 distance copy number profile that exists in multiple cells. As shown in Fig. S18, removing cell-specific copy number profiles resulted in CNRein having a worse fit with orthogonal SNV data. This demonstrates the sensitivity of the orthogonal SNV-based analysis to missing all cell-specific copy number profiles. For reference, Fig. S18 also includes our previous comparisons of the unmodified CNRein predictions with SIGNALS and CHISEL.

Another modification to CNRein is to decrease the size of segments by decreasing the minimum region size Γ used for finding segments in the algorithm of Section A.1.3. Specifically, we tried including $\Gamma = 5$ (resulting in 500kb segments) in addition to the existing $\Gamma = 10$ (for 1Mb or larger segments), $\Gamma = 20$ and $\Gamma = 40$ already considered by our method. As shown in Fig. S22a and Fig. S32a this does not create any large scale visible difference in the copy number profiles for either breast cancer pateint S0 or ovarian cancer patient OV2995. Applying the orthogonal SNV-based analysis keeps the same relative performance of methods as before the modification (Fig. S22b and Fig. S32b) for both ovarian cancer patient OV2295 and breast cancer patient S0. However, on ovarian cancer patient OV2295, the quality of CNRein's predictions relative to other methods is slightly reduced. Specifically, the log likelihood ratios are less favorable to CNRein for comparisons with SIGNALS (from $-1,033$ to $-2,418$), CHISEL (from $3,418$ to $2,033$) and Alleloscope (from $12,635$ to $11,582$). As shown in Fig. S22 and Fig. S32c, reducing the size of segments also increases the parsimony scores for CNRein. On ovarian cancer patient OV2295, the modified CNRein has a parsimony score of 19,348, compared to an original parsimony score of 7,163, SIGNALS' parsimony score of 34,529, CHISEL's parsimony score of 13,769, and Alleloscope's parsimony score of 4,149. On breast cancer S0, the modified CNRein has a parsimony score of 379, compared to an original parsimony score of 397, SIGNALS' parsimony score of 40,222, CHISEL's parsimony score of 8,506, and Alleloscope's parsimony score of 2,076. Overall, decreasing segment sizes by including $\Gamma = 5$ seems to slightly worsen

the predictions of CNRein, increasing the parsimony score for ovarian cancer patient OV2295 and slightly worsening the fit to SNVs for both datasets.

B.6 VAF analysis on additional copy numbers

As stated in the main text, we use the set notation $\{X^A, X^B\}$ to refer to either of the copy numbers (X^A, X^B) or (X^B, X^A). In the main text, we plotted the variant allele frequency (VAF) of truncal SNVs that occur on cells with the copy number $\{2, 1\}$ inferred by CNRein and Alleloscope for ovarian cancer patient OV2295 (Main text: Fig. 3f-g). Here, we provide similar VAF plots for other copy numbers inferred by CNRein, SIGNALS, CHISEL, and Alleloscope. Note for VAF plots, to avoid excessive noise, we restrict to SNVs with at least 20 total reference and variant reads. For ovarian cancer patient OV2295, Fig. S15 shows VAF plots for CNRein, SIGNALS, CHISEL, and Alleloscope on several copy numbers. Specifically, all copy numbers with at least 300 SNVs for all methods are shown, in addition to the copy number $\{2, 2\}$ which has at least $1{,}000$ SNVs for all methods other than Alleloscope. For copy number $\{1, 2\}$, Alleloscope's VAFs form a unimodal distribution concentrated around $1/2$, in contrast to all other methods predicting the expected bimodal distribution with peaks around $1/3$ and $2/3$. Additionally, for $\{1, 1\}$ and $\{1, 2\}$, Alleloscope's VAFs have a large spike at 1.0, indicating loss of heterozygosity incorrectly being predicted as $\{1, 1\}$ and $\{1, 2\}$. CHISEL also has a much smaller spike in the VAF at 1.0 for $\{1, 2\}$, whereas CNRein and SIGNALS do not have this issue. For the copy number $\{2, 2\}$, CNRein, SIGNALS, and CHISEL all have a unimodal distribution centered at $1/2$, rather than having peaks at $1/4$ or $3/4$. This indicates SNVs occurring prior to CNAs or WGD. Additionally, for all methods, copy number $\{1, 2\}$ is predicted to have allelic mirroring in which some cells have copy number $(1, 2)$ whereas other cells have copy number $(2, 1)$ for the same genomic region. For any SNV on haplotype A, the VAFs on copy numbers $(1, 2)$ and $(2, 1)$ are expected to be near $1/3$ and $2/3$, respectively as shown in Fig. S13. Similarly, for any SNV on haplotype B, the VAFs on copy numbers $(1, 2)$ and $(2, 1)$ are expected to be near $2/3$ and $1/3$, respectively. However, in either case, the VAFs on copy numbers $(1, 2)$ and $(2, 1)$ should not be both near $1/3$ nor should they be both near $2/3$. Fig. S14 shows the VAFs of SNVs (with at least 10 reads) on $(1, 2)$ and $(2, 1)$ for CNRein, SIGNALS, CHISEL, and Alleloscope, demonstrating agreement with SNVs for CNRein, SIGNALS, and CHISEL, but not Alleloscope. Specifically, the Pearson correlation between the VAF for $(1, 2)$ and $(2, 1)$ is -0.65, -0.72, -0.67, and -0.05 for CNRein, SIGNALS, CHISEL, and Alleloscope, respectively, where a strong negative correlation validates predicted allelic mirroring.

In addition to these common copy numbers, we investigated a wide variety of additional copy numbers predicted by CNRein. In order to maximize the number of observed SNVs, we used all $n = 890$ cells,

rather than the subset of $n = 617$ cells for which all methods have predictions. Fig. S15 shows VAFs for the top-11 copy numbers with the most SNVs (or equivalently, all copy numbers with at least 140 SNVs occurring on that copy number), as well as the copy number $\{3, 3\}$ (which has 64 SNVs but is still very visually clear due to the simple unimodal VAF structure). For all copy numbers $\{X^A, X^B\}$ there are peaks at $X^A/(X^A + X^B)$ and $X^B/(X^A + X^B)$ as expected. For the sake of completeness, we also include the VAF plots for SIGNALS (Fig. S16) and CHISEL (Fig. S17).

On breast cancer patient S0, we show VAF plots for the $n = 3{,}540$ cells for which CNRein, SIGNALS, and CHISEL give predictions. We excluded Alleloscope since further restricting to $n = 785$ cells in one section would substantially reduce the SNV read counts making VAF plots much less visually clear. Fig. S29 shows VAF histograms for copy numbers whose corresponding segments contain at least 28 truncal SNVs for CNRein, SIGNALS, and CHISEL. For all methods and all copy numbers $\{X^A, X^B\}$, there are peaks at $X^A/(X^A + X^B)$ and $X^B/(X^A + X^B)$ as expected. For copy number $1, 2$, CHISEL seems to have a less clear bimodal distribution than CNRein and SIGNALS, as a result of having more VAFs near $1/2$ and more VAFs near 1.0. Additionally, CNRein, SIGNALS, and CHISEL all predicted allelic mirroring for copy number $\{1, 2\}$. As previously described, SNV validation of allelic mirroring would result in the VAFs for $(1, 2)$ and $(2, 1)$ containing one value near $1/3$ and one value near $2/3$ (but not both values near $1/3$ nor both values near $2/3$). This pattern validating the predicted allelic mirroring is clear for CNRein and SIGNALS but not CHISEL as shown in Fig. S30. Specifically, the Pearson correlation between the VAF on $(1, 2)$ and $(2, 1)$ is -0.89, -0.89, and -0.20 for CNRein, SIGNALS, and CHISEL respectively, where a strong negative correlation demonstrates SNV validation of allelic mirroring. This result is in line with our statistical analysis on SNVs showing that CHISEL matches SNV data worse than CNRein and SIGNALS (described in Main text: "Evaluation on a breast cancer dataset sequenced with 10x Chromium CNV technology").

B.7 Comparing copy numbers with read depth

In addition to SNV-based orthogonal validation, we also validate CNRein utilizing fit to read depth as shown in Main text: "Evaluation on an ovarian cancer dataset sequenced with DLP+ technology" and Main text: "Evaluation on a breast cancer dataset sequenced with 10x Chromium CNV technology". Here we provide the details of this analysis in addition to a read depth analysis on specifically cell specific CNAs.

To analyze fit to read depth, we divide the total copy number for each cell by its average value across bins to produce a normalized average copy number. We then also divide the read depth for each cell by its average value across bins to form a normalized read depth. We define the L1 read depth error for cell s as

the average L1 error for all bins i between the normalized predicted total copy number and the normalized read depth. Main text: Fig. 3c shows the L1 read depth errors for CNRein, SIGNALS, CHISEL, and Alleloscope on ovarian cancer patient OV2295. Applying the student's T-test on the set of errors demonstrates CNRein's predictions fit the read depths better than CHISEL or Alleloscope with p values $7.4 \cdot 10^{-30}$, and $3.9 \cdot 10^{-278}$, respectively. There is no statistically significant difference between the L1 distance for CNRein and SIGNALS. Main text: Fig. 4c shows the L1 read depth errors for CNRein, SIGNALS, CHISEL, and Alleloscope on breast cancer patient S0. Again, CNRein fits the read depths better than CHISEL and Alleloscope (p values $2.5 \cdot 10^{-6}$ and 0.0043, respectively), but there is no statistically significant difference between the L1 distance for SIGNALS and CNRein. Overall, these analyses validate the conclusion that CNRein's parsimonious solutions do not come at the cost of fitting the read depth data and detecting legitimate CNAs.

In addition to analyzing fit to read depth generally, it is also worth investigating cell-specific copy number profiles and cell-specific CNAs. We specifically analyze this on ovarian cancer OV2295 since this patient has by far the most cell-specific copy number profiles according to CNRein (408 out of 617 cells in comparison to 113 out of 783 cells on breast cancer patient S0). In contrast, SIGNALS predicts all cells to have cell-specific profiles on this dataset. Therefore, it is worth considering if the set of cells we choose to have or not have cell-specific profiles is justified by the read-depth data. We analyze the L1 distance between the normalized read depth of each pair of cells. Then for each cell, we calculated the minimum L1 distance to any other cell. One would expect cells with the same copy number profile to have lower L1 distances between their read depths on average. Consequently, one would expect cells belonging to clones with shared copy number profiles to have a lower minimum L1 distance than cells with cell-specific copy number profiles. As shown in Fig. S19, this is exactly what we see. Specifically, applying the t-test shows larger L1 distances for cells with predicted cell-specific profiles with a p value of $p = 4.8 \cdot 10^{-30}$. This simple analysis provides general evidence of cell-specific copy number profiles, but does not specifically analyzed the read depth within the region of cell-specific CNAs.

We characterize CNAs as a region with some start position, end position, and allele-specific copy number. We then define a cell-specific CNA as a CNA that only occurs on one cell (with that start position, end position, and allele-specific copy number). For a given cell and predicted copy number profile, we define the scaled read depth as the read depth scaled to have the same average value as the average total copy number in the copy number profile. With this scaling, one expects the read depth to be on average equal to the copy number for the correct copy number profile. This allows us to investigate systematic biases in the scaled read depth relative to the predicted copy number due to the inference of spurious CNAs. Specif-

	Modified skewness			Total number of bins		
Copy Number	CNRein	SIGNALS	CHISEL	CNRein	SIGNALS	CHISEL
1	**0.71**	0.84	0.73	23,448	120,245	6,649
2	0.27	**0.09**	0.25	23,983	227,944	17,373
3	**-0.08**	-0.29	0.09	26,362	216,510	32,794
4	**0.03**	-0.09	0.06	30,668	146,715	27,142
5	**-0.18**	-0.26	-0.27	35,441	90,178	29,958
6	**-0.24**	-0.53	-0.44	22,723	58,172	18,751
7	**-0.42**	-0.58	-0.58	15,821	23,520	7,573
8	**-0.36**	-0.63	-0.82	8,010	10,859	5,599
9	**-0.44**	-0.64	-0.61	5,542	5,395	465
10	**-0.28**	-0.61	-0.34	3,563	3,289	1,450

ically, for each total copy number from 1 to 10, we observe the read depth of sets of bins (in specific cells) that are contained within predicted cell-specific CNAs with that total copy number. Fig. S19 shows the distribution of these scaled read depth values for CNRein SIGNALS, and CHISEL for copy numbers with at least $10,000$ bins in cell-specific CNAs for each method (which corresponds to copy numbers 2 through 6). We exclude Alleloscope from this analysis since differences in cell-specific CNAs are overshadowed by Allloscope predicting no WGD and thus extremely different copy numbers than all other methods across the entire genome. If predicted CNAs are legitimate, we expect the scaled read depths to form a distribution concentrated around the predicted copy number. However, if predicted CNAs come from random deviations from some ground truth copy number, we expected the read depth to be biased by this and to form a lopsided distribution. Skewness is defined by the difference in the mean from the median divided by the standard deviation and encapsulates lopsided distributions that are shifted away from thee median. Since we know the distribution should be concentrated around the predicted copy number (rather than some median), we calculate a modified skewness metric defined by the mean minus the predicted copy number divided by the standard deviation. The below table shows modified skewness values for all total copy numbers from 1 to 10.

We observe that CNRein has the lowest modified skewness for all copy numbers with the exception of 2, indicating CNRein suffers lopsided read depth distributions due to spurious CNAs less than existing approaches. However, the distributions for CNRein are still somewhat skewed, indicating some incorrect cell-specific CNAs and thus room for further improvement in copy number calling.

References

[1] Simone Zaccaria and Benjamin J Raphael. Characterizing allele-and haplotype-specific copy numbers in single cells with CHISEL. *Nature biotechnology*, 39(2):207–214, 2021.

[2] Petr Danecek, James K Bonfield, Jennifer Liddle, John Marshall, Valeriu Ohan, Martin O Pollard, Andrew Whitwham, Thomas Keane, Shane A McCarthy, Robert M Davies, and Heng Li. Twelve years of samtools and bcftools. *GigaScience*, 10(2):giab008, January 2021.

[3] Olivier Delaneau, Jean-François Zagury, Matthew R. Robinson, Jonathan L. Marchini, and Emmanouil T. Dermitzakis. Accurate, scalable and integrative haplotype estimation. *Nature Communications*, 10(1):5436, November 2019.

[4] Adam Auton, Gonçalo Abecasis, Richard Gibbs, Eric Boerwinkle, Harsha Doddapaneni, Yi Han, Viktoriya Korchina, Christie Kovar, Sandra Lee, Donna Muzny, Jeffrey G. Reid, Yiming Zhu, Eric S. Lander, David M. Altshuler, Stacey B. Gabriel, et al. A global reference for human genetic variation. *Nature*, 526(7571):68–74, 2015.

[5] Tyler Funnell, Ciara H O'Flanagan, Marc J Williams, Andrew McPherson, Steven McKinney, Farhia Kabeer, Hakwoo Lee, Sohrab Salehi, Ignacio Vázquez-García, Hongyu Shi, et al. Single-cell genomic variation induced by mutational processes in cancer. *Nature*, 612(7938):106–115, 2022.

[6] Josiah P Hanna, Scott Niekum, and Peter Stone. Importance sampling in reinforcement learning with an estimated behavior policy. *Machine Learning*, 110(6):1267–1317, 2021.

[7] Yoshua Bengio, Salem Lahlou, Tristan Deleu, Edward J Hu, Mo Tiwari, and Emmanuel Bengio. Gflownet foundations. *Journal of Machine Learning Research*, 24(210):1–55, 2023.

[8] Samson Weiner and Mukul S Bansal. CNAsim: improved simulation of single-cell copy number profiles and DNA-seq data from tumors. *Bioinformatics*, 39(7):btad434, 2023.

[9] Emma Laks, Andrew McPherson, Hans Zahn, Daniel Lai, Adi Steif, Jazmine Brimhall, Justina Biele, Beixi Wang, Tehmina Masud, Jerome Ting, et al. Clonal decomposition and DNA replication states defined by scaled single-cell genome sequencing. *Cell*, 179(5):1207–1221, 2019.

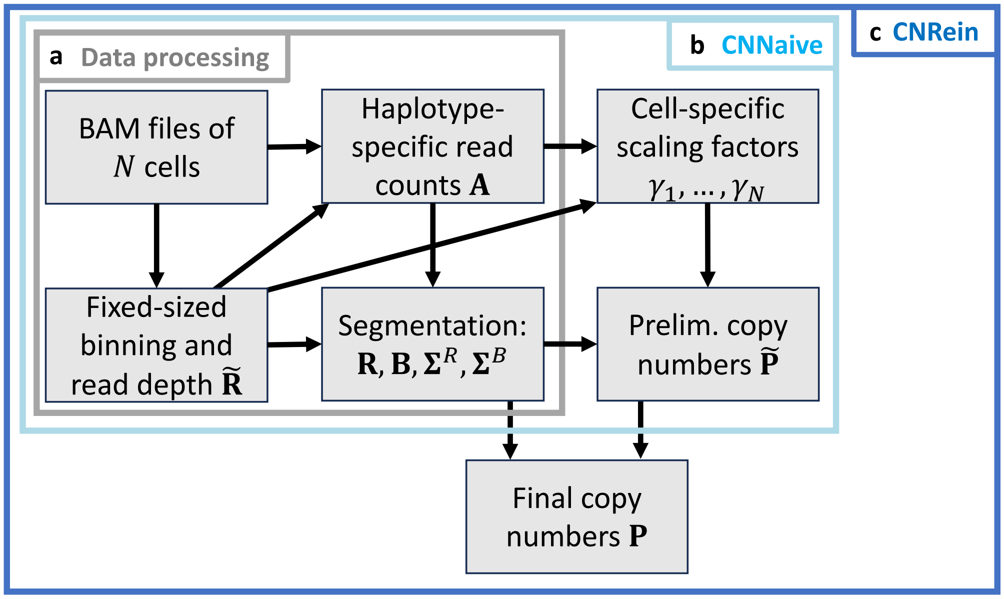

Figure S1: **A diagram of the processes in CNRein.** **a** Data processing steps to obtain measurements $\mathbf{R}, \mathbf{B}, \mathbf{\Sigma}^R, \mathbf{\Sigma}^B$ for L segments from BAM files of N cells. **b** Given these measurements, CNNaive produces preliminary copy number profiles $\tilde{\mathbf{P}}$ by identifying a cell-specific scaling factor γ_s for each cell s, without using an evolutionary model. **c** Finally, CNRein applies evolution-aware deep reinforcement learning given input measurements $\mathbf{R}, \mathbf{B}, \mathbf{\Sigma}^R, \mathbf{\Sigma}^B$ together with initial estimates of copy number profiles $\tilde{\mathbf{P}}$ to produce the final copy number profiles $\mathbf{P}$.

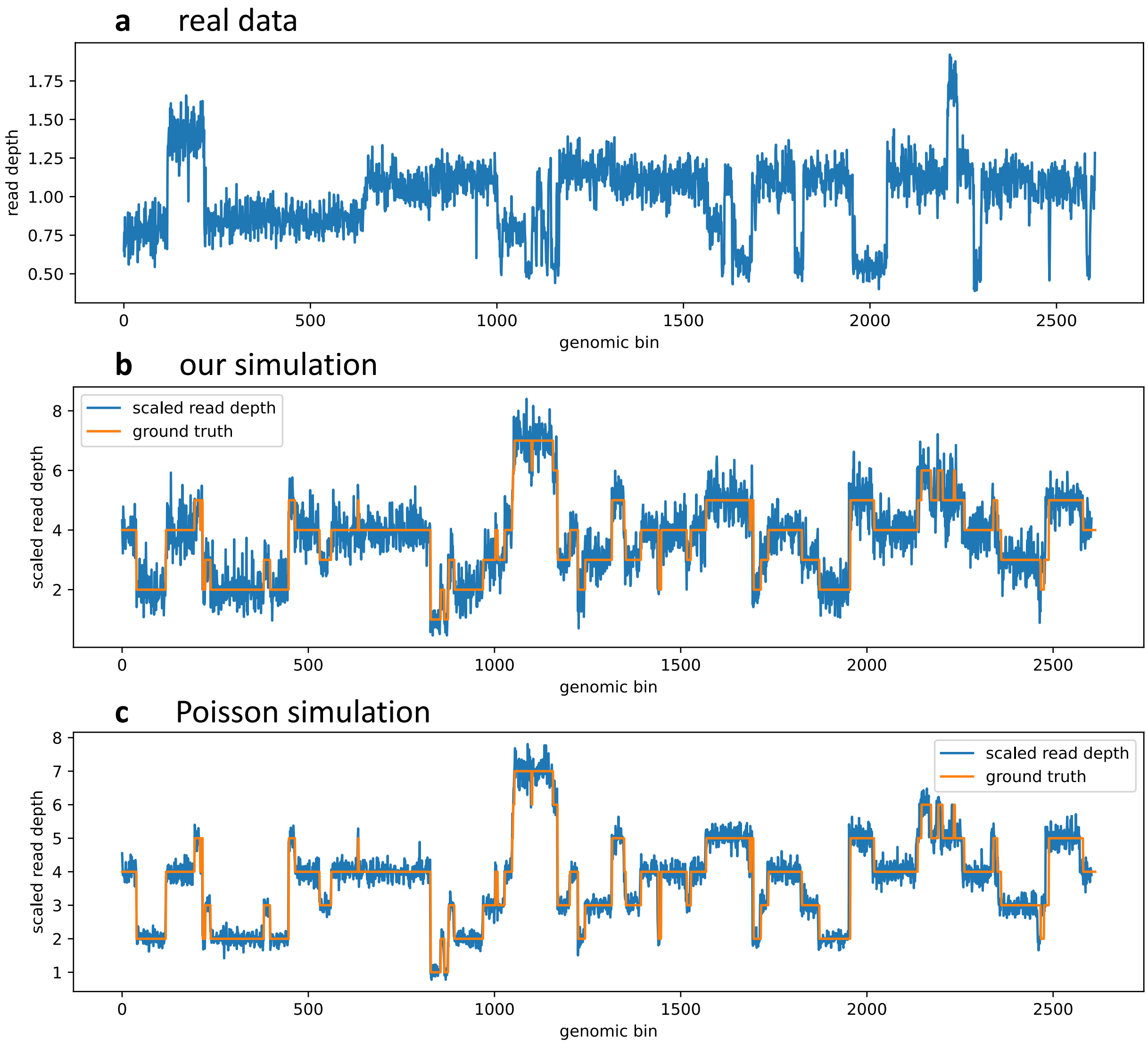

Figure S2: **A comparison of breast cancer patient S0 read depths and our simulated read depths.** Read depths on real and simulated data are shown. Bins of size 1Mb are used for the sake of visual clarity. **a** Read depths for a cell in breast cancer patient S0 are shown. These read depths are scaled to have an average value of 1. **b** Read depths are shown for one cell in our simulation instance. The read depths are scaled such that the average read depth equals the average copy number to allow an easy comparison with the ground truth. **c** A Poission noise-based read depth plot on the same simulated cell is generated. The number of reads used is equal to the average number of reads per cell breast cancer patient S0 (989,469 reads). These reads are then mapped to bins with a probability proportional to the total copy number of the bin. The read depths are scaled such that the average read depth equals the average copy number to allow an easy comparison with the ground truth. Despite using the same number of reads as in breast cancer patient S0, the noise level is still clearly far below the noise level of the measured read depths from breast cancer patient S0.

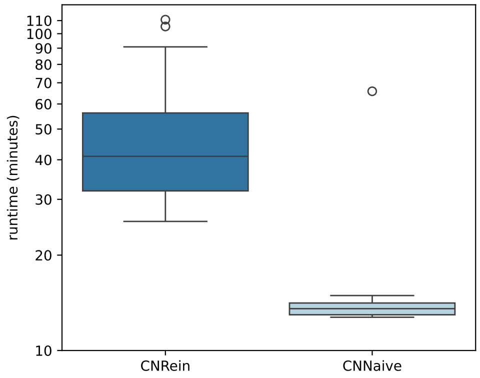

Figure S3: **Runtimes on** 20 **simulation instances for CNRein and CNNaive.** CNNaive steps starting from read depths $\tilde{\mathbf{R}}$ and haplotype-specific counts $\mathbf{A}$ (provided by the simulation) run fairly quickly. The runtime of CNRein's optimization ranges from 26 minutes to 111 minutes, whereas CNNaive's runtime ranges from 13 minutes to 66 minutes. CNRein (and CNNaive) were run on a laptop with 96GB of RAM and a 3.6 GHz processor (with 12 cores), without the use of a GPU for all experiments.

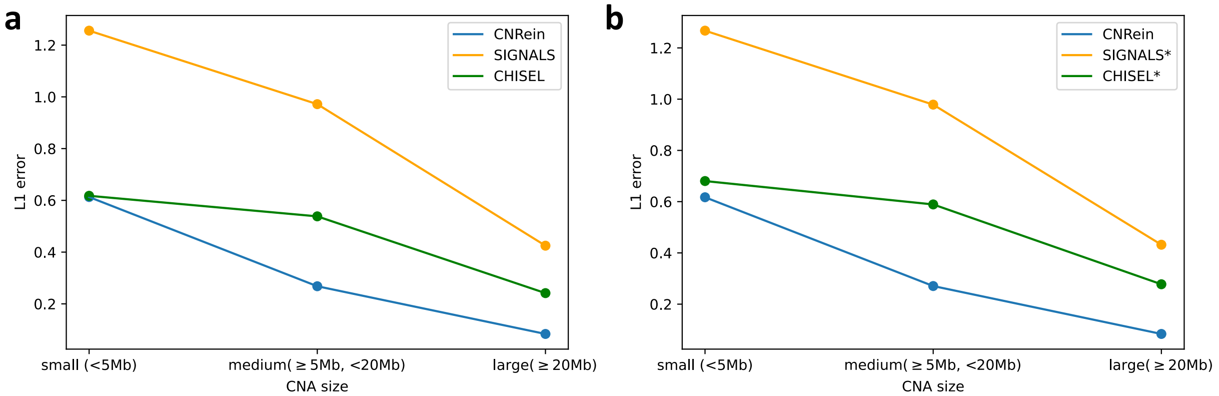

Figure S4: **The error of CNRein and CNNaive on small, medium, and large CNAs on simulated data. a** Unordered allele-specific L1 error (equation (67)). **b** Haplotype-specific L1 error. SIGNALS and CHISEL are given ground truth phasing.

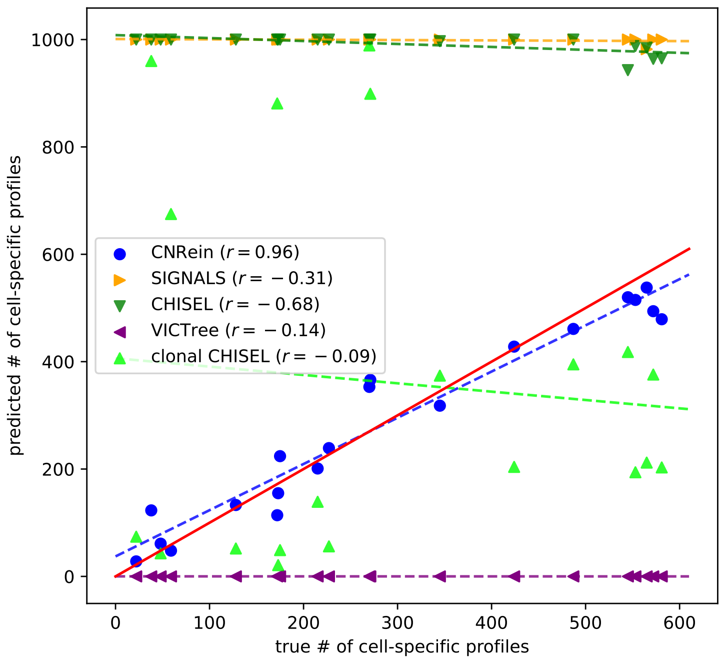

Figure S5: **CNRein accurately reconstructs the number of cell-specific profiles on simulated data.** In Main text: Fig. 2d, we found that CNRein accurately reconstructs the total number of unique copy number profiles on our 20 simulation instances. Here we observe the predicted and ground truth number of cell-specific copy number profiles. CNRein accurately reconstructs the number of cell-specific copy number profiles (Pearson correlation $r = 0.96$) unlike SIGNALS (Pearson correlation $r = -0.31$) or CHISEL (Pearson correlation $r = -0.68$). The red, solid line shows the $x = y$ line.

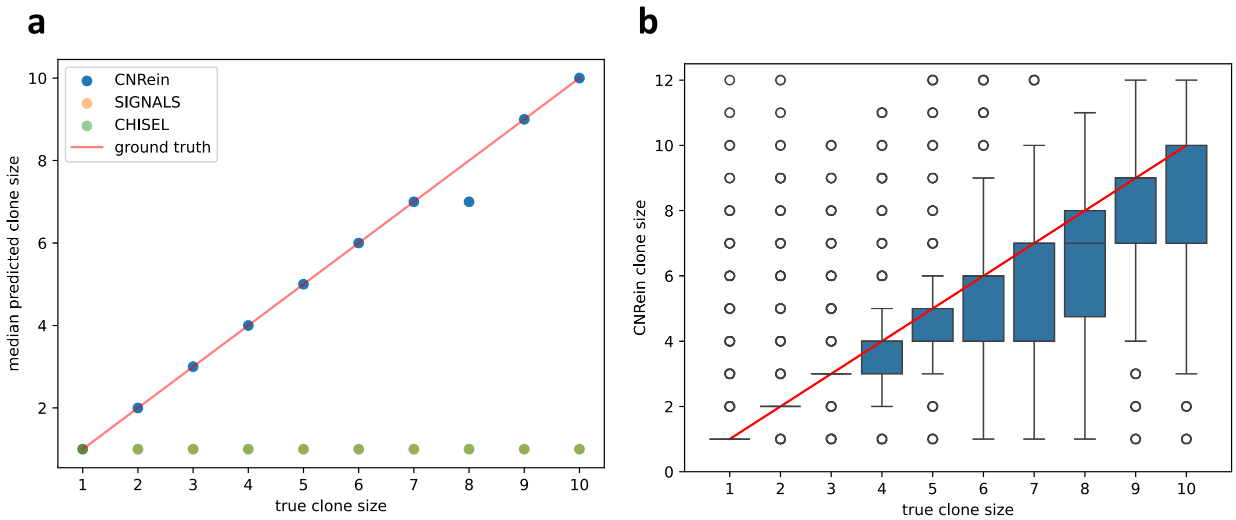

Figure S6: **CNRein accurately reconstructs small clones on simulations. a** Median predicted clone sizes for each ground truth clone size from 1 to 10 for CNRein, SIGNALS and CHISEL. CNRein accurately reconstructs clone sizes whereas SIGNALS and CHISEL predict median clone sizes of 1 independent of the true clone size. **b** The distribution of predicted clone sizes for CNRein is shown for each ground truth clone size. For all ground truth clone sizes other than 8, the median value and the third quartile value both equal the ground truth clone size.

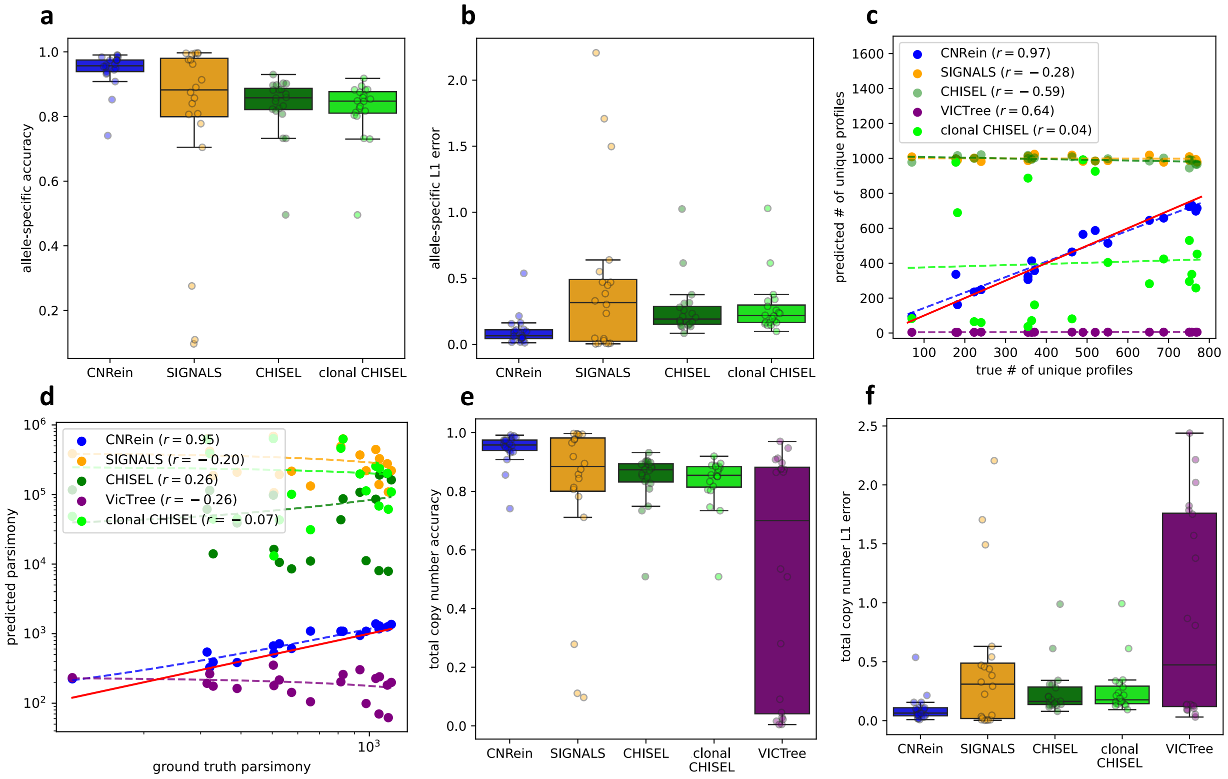

Figure S7: **Testing VICTree and clonal CHISEL on simulations.** By default, CHISEL produces predictions on individual cells. However, CHISEL also provides predicted clonal copy numbers which we refer to as clonal CHISEL. **a-b** Allele-specific accuracy and L1 error on our simulations. clonal CHISEL performs similarly well as the original CHISEL predictions, underperforming CNRein. **c** The ground truth and predicted number of unique profiles for each method including clonal CHISEL. The ground truth line is shown with a solid red line. clonal CHISEL produces much smaller numbers of unique copy number profiles than the ground truth. **d** Ground truth and predicted parsimony values are shown for each method. Clonal CHISEL overestimates the parsimony for low parsimony simulation instances and underestimates the parsimony for high parsimony simulation instances. Note that the line of best fit for clonal CHISEL appears very curved due to the logarithmic scale of both axes. **e-f** Total copy number accuracy and L1 error on our simulations.

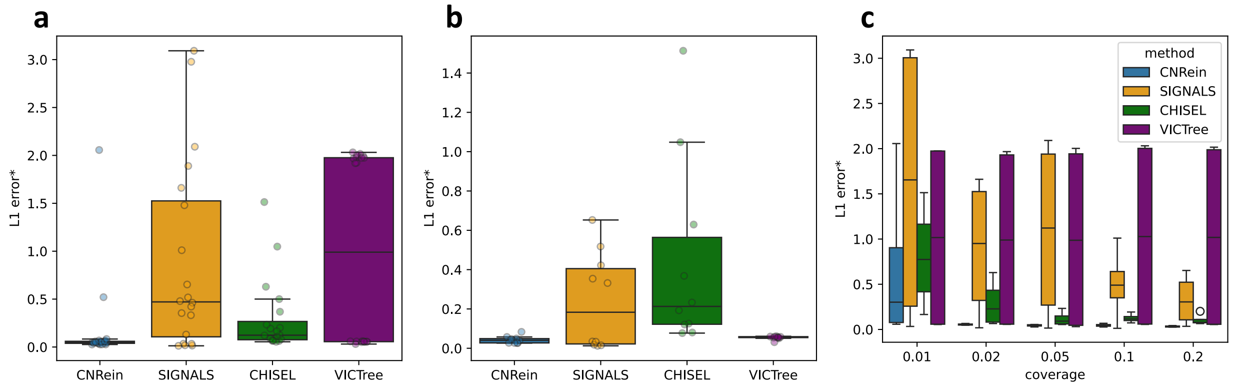

Figure S8: **Results on CNAsim simulations.** L1 error measurements of CNRein, SIGNALS, CHISEL and VICTree on CNAsim simulations. Since VICTree predicts total copy numbers, its L1 errors are for total copy numbers, whereas the allele-specific L1 errors are shown for the other methods. **a** The L1 error of each method on all 20 simulation instances. CNRein has the best performance followed by CHISEL. **b** The L1 error on the subset of 10 simulation instances without whole genome duplication. Although VICTree performs very poorly with whole genome duplications, VICTree has the second lowest median error by a large margin of 0.0556 after CNRein (median L1 error 0.0406) on the subset of simulations without WGD. **c** The average L1 error on simulations with each level of coverage. In all cases, CNRein performs the best followed by CHISEL. For both CNRein and CHISEL, the L1 error increases as the coverage decreases.

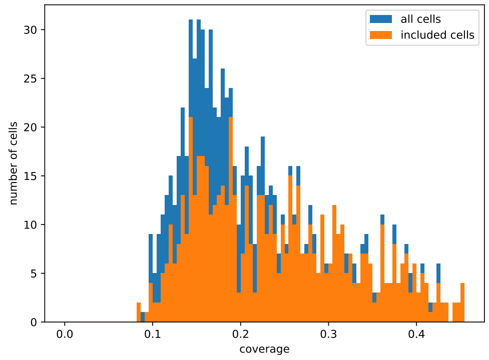

Figure S9: **The coverage of cells of ovarian cancer patient OV2295 [9].** A histogram of the coverage of cells. In blue is the set of all 890 available cells. In orange are the $n = 617$ cells for which all methods were run on and comparisons were performed.

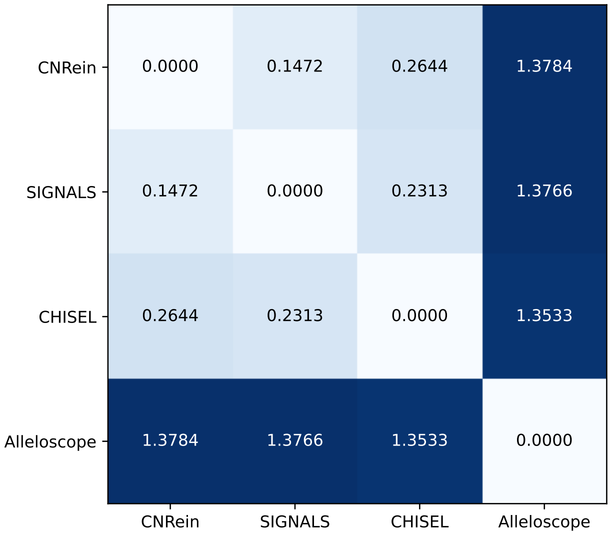

Figure S10: **L1 differences between copy number profiles predicted by CNRein, SIGNALS, CHISEL, and Alleloscope on ovarian cancer patient OV2295.** Haplotype-specific copy number profiles are compared for CNRein, SIGNALS, CHISEL and Alleloscope on $n = 617$ cells from ovarian cancer patient OV2295 [9]. Distances are calculated as in equation (67), with each bin phased to minimize errors between methods (as was done when comparing SIGNALS and CHISEL to ground truth copy numbers in Section B.3. Alleloscope is by far the most distant from other methods, with a distance over 1.35 to any other method while all other methods' predictions are within a distance of 0.265 of each other. This is due to the lack of whole genome duplication in Alleloscopes predictions.

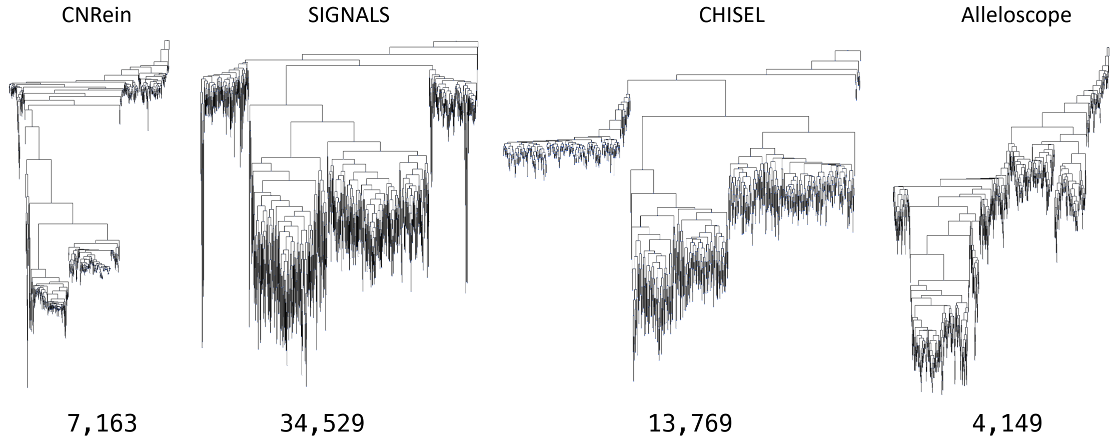

Figure S11: **Full trees of each method for ovarian cancer patient OV2295.** Full trees on the set of unique copy number profiles (clones) for CNRein, SIGNALS, CHISEL, and Alleloscope. These trees are scaled to the same height for plotting, however, the relative length of branches in each plot is proportional to the number of CNA events on the branch (estimated by the ZCNT distance). Parsimony values are labeled below each tree. Branches near the leaves are the shortest for CNRein and Alleloscope, the longest for SIGNALS, and have intermediate lengths for CHISEL. Inferring spurious CNAs results in CNAs that do not follow an evolutionary tree and consequently occur near leaves (possibly with homoplasy). Additionally, Alleloscope missing legitimate differences in whole genome duplication results in a very low parsimony score.

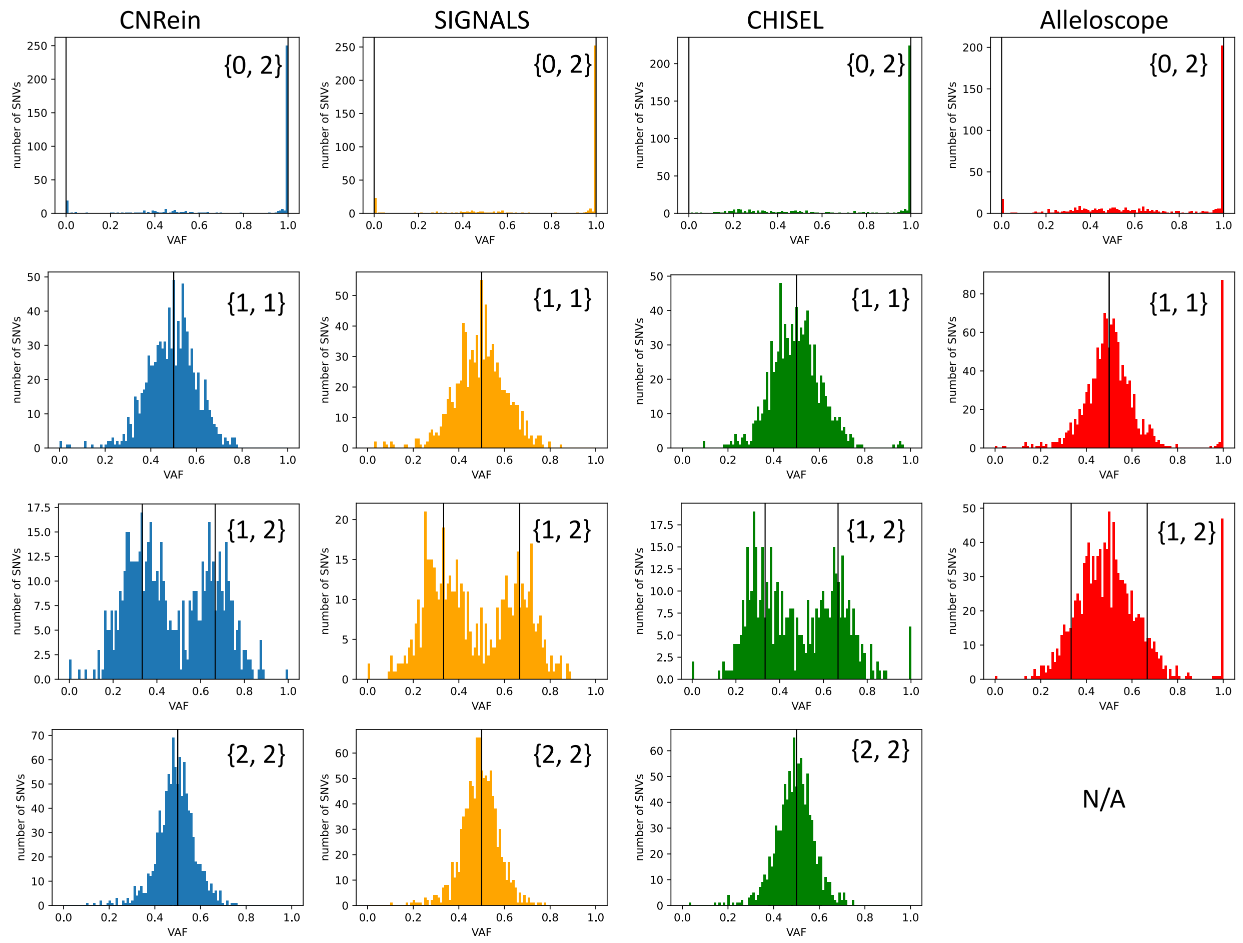

Figure S12: **VAF values for common copy numbers for ovarian cancer patient OV2295.** The common copy numbers $\{0,2\}$, $\{1,1\}$, and $\{1,2\}$ are shown for all CNRein, SIGNALS, CHISEL, and Alleloscope. The copy number $\{2,2\}$ is shown for CNRein, SIGNALS, and CHISEL, but not Alleloscope (due to the rarity of this copy number according to Alleloscope's predictions). With the exception of Alleloscope, all VAF plots have peaks around the theoretically expected values as indicated with black vertical lines.

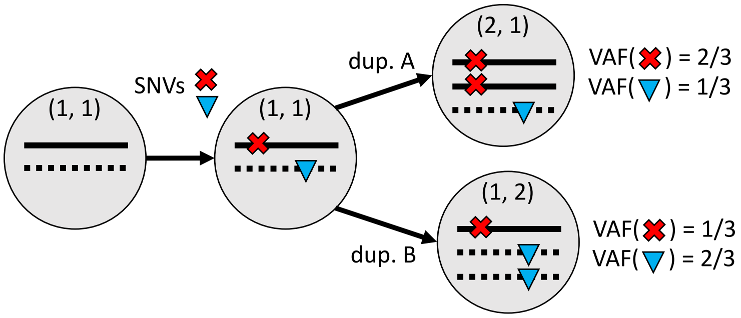

Figure S13: **A diagram of how allelic mirroring affects VAFs.** The impact of allelic mirroring on the VAF of truncal SNVs is shown. Specifically, the red SNV occurs on haplotype A (solid) and thus has an expected VAF of $1/3$ on copy number $(1, 2)$ and an expected VAF of $2/3$ on copy number $(2, 1)$. Similarly, the blue SNV occurs on haplotype B (dashed) and thus has an expected VAF of $2/3$ on copy number $(1, 2)$ and an expected VAF of $1/3$ on copy number $(2, 1)$.

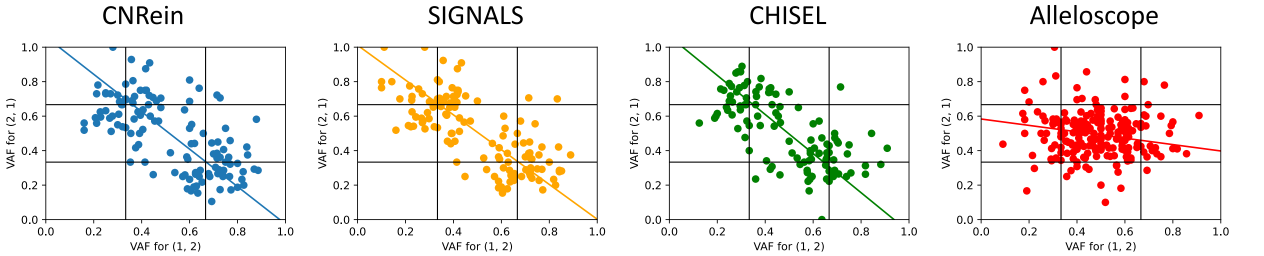

Figure S14: **VAFs demonstrating allelic mirroring for copy number $\{2, 1\}$ on ovarian cancer patient OV2295.** VAFs are shown for SNVs that occur on both copy number $(1, 2)$ and $(2, 1)$ due to allelic mirroring. A best fit line for each method is shown where orthogonal distance regression is used due to their being noise in both x and y variable measurements. A strong negative Pearson correlation between VAFs on $(1, 2)$ and $(2, 1)$ demonstrates SNV support of predicted allelic mirroring for CNRein ($r = -0.65$), SIGNALS ($r = -0.72$) and CHISEL ($r = -0.67$) but not Alleloscope ($r = -0.05$).

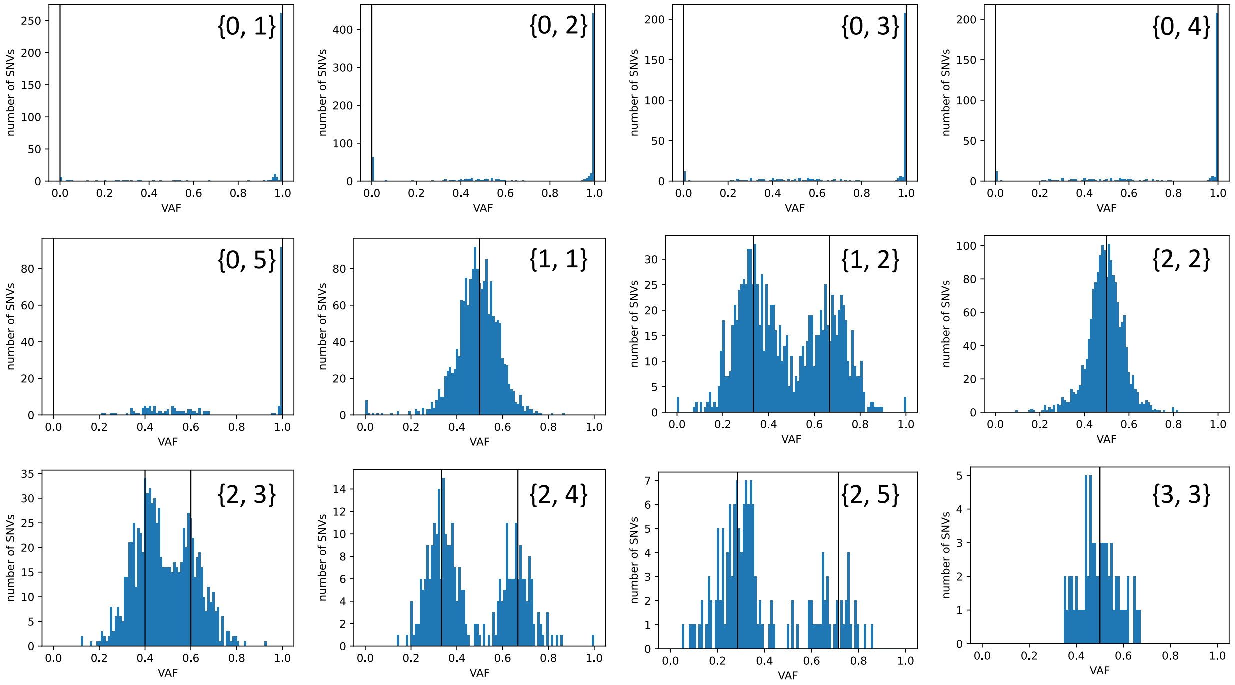

Figure S15: **VAFs of truncal SNVs overlapping with various copy numbers inferred by CNRein on ovarian cancer patient OV2295.** VAFs are shown for all the top-11 copy numbers with the most SNVs (or equivalently, all copy numbers with at least 140 SNVs occurring on that copy number), as well as the copy number $\{3, 3\}$ (which has 64 SNVs but is still very visually clear due to the simple unimodal VAF structure). The allele-specific copy number of each plot is indicated on the plot. Black vertical lines indicate the expected VAF of SNVs either occurring on the major or minor allele. On all plots, the VAFs are concentrated around these expected numbers.

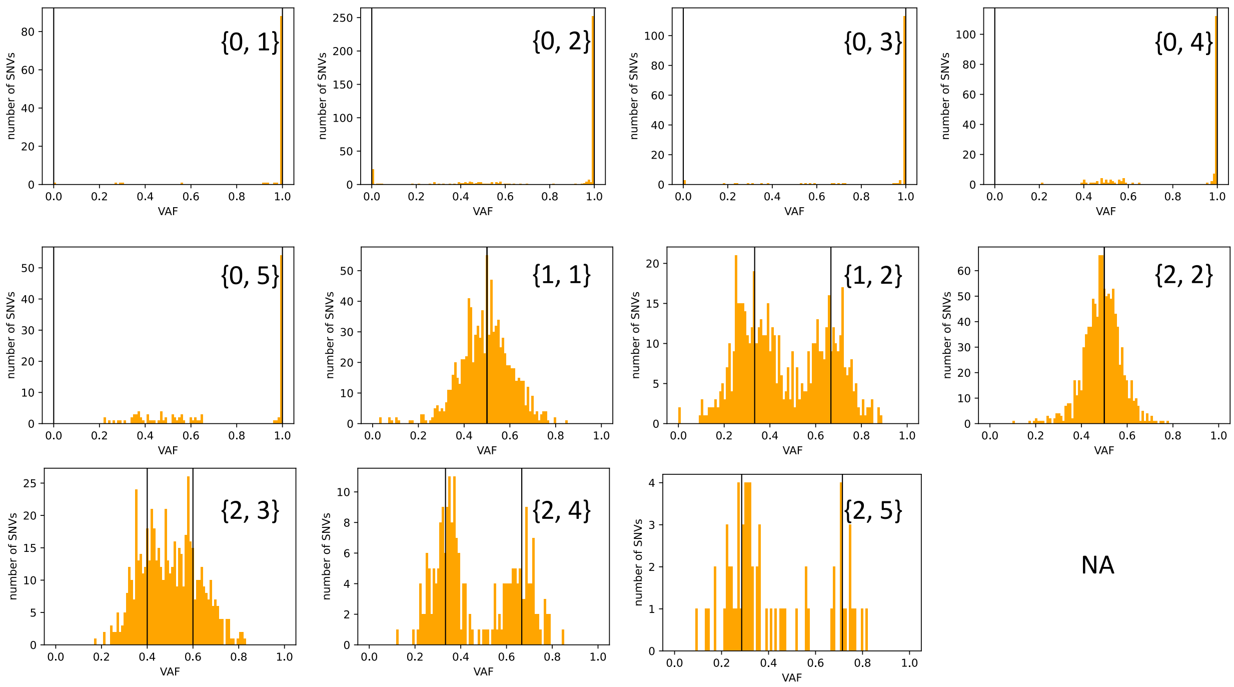

Figure S16: **VAFs of truncal SNVs overlapping with various copy numbers inferred by SIGNALS on ovarian cancer patient OV2295.** VAFs are shown for all the top-11 copy numbers with the most SNVs. The allele-specific copy number of each plot is indicated on the plot. Black vertical lines indicate the expected VAF of SNVs either occurring on the major or minor allele.

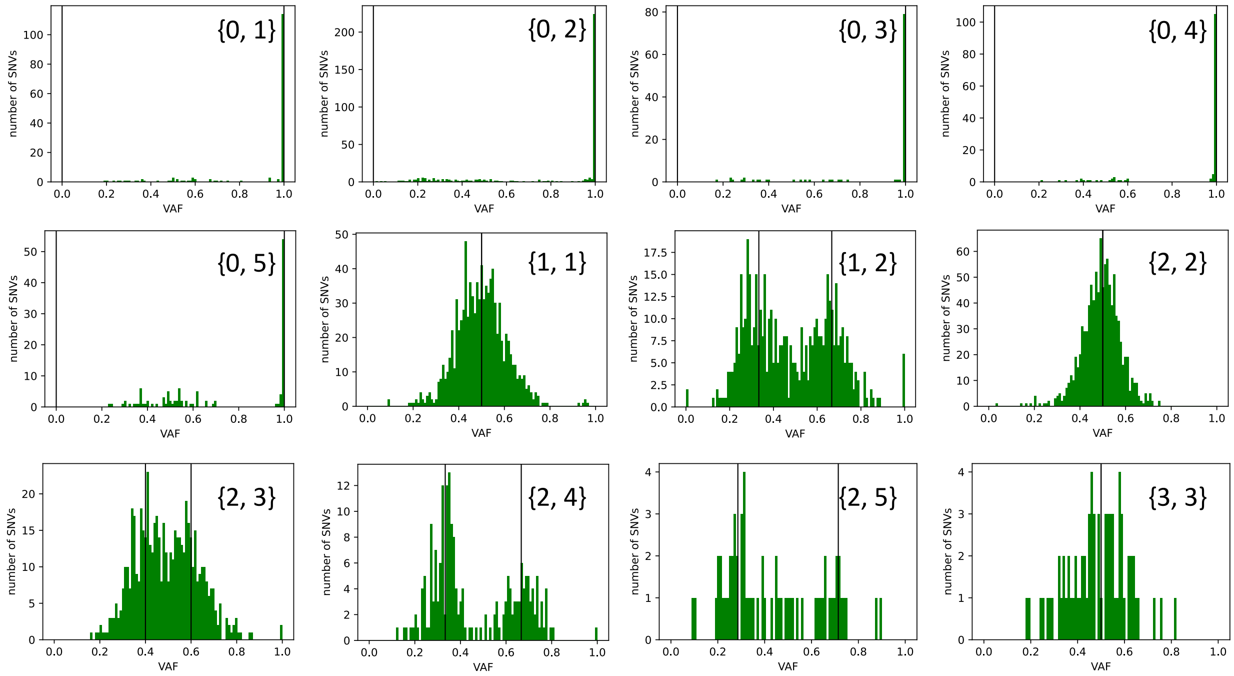

Figure S17: **VAFs of truncal SNVs overlapping with various copy numbers inferred by CHISEL on ovarian cancer patient OV2295.** VAFs are shown for all the top-11 copy numbers with the most SNVs as well as $\{3, 3\}$ as was done in Fig. S15. The allele-specific copy number of each plot is indicated on the plot. Black vertical lines indicate the expected VAF of SNVs either occurring on the major or minor allele.

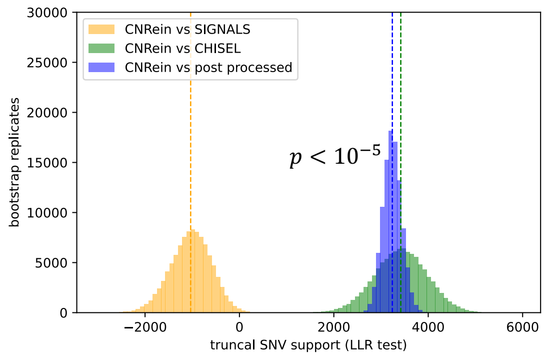

Figure S18: **Truncal SNV analysis of CNRein post processed to remove cell-specific copy number profiles on ovarian cancer patient OV2295.** We analyze the sensitivity of our orthogonal SNV-based analysis to the detection cell-specific copy number profiles. Specifically, we compare CNRein with a version of CNRein post-processed to have no cell-specific copy number profiles. Removing cell-specific copy number profiles caused CNRein to fit orthogonal SNV data worse (log likelihood ratio 3239.52, p value $< 10^{-5}$).

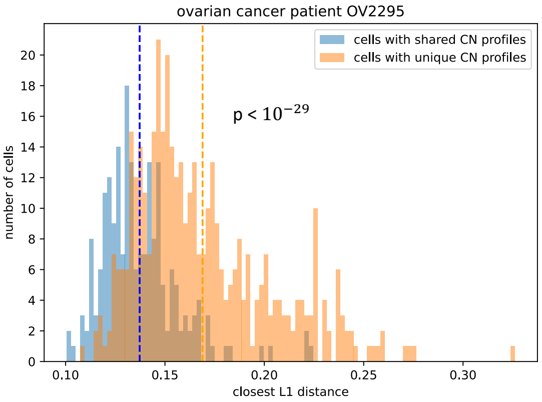

Figure S19: **Cells with predicted cell-specific profiles have more distinct read depths.** For each cell in ovarian cancer patient OV2295, we calculated the minimum L1 distance between read depths to any other cell (averaged across the genome). As expected, this minimum distance is smaller for cells with the same copy number profile as other cells (mean value 0.137) when compared to cells with cell-specific profiles (mean value 0.168). Mean values are indicated by dashed vertical lines. Applying the student's t-test gives a p value of $4.8 \cdot 10^{-30}$.

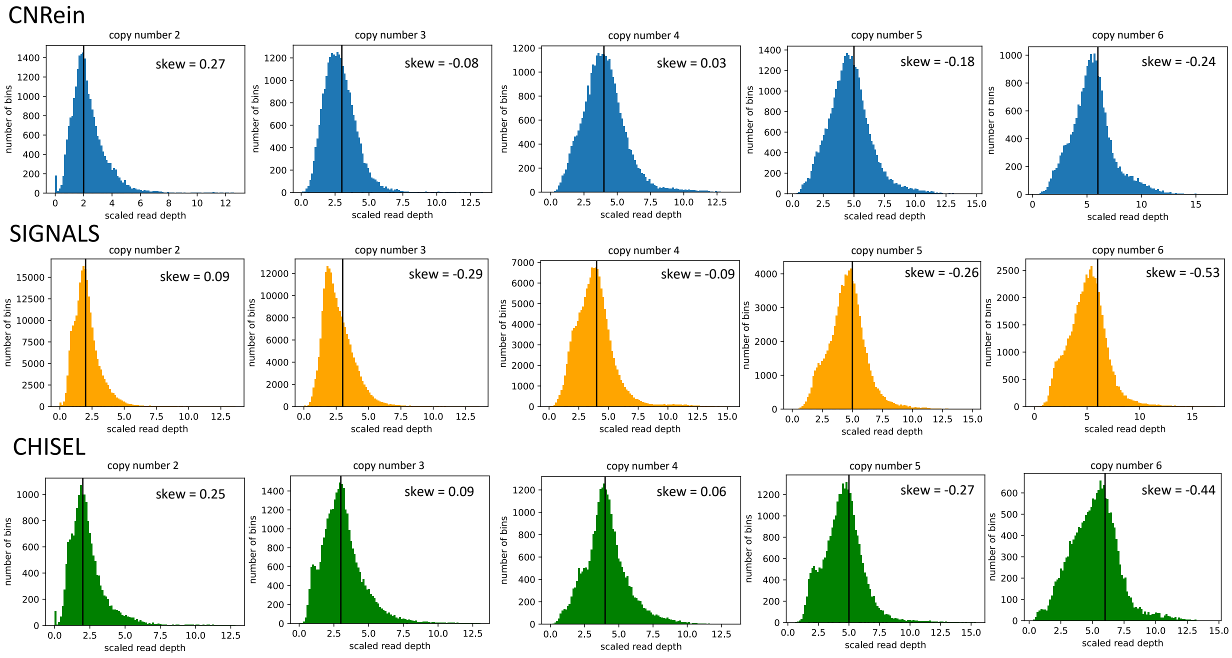

Figure S20: **Distributions of scaled read depth for cell-specific CNAs with different total copy numbers.** Bins contained within cell-specific CNAs are analyzed for CNRein, SIGNALS, and CHISEL. Each plot shows the distribution of scaled read depths for cell-specific CNAs of some fixed total copy number from 2 to 6 indicated by the black vertical line. Ideally, each distribution would be concentrated around the vertical line, however, this is not perfectly the case indicating some spurious predicted CNAs. For all copy numbers other than 2, CNRein achieves the lowest modified skewness value, indicating the least lopsided distribution.

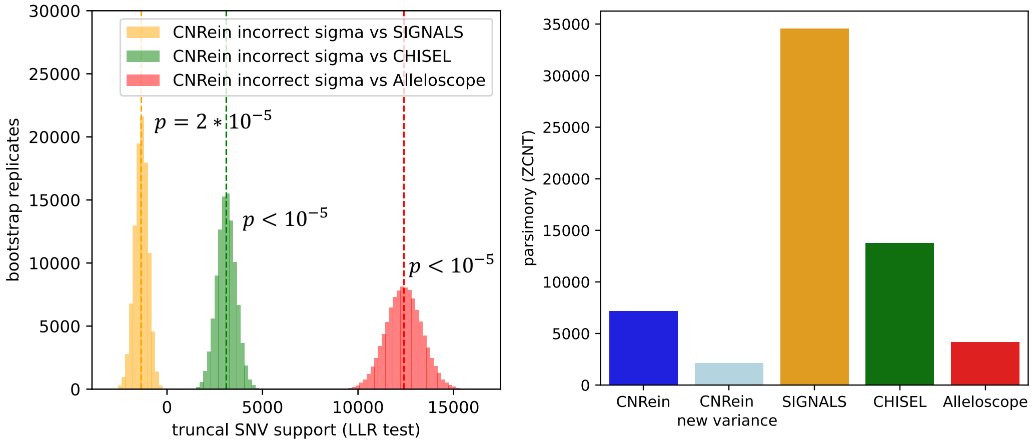

Figure S21: **CNRein with incorrect variances on ovarian cancer patient OV2295.** We test CNRein when the variances Σ^R and Σ^B are made artificially higher by a factor of 10. **a** SNV based analysis shows similar but slightly worse results as the original CNRein in terms of log liklihood ratios. **b** CNRein with increased variance estimates have a lower parsimony value of 2100 relative to the original value of 7163 due to the increased focus on finding a coherent solution across cells.

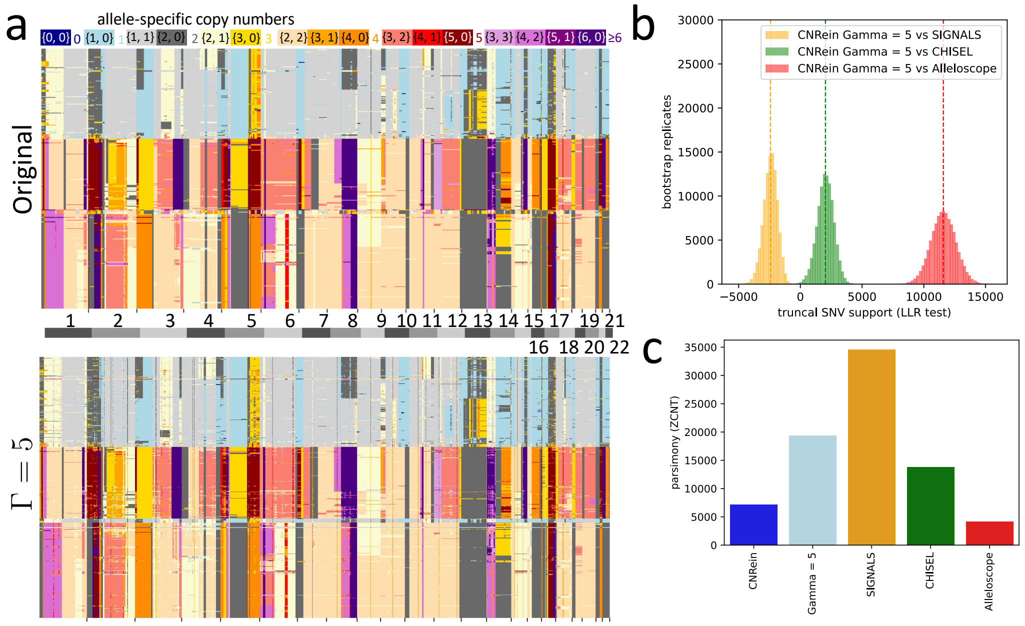

Figure S22: **CNRein predictions with modified segment sizes on ovarian cancer patient OV2295.** We produce CNRein predictions with $\Gamma = 5$ included for segmentation, producing smaller segments. **a** We compare the predicted copy number profiles with the modified segmentation profiles. **b** We apply SNV-based orthogonal validation to the modified version of CNRein. **c** We measure the parsimony of the modified CNRein predictions, finding that it increases the parsimony value.

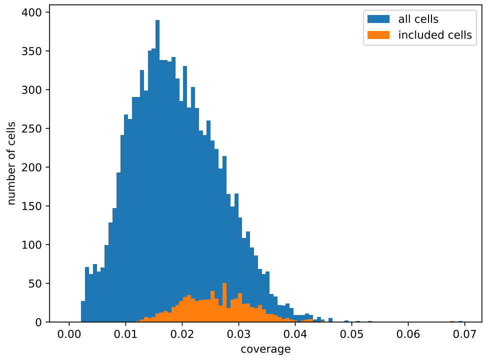

Figure S23: **The coverage of cells of breast cancer patient S0.** A histogram of the coverage of cells. In blue is the set of all 10,202 available cells. In orange are the $n = 785$ cells for which all methods were run on and comparisons were performed. Note that many of the excluded cells were normal (non-cancerous) cells, which will have a lower coverage due to the absence of amplifications and whole genome duplications.

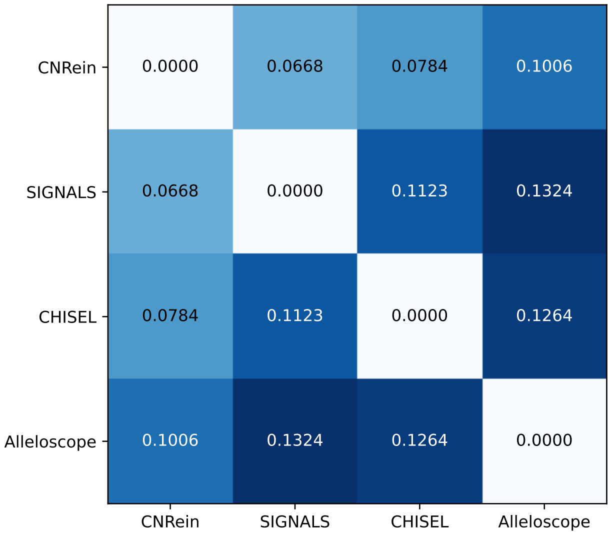

Figure S24: **L1 differences between copy number profiles predicted by CNRein, SIGNALS, CHISEL, and Alleloscope on breast cancer patient S0.** Haplotype-specific copy number profiles are compared for CNRein, SIGNALS, CHISEL, and Alleloscope on $n = 785$ cells from breast cancer patient S0. Distances are calculated as in equation (67), with each bin phased to minimize errors between methods (as was done when comparing SIGNALS and CHISEL to ground truth copy numbers in Section B.3. For all methods, the minimum distance alternative method prediction is CNRein, indicating that CNRein best fits the consensus of other methods.

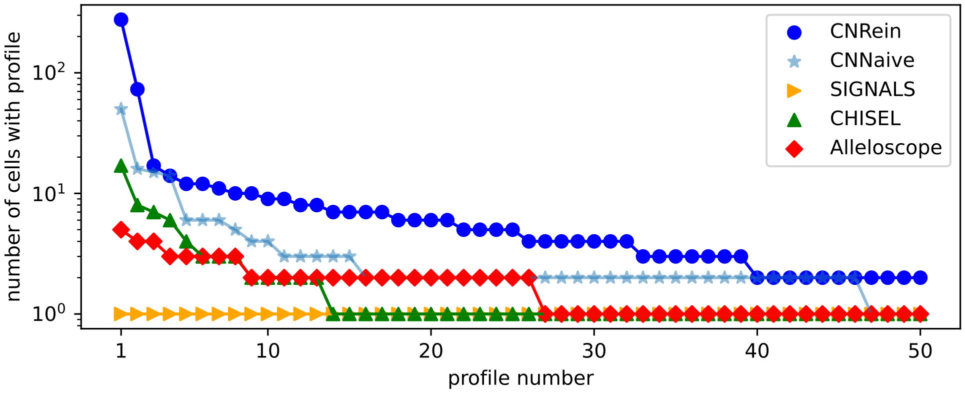

Figure S25: **The number of cells for CNNaive for common copy number profiles on breast cancer patient S0.** The number of cells for the top 50 most common copy number profiles on breast cancer patient S0 is shown for CNRein, CNNaive, SIGNALS, CHISEL, and Allelscope. Specifically, CNNaive is now included to show the specific advantage of CNRein's evolutionary model. The number of cells in the most common copy number profile is 276, 50, 1, 17, and 5 for CNRein, CNNaive, SIGNALS, CHISEL, and Alleloscope, respectively. The number of unique copy number profiles is 206, 628 785, 737, and 747 for CNRein, CNNaive, SIGNALS, CHISEL, and Alleloscope, respectively.

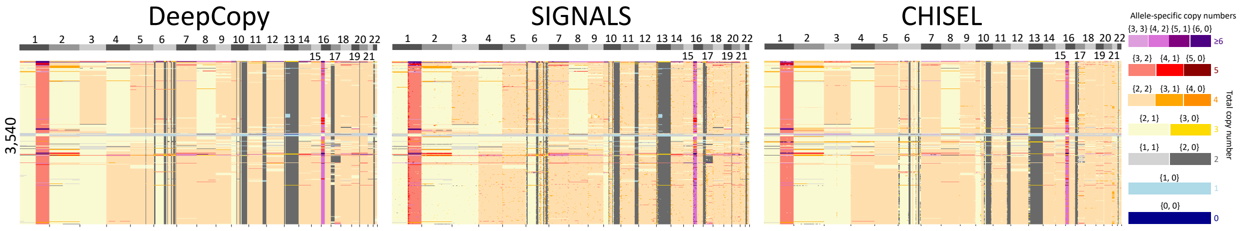

Figure S26: **A heatmap of allele-specific copy number predictions on** 3,540 **cells from breast cancer patient S0.** Here we show allele-specific copy number predictions on the full set of 3,540 cells for which CNRein, SIGNALS, and CHISEL have predicted profiles.

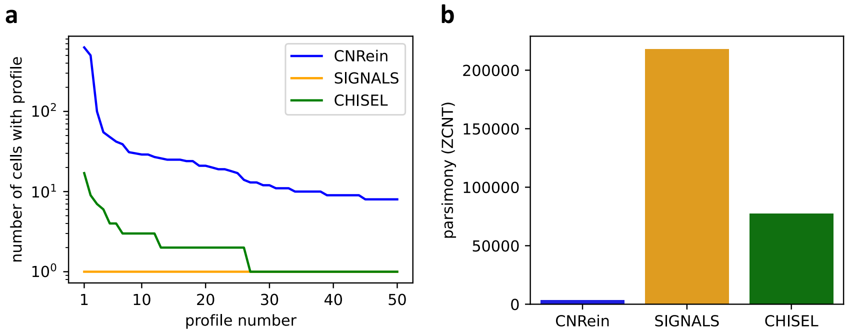

Figure S27: **Clone sizes and parsimony on on** 3,540 **cells from breast cancer patient S0. a** A comparison of clone sizes for CNRein, CHISEL, and SIGNALS. The most common unique copy number profiles have 629, 1, and 17 cells for CNRein, CHISEL, and SIGNALS respectively. **b** A comparison of parsimony scores for CNRein, CHISEL, and SIGNALS, showing parsimony scores of 3,687, 218,145, and 77,411, respectively.

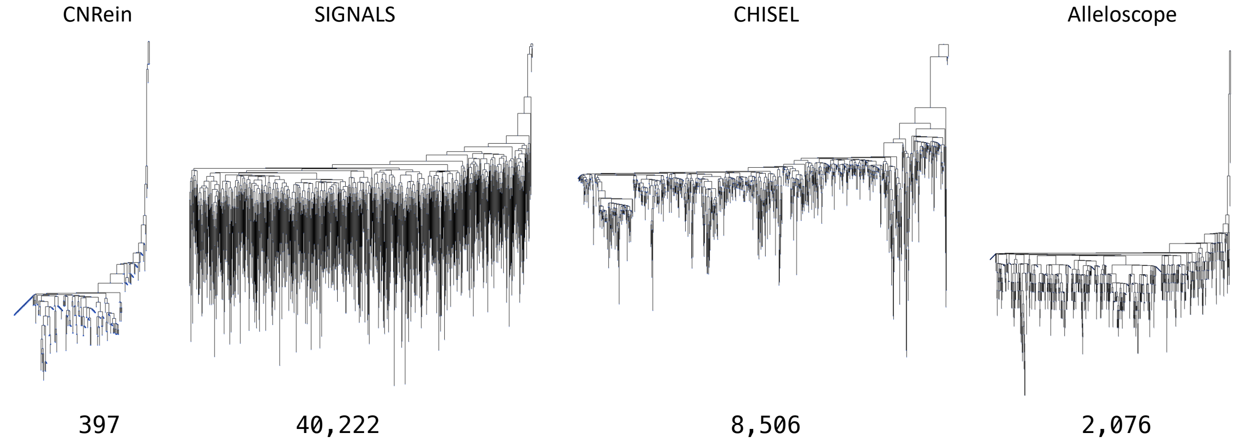

Figure S28: **Full trees of each method on breast cancer patient S0.** Full trees on the set of unique copy number profiles (clones) for CNRein, SIGNALS, CHISEL, and CNNaive. All trees are scaled to the same height for plotting, however, the relative length of branches in each plot is proportional to the number of CNA events on the branch (estimated by the ZCNT distance). Parsimony values are labeled next to each tree. Branches near the leaves are the shortest for CNRein, longer for CHISEL and Alleloscope, and extremely long for SIGNALS. Inferring spurious CNAs results in CNAs that do not follow an evolutionary tree and consequently occur near leaves (possibly with homoplasy), resulting in very long branches near the leaves.

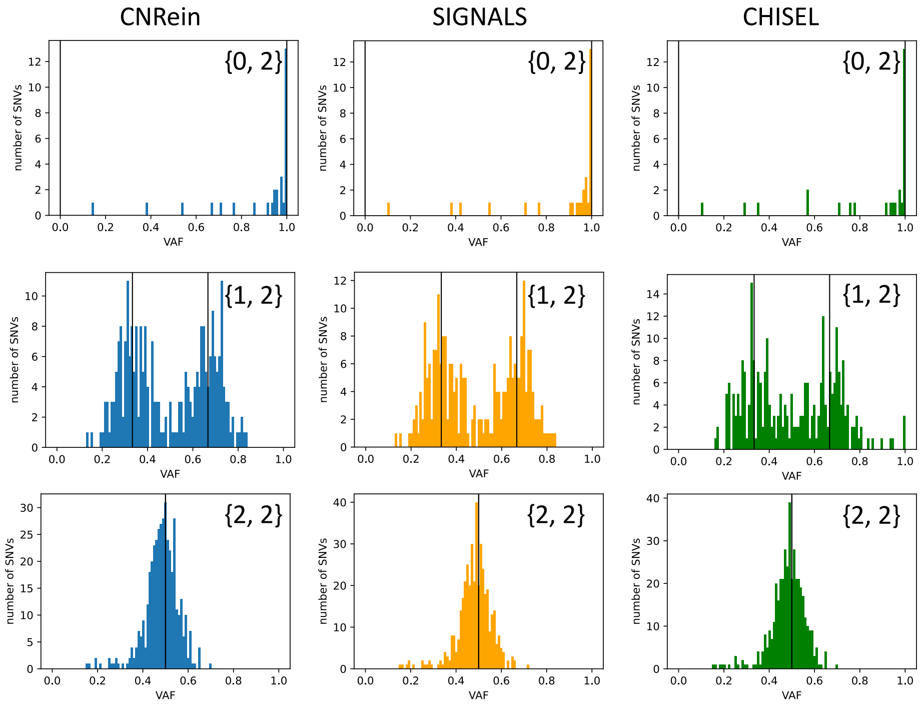

Figure S29: **VAFs of truncal SNVs overlapping with three common copy numbers for CNRein SIGNALS and CHISEL on breast cancer patient S0.** VAFs are shown for the top-3 copy numbers with the most SNVs (or equivalently, all copy numbers with at least 28 SNVs occurring on that copy number for CNRein, CHISEL, and SIGNALS). The allele-specific copy number of each plot is indicated on the plot. Black vertical lines indicate the expected VAF of SNVs either occurring on the major or minor allele. The VAFs are concentrated around these expected numbers.

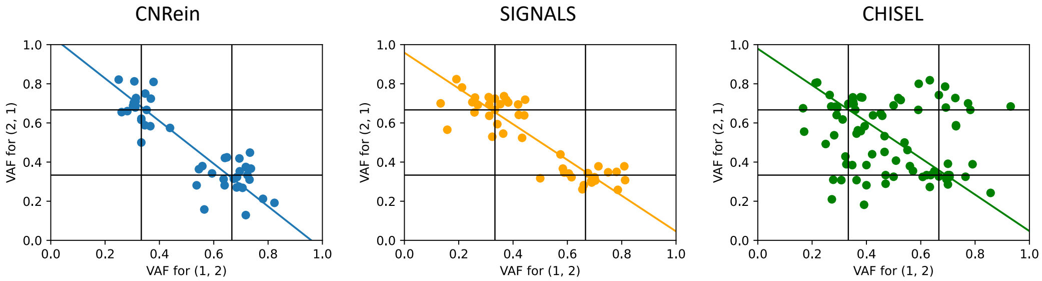

Figure S30: **VAFs demonstrating allelic mirroring for copy number $\{2, 1\}$ on breast cancer patient S0.** VAFs are shown for SNVs that occur on both copy number $(1, 2)$ and $(2, 1)$ due to allelic mirroring. A best fit line for each method is shown where orthogonal distance regression is used due to their being noise in both x and y variable measurements. A strong negative Pearson correlation between VAFs on $(1, 2)$ and $(2, 1)$ demonstrates SNV support of predicted allelic mirroring for CNRein ($r = -0.89$) and SIGNALS ($r = -0.89$), but not CHISEL ($r = -0.20$).

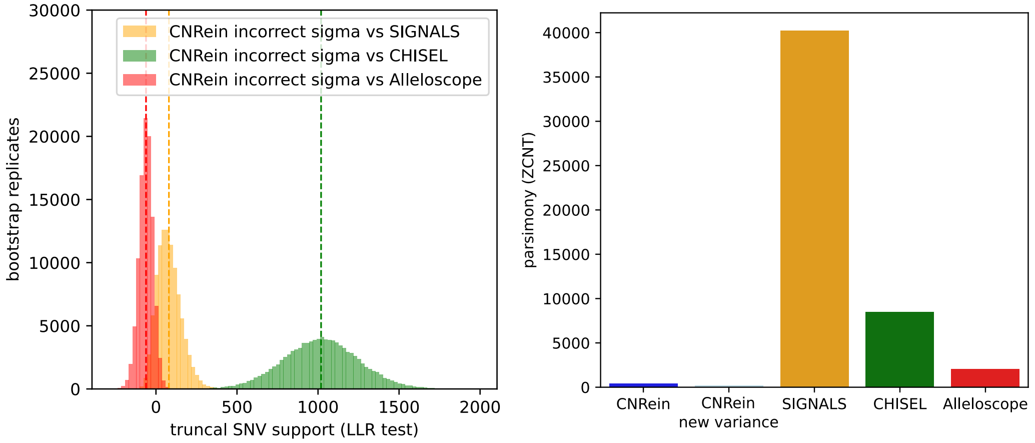

Figure S31: **CNRein with incorrect variances on breast cancer patient S0.** We test CNRein when the variances Σ^R and Σ^B are made artificially higher by a factor of 10. **a** SNV-based analysis shows similar but slightly worse results as the original CNRein in terms of log likelihood ratios. **b** CNRein with increased variance estimates have a lower parsimony value of 209 relative to the original value of 397 due to the increased focus on finding a coherent solution across cells.

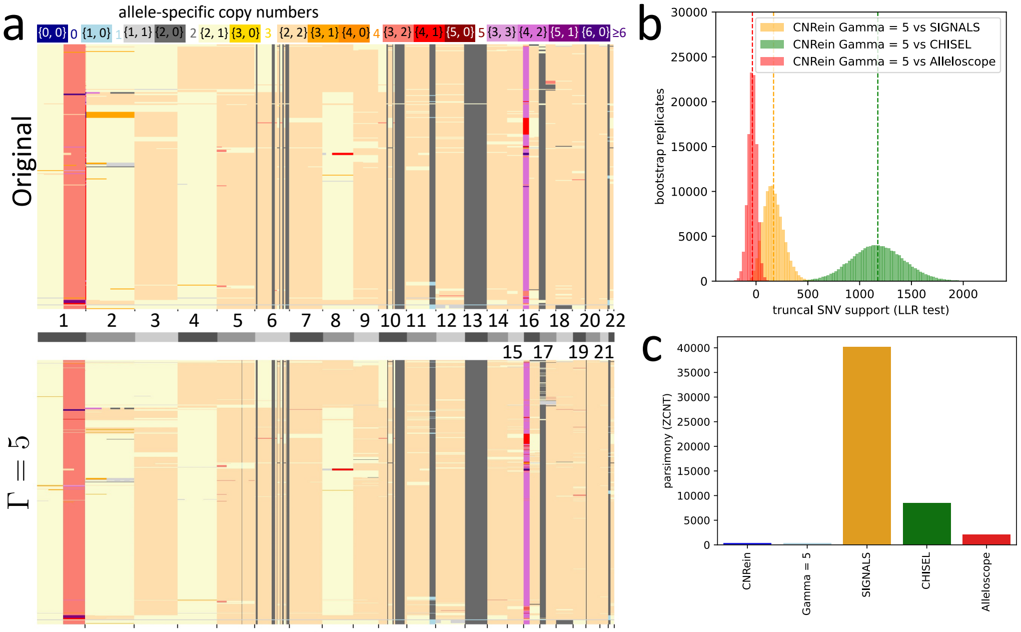

Figure S32: **CNRein predictions with modified segment sizes on ovarian cancer patient OV2295.** We produce CNRein predictions with $\Gamma = 5$ included for segmentation, producing smaller segments. **a** We compare the predicted copy number profiles with the modified segmentation profiles. **b** We apply SNV-based orthogonal validation to the modified version of CNRein. **c** We measure the parsimony of the modified CNRein predictions, finding that it increases the parsimony value.

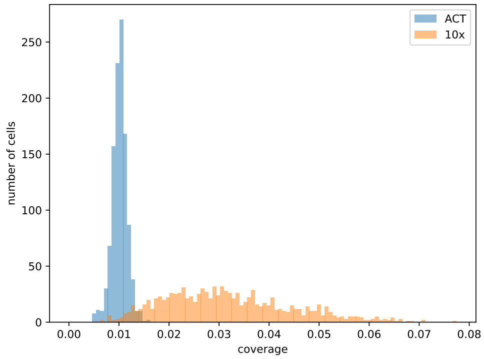

Figure S33: **The coverage of cells of breast cancer patient TN3.** The coverage of breast cancer patient TN3 cells, with blue representing cells sequenced with ACT technology and orange representing cells sequenced with 10x technology.

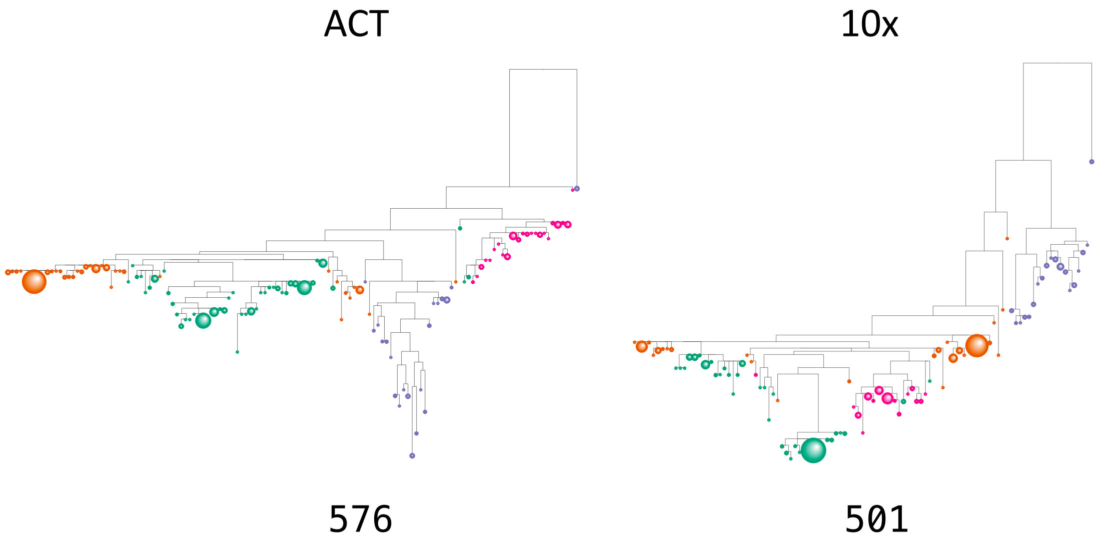

Figure S34: **Phylogenies for breast cancer patient TN3 for both sequencing technologies.** Phylogenies are shown for patient TN3, with a node for each unique copy number profile. The sizes (area) of the nodes are proportional to the number of cells with that profile, and the color of the nodes matches the UMAP clusters (shown in Main text: Fig. 5). As shown below the trees, the parsimony score is 576 on the cells sequenced with ACT technology and the parsimony is 501 on the trees sequenced with 10x technology. Note that parsimony scores can be strongly affected by focal CNAs on small numbers of cells, resulting in different parsimony values despite their being highly similar predicted copy number profiles.

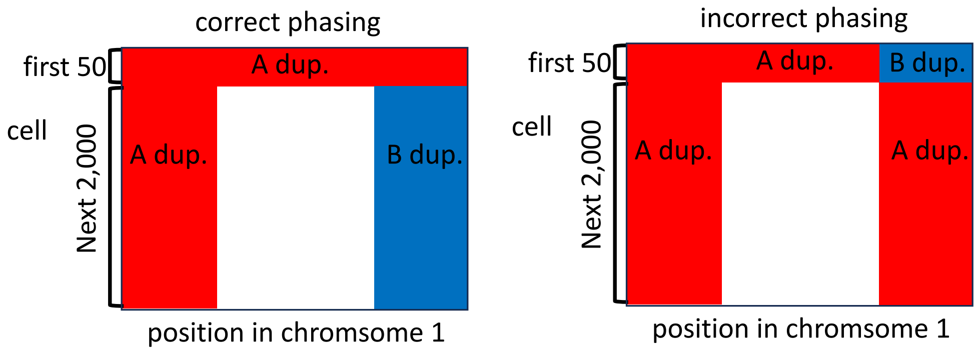

Figure S35: **An example of a chromosome requiring our adjusted phasing algorithm.** A hypothetical copy number profile for chromosome 1 is shown. The first 50 cells have duplication of haplotype A. The remaining 2000 cells have duplication of haplotype A in the first quarter of the chromosome and duplication of haplotype B in the last quarter of the chromosome. The simplified version of our phasing algorithm would primarily utilize the last 2000 cells for phasing and incorrectly phase the last quarter of the chromosome relative to the first quarter. However, our modified phasing algorithm considers multiple sets of cells for phasing to be able to phase this correctly.
