# Supplementary material for: CNRein: an evolution-aware deep reinforcement learning algorithm for single-cell DNA copy number calling
Source: Genome Biol. 2025 Apr 7;26:87. doi: 10.1186/s13059-025-03553-2 (PMC11974095; doi:10.1186/s13059-025-03553-2)
Supplement: Supplementary file 2 — Additional file 2. Review history. [file 13059_2025_3553_MOESM2_ESM.docx]

Review History

**First round of review**

**Reviewer 1**

**Were you able to assess all statistics in the manuscript, including the appropriateness of statistical tests used?**

Yes: Statistical tests seem valid.

**Were you able to directly test the methods?**

No

**Comments to author:**

The authors describe DeepCopy, a method for inferring copy number states from single cell whole genome sequence data. The method uses an evolutionary model and a reinforcement learning algorithm to infer copy number states jointly across a population of related tumor cells. The method is interesting and will almost certainly be useful in single cell cancer genomics projects. The benchmarking experiments are well constructed. My main concern is that DeepCopy may be insensitive to cell specific CNA. The profiles produced by DeepCopy look cleaner than the other methods, but is that at the expense of true cell specific events? Unfortunately I dont believe the current benchmarking results clearly evaluate DeepCopy's sensitivity to such events.

Major:

1. Why are larger clones more desirable in output on real data? Do you believe that many of the cells sequenced have truly identical copy-number profiles, or is it merely the case that the per-cell coverage and data quality limit recovery of cell-specific events? I think the authors should comment on this in the introduction and/or discussion, because while indeed inferring fewer larger clones is more parsimonious, it is not obvious that it is a desirable goal or ultimately a more accurate description of the underlying biology.

2. Does DeepCopy perform any correction for errors in the reference-based haplotype phasing within bins? If not, what enables the subsequent steps to be robust to switch errors? Using SHAPEIT with the 1000 Genomes reference panel, SNPs within 100kb of each other can be phased incorrectly up to ~10% of the time (Fig. 3A from Choi et al., PLoS Genetics 2018).

3. The SVD-based haplotype phasing algorithm is very interesting but it is not clear to me why a rank-10 matrix approximation is necessary, what information it encodes, and overall how the algorithm accomplishes the goal of aligning phasing blocks across the chromosome. Are the "groups" of 100 haplotype blocks overlapping? Why an exponential moving average?

4. What evidence is there that the fewer copy-number profiles inferred by DeepCopy represent a better fit to the data? Can you contrast the cell-specific events inferred by DeepCopy (i.e., in clones of size 1) with those inferred by other methods to show that DeepCopy only combines cells into clones where there truly is insufficient evidence for cell-specific events?

5. Previous analysis of OV2295 (see Funnell 2022, Extended Data Fig. 3), showed scattered WGD in the non-WGD sample. Does DeepCopy predict the same? If not, why not?

6. How does the use of ZCNT interact with clonal or subclonal WGD in simulations and real data?

7. The DeepCopy profiles for OV2295 look more smoothed over than those for chisel or especially signals (ignoring allelescope which seems to be having trouble). Can the authors be sure those cell specific CNVs are not real? Showing the raw read depth alongside the inferred copy number state will help show whether the cell specific events are spurious.

8. The claim that "DeepCopy avoids spurious CNAs producing a more parsimonious solution than SIGNALS or CHISEL" is not supported by the evidence. Using parsimony cannot assess whether DeepCopy smooths over cell specific events, or whether signals or chisel predicts spurious CNA". Instead, an appeal to each method's fit to raw read depth signal may help here.

9. One of the main methods by which the authors evaluate copy number, and indeed a major contribution of the paper, is to construct a tree and then calculate a parsimony score. If the copy number is overly smoothed, removing cell specific CNAs, building a phylogeny may be more difficult. A worse parsimony score may thus be due to the increased difficulty in tree building rather than inaccuracies in copy number estimation. In other words, the parsimony evaluation method may bias against calling a higher amount of true cell specific CNA. Can the authors provide evidence this is not the case? Applying tree building plus parsimony scoring to true copy number from simulations with varying amount of cell specific CNAs would help rule out this phenomenon or at least identify the degree to which it is a problem.

10. The authors claim that "DeepCopy avoids spurious CNAs by producing a far more parsimonious solution" (line 240). This does not seem to be well supported by their current benchmarking. Specifically, it is not clear that using SNV likelihood based evaluation, a great idea in general, will be sufficiently sensitive to discern between methods that predict spurious cell specific CNAs, identify true cell specific CNAs, or smooth over true cell specific CNAs.

Minor:

1. The input format requires cells in the BAM to be labeled by read group instead of CB tag. Labeling cells by CB tag is preferable. Droplet based methods produce too many barcodes to be stored as one barcode per read group. Also a merged bam with many read groups is incompatible with pseudobulk SNV and breakpoint calling. Please add support for CB tag based BAM input.

2. In the segmentation algorithm, did you experiment with setting Gamma smaller than 10 to recover smaller copy-number segments? How are the 6 evidence values combined for each candidate breakpoint?

3. In what sense is the relaxation to a Gaussian distribution using the central limit theorem valid in single-cell sequencing data when each bin may have fewer than 50 reads, with much fewer reads covering SNPs within the bin? Does this refer to a relaxation at the segment level?

4. The choice of name DeepCopy may not be ideal, as it is also a commonly used computer science term which could make the method much harder to find. Also, there is already a method DeepCNA (Liu et al., BMC Genomics 2024) for inferring CNAs from scDNA-seq, which could be confusing.

5. Some sections, especially methods, are complex without adequate explanation. For instance "B.2 Detection of small CNAs" relies on formulas almost exclusively and a few sentences would help the reader understand what the formulas mean.

6. "vast majority of truncal SNVs seem to precede their coinciding CNAs" -> I would add CNAs and WGD here if that in fact is true.

7. Line 213 " small CNAs in SIGNALS predictions likely due to noise", be specific about these CNAs. what are their sizes and cell specificity?

Typos:

Supplement page 31 line 745 "moving exponential moving"

Supplement page 36 line 33 "minimizing the blow"

**Reviewer 2**

**Were you able to assess all statistics in the manuscript, including the appropriateness of statistical tests used?** Yes

**Were you able to directly test the methods?** No

**Comments to author:**

General comments

This is a very interesting article describing a significant effort regarding allele-specific copy number calling for single cancer cells or subclones. Unfortunately, the article has several severe problems which are intertwined. The first two are concerned with the evaluation of the results and comparisons with other methods, which is related to the question about the purpose of the method: is the purpose copy number calling for individual cells or cancer subclones? After having read the submission, it is impossible to know if the proposed method is better than earlier methods. The third and fourth severe problems are that the authors have developed almost everything in the article independently. So, previously developed standard methods are not used, and many details are provided that make a better organization desirable.

Copy number calling for cancer clones regards clustering single cells into sub-clones and assigning all single cells to the same copy number profile in one sub-clone. When constructing a single tree from the copy number profiles, one can expect to get a lower parsimony score if each cell has been assigned a unique copy number profile. So the comparison between Deepcopy due to its regular ratio, induced by the RL framework, and copy number calls for individual cells made by the other method, for instance, Chisel, is unfair. Moreover, Chisel's original publication describes it as a method in which one of the final steps constitutes a clustering of the single cell copy number profiles and trees constructed in the publication or trees on sub-clones. In addition to these problems, there are other methods that I've been using over the last couple of years, such as Conet, Sitka, and VicTree. One course argued that they are not allele-specific, but they may still perform better than the allele-specific method presented here! So, a comparison is necessary.

There are, in particular, three lines of research that the submission would have benefited from taking advantage of. The first is the so-called G flow nets (GFNs) described in several papers, mainly from Bengio's group. GFNs have been used to construct various types of bio sequences. The RL framework used in this article seems to be a less mature version of GFNs. One of the sampling steps used to find a normalizing constant could be better handled using GFNs.
Moreover, it would make the method presentation shorter and more standard. Second, a technique called CNAsim was published by Weiner and Bansal, which can be used to simulate copy number evolution in cancer cells. Using this framework would shorten the presentation and facilitate a better comparison with other methods. For the site, the paper is. "A zero-agnostic model for copy number evolution in cancer." That paper describes a parsimony approach that could've been exploited in the submission rather than, as it's now done, using a few results from there to construct a new parsimony approach. Again, using the previously published method would have improved the submission's presentation and facilitated a better comparison with other methods.

Finally, the submission requires a better presentation and organization. The multilayered organization, that is, with references to the method section, which contains references to supplements, etc., makes the paper very hard to read. Consequently, more material has to be presented in the same section. There's no reason to first introduce the measure probability and then later on actually define it., etc.

Specific comments

Evolution-Aware Deep Reinforcement Learning for Single-Cell DNA Copy Number Calling
Draft-- M

" evolution- 11 ary constraint"
10: You need a better intuitive description or what your evolutionary constraints are. It shouldn't go into the abstract but the introduction.

" A key limitation of these data is that 38 we do not measure read depth and BAF in individual clones but rather obtain composite measurements of 39 millions of cells in the bulk sampl"
37: This is basically saying that the limit of bulk data is that it's bulk data.

"verview of DeepCopy."
Fig 1: This has hardly any information.

" haplotype-specific copy number profiles P "
Fig 1d: For clones or cells? This should be made clear as early as possible.

" copy-neutral loss of heterozygosity and mirrored-subclonal 60 CNA"
59: I guess that this means that different alleles occur with number two in different cells. Please formulate this carefully.

" have not "
60: Do not take

" evolutionary process "
66: You need to supply more details about the model.

" on simulated data, "
71: What model is used for the simulation? Again I suggest CNAsim.

" final copy number estimation via deep reinforcement learning. "
84: GFNs should be discussed in the intro.

" perform bias correction and segmentation."
89: GC bias correction, right? What do you mean by segmentation? And how is it performed and used?

"uses low-noise regions to estimate the cell-specific scaling factor"
91 How and where is this described?

" based on a 96 generative model for copy number profiles that sequentially adds CNAs to clones starting from the normal 97 clone"
95: So paths are generated rather than trees? You need to explain this to the reader.

" In contrast to existing methods, "
95: Again, this is incorrect there are other evolutionary aware methods.

" views "
97: Less anthropomorphisms please

"r a single simulation instance with a whole genome duplication."
Fig 2. where in the tree is the WGD?

"A small amount of jitter was added to predictions with exactly 1,000 unique copy number profiles for clarity"
Fig 2 d: Jitter. Please explain.

" a truncal "
104 Define truncal and why only truncal?

"starting with either a normal clone or a whole genome duplicated clone,"
105: Why would a WGD happen before all other events? Why does it produce interesting data?

"CNAs to existing clones,"

" accuracy as the average 114 proportion of bins across all cells for which the predicted allele-specific copy number matches the true "

" For the shown simulation instance "
115: In fig 2a

" Note that high parsimony values may be re- 138 flective of homoplasy, i.e., identical CNAs occurring independently at many locations on the tree, due to a 139 lack of evolutionary structure in the predictions even if the predictions do not look very visually noisy as is 140 the case for CHISEL's prediction."
137: Hard to parse.

" We note that Alleloscope achieved an even lower parsimony 173 score of 3,651, however, we believe this is due to Alleloscope incorrectly predicting a lack of WGD"
173: So, final parsimony is not a great measure of success.

"Alleloscope did not predict WGD for any cell."
Fig 3: what does predicting WGD mean?

" predictions are far noisie "
Fig 3 b: How are the cells ordered? if it is based on a clustering based on the deepcopy copy number calls, then others may look more noisy. Anyway, noisiness is a so subjective term that it cannot be used unless turned into something quantifiable.

" are likely truncal, i.e."
176: The term truncal is used earlier.

" likely "

" must have a variant allele frequency (VAF) on those cells equal to some integer multiple of 1/(XA +XB 179 )"
179: This is true not only for truncal and a stronger statement can be made. What are you assuming regarding the order between the first duplication in the genomic segment of the SNV and the introduction of the SNV? In S8 The SNV is present before the duplication. In fact you make the stronger assumption below. Consequently, right here the assumption of truncal SNV is unnecessary. Moreover, the text should be better organized.

"each truncal SNV's "
191: How do you determine whether an SNV is truncal?

" avoids spurious CNAs producing a more parsimonious solution than SIGNALS or 201 CHISEL while retaining a high accuracy towards legitimate CNAs "
200: This core argument is not convincing.

" a slightly worse but not significant fit than Alleloscope (LLR −35.24 and p = 0.205). VAF 235 values are plotted in Fig. S17, showing only minor differences between methods. Additionally in Fig. S18, 236 we show that DeepCopy, SIGNALS, and CHISEL predict allelic mirroring on copy number {2, 1}, but this 237 prediction is supported by orthogonal SNV data only for DeepCopy and SIGNAL"
234: The SNV based test can only distinguish between certain pairs of copy number configurations.

"ons are far noisier "
Figure 4: Noise is not well fine

" we have shown that DeepCopy avoids spurious CNAs by producing a far more parsimo- 240 nious solution with fewer unique copy number profiles than "
239: This has to be rephrased

" DeepCopy models 265 the probabilities of trajectories starting on the normal cell and ending on some copy number profile"
264: This should be explained much much earlier

" more realistic clone sizes than existing methods"
269: This ambiguity concerning whether the methods are single cell number calling or clonal cop number, must be straightened out

"NPs"
312: use both the terms SNP and SNV. If you define them in the same way, use only one and provide the definition. Otherwise, give both definitions.

"This then produces the B-allele frequencies (BAFs) 314 B = [B 1,...,B N ]⊤ ∈ [0, 1]N × L , which measure the proportion of reads coming from the second haplo-315 type (typically set to be the minor haplotype), i.e., B s,i ≈ P(1) s,i / (P(1) s,i + P(2) s,i )"
314: This is in conflict with the above definition that has one of P^(1)_i and P^(2)_i has the paternal, i.e., uses 1 or 2 to indicate paternal.

"Pr(B s | ΣB s ,P)"
326: why isn't this conditioned by R?

"Supplementary Section A.2.1."
328 This overly distributed organization hinders the reader's understanding. Please define the distributions here.

" we define Pr(P | θ) 332 as the probability of the copy number profile P according to a model with tumor-specific parameters θ "
331: Give an intuitive explanation of the parameter theta

"P∈P"
336 (2): Why sum over P when you use argmax in (4)?

" counts for K bins of size ℓbin "
348: Before there was a discussion about segmenting now the segments seem to be fixed

" The genome is then split into L segments consisting of many 100kb bins, as 354 described in Supplementary Section A.1.3"
353: Each consist of many bins? Why is this done like this? Why don't you just work with the bins?

"eural network."
Fig 6 d: What is the input?

"hese"
Fig 6 d: Unclear reference.

"he set ̃ P of copy number profiles returned by NaiveCopy is used to help guide 362 DeepCopy's optimization. Fig. 6a-b shows a diagram of this pipeline."
362: It doesn't provide a lot of insight.

"ach cancer is trained on independently,"
367: Rephrase.

"This process repeats unit terminating"
375: Spelling.

"e define the reward function as 411 r(G) = NX s=1 Pr(R s,B s | θ)− 1 Pr(R s,B s | G)."
411: This resembles Gflow nets (GFNs). Using the developed GFN machinery, this paper could be made much more readable and elegant.

"To do so, rather 427 than looking at segments, we evaluated the K bins with a fixed size of 100kb."
427: explain

"a,b,a′,b′)"
439 (10): Here, you don't seem to keep track of maternal and paternal.

"Finally, parsimony values are calculated with edge 439 lengths determined by the ZCNT distance."
439: But the standard parsimony score does not depend on edge lengths.

"amplification on one allele"
693: understandable but probably not correct terminology

"The probability of observing values of CA s,η,CB s,η assuming there is no imbalance comes 700 from a binomial distribution which can be approximated by a Gaussian. Specifically, CA s,η − CB s,η has a mean 701 of zero, and a variance equal to the total number of counts CA s,η + CB s,η"
700: These probabilities have not been introduced yet, so a better organization is desirable. Furthermore, when introduced, the definitions are slightly different. Finally, I didn't get this variance, please explain.

"For any two haplotype blocks η and η′ in the same chromosome, we want to phase the "
710 This depends on the above assumption.

"Additional phasing algorithm details:"
728: This section must be better explained.

"2ΣR s,i"
878: The denominator must be explained. The total copy number is often used here.

"Pr(R s,B s | θ)− 1"
1003 (57): GFNs would most likely assist with estimating the normalizing factor.

"as defined in Supplementary Section A.3.8."
1008: This layer-after-layer organization makes reading and understanding hard.

"Efficient generative sequences"
1068: The content of A.38 can be expressed more efficiently and readable.


"such that the distance from P to P′ is 1135 equal to the distance from P′ to P, i.e., ZCNT(P,P′) = ZCNT(P′,P)"
1135: inefficient description.

"ZCNT(P(1) ,Q(1) ) + ZCNT(P(2) ,Q(2) )."
1137 (69): This requires keeping track of maternal and paternal. Do all methods do that?

"Once all internal node copy number profiles are determined, 1147 we can calculate the parsimony score of the tree by again applying ZCNT distances. Specifically, the length 1148 of each edge is equal to the ZCNT distance, as defined in equation (69), between the copy number profiles 1149 of the nodes connected by the edge. Then, the parsimony score is the sum of distances on all edges."
1147: This is an entirely reasonable approach. Still, it is also not standard, and it is hard to judge how well it fits the CN profile reconstruction methods.

"As discussed in the main text, and shown in Supplementary Section B.1, the 1182 majority of truncal SNVs are of the first type in our data"
1182: But will the fraction of the second type influence your results?

"max(Pr( v, r | X (1) , X (2) , A-allele), Pr(v, r | X (1) , X (2) , B-allele))."
1194: This means that you don't distinguish between maternal and paternal. When and how must be better organized.

"VAFs of truncal SNVs overlapping with various copy numbers inferred by DeepCopy on ovarian cancer patient OV2295 [20]. VAFs are shown for all the top-11 copy numbers with the most SNVs (or equivalently, all copy numbers with at least 140 S"
Fig S10: SO Chisel and Signals do really well earlier. It would be interesting to see how well they do wrt this test.

"and the color of the nodes matches the UMAP clusters"
Fig S20: This means that those clusters are far from monophyletic; that is, either the trees or the clusters are far from correct.

**Reviewer 3**

**Were you able to assess all statistics in the manuscript, including the appropriateness of statistical tests used?** Yes I am able to assess all statistics in the manuscript

**Were you able to directly test the methods?** Yes

**Comments to author:**

The authors proposed DeepCopy for haplotype-specific copy number calling on scDNA-seq data. DeepCopy applies a deep reinforcement learning system to correct and estimate the final copy number profile from 'raw' copy number profiles initially estimated from low-noise regions by NaiveCopy. By comparing DeepCopy with CHISEL, Alleloscope and SIGNALS on simulated and real data, they claim that their evolution-aware model produces more realistic clones with identical CN profiles, leading to more parsimonious CNA phylogenies. I think the use of reinforcement learning for CNA calling is an excellent idea. However, there are still some issues that need to be addressed before it can be accepted for publication.
Major:
1. The method descriptions are too technical in Methods and Supplementary sections, while too simple in the Results section. It would benefit a broader audience if a more intuitive introduction to the DeepCopy model could be provided in the main text and Method sections. In particular, a brief description of the intuition behind the reinforcement learning framework and a more detailed explanation of how it is adapted to the CNA calling task would benefit readers unfamiliar with reinforcement learning.
2. Can you clarify the generating process. If I understand correctly, the process generates a new CNA tuple given the current state which is represented by CNA profiles Ρ. The process stops at a generated STOP state. However, it is not clear to me how the elements in CNA tuple are generated. In Line. 390-393 and figure 6d, they assign a probability to each element, how these element wise probabilities were joined to represent the probability of the new tuple? In addition, the probability for each element seems to be generated by a softmax function which gives a distribution of all possible output state, do you choose the best state for each element and combine them to form the final output tuple to be generated?
3. Does the above generating model allow CNAs to be generated on overlapping regions on the same haplotype? Do you constrain the number of events to be generated?
4. How is the CNN structure related to the generating model? A graphical representation of the structure may be beneficial.
5. In the simulation set-up how do you assign fitness values?
6. Is DeepCopy sensitive to the estimates of R, B and \Sigmas? Will unreliable estimates of R, B and \Sigmas, distort the results of DeepCopy?
7. DeepCopy samples from the initial estimates from NaiveCopy. How different is the final profiles P compared to P-tilde?
Minor:
8. The prior based on the generating process in DeepCopy seems to improve the estimation of CNAs at the trunk or in large subclones. Would it miss or be less sensitive to low frequency CNA?
9. Does other methods also make consistent estimates across sequencing technologies in section 2.5?
10. Fig 3b and 4b, the region with small CNAs can be circled or highlighted in the figure for better understanding.
11. In the method description DeepCopy and NaiveCopy apply an initial bin size of 100kb. Is it necessary to adjust this bin size when applied to ultra shallow technologies? Say ACT.
12. The author used many mathematical notations and symbols in Methods and Supplementary sections. It is recommended to include a glossary table with the list of notations.

**Authors Response**

**Point-by-point responses to the reviewers’ comments:**

We would like to thank the editor for handling our manuscript and the reviewers for their helpful feedback and analysis suggestions. We have performed a variety of additional analyses, including several analyses justifying our cell-specific copy number profiles and several analyses comparing predicted copy numbers with read depth. Additionally, we have added benchmarking on CNAsim simulations, comparisons with the total copy number calling algorithm VICTree, and updated our parsimony estimates to use Lazac. We have also updated the writing to improve readability by adding intuitive explanations and examples, as well as moving more content to the methods section to avoid an overly layered presentation. Please find below our point-by-point response (indicated in blue).

**Reviewer 1:**

Reviewer #1: The authors describe DeepCopy, a method for inferring copy number states from single cell whole genome sequence data. The method uses an evolutionary model and a reinforcement learning algorithm to infer copy number states jointly across a population of related tumor cells. The method is interesting and will almost certainly be useful in single cell cancer genomics projects. The benchmarking experiments are well constructed. My main concern is that DeepCopy may be insensitive to cell specific CNA. The profiles produced by DeepCopy look cleaner than the other methods, but is that at the expense of true cell specific events? Unfortunately I dont believe the current benchmarking results clearly evaluate DeepCopy's sensitivity to such events.

We agree that producing cleaner appearing profiles does not imply a more accurate method, since a method could indeed be missing legitimate events. CNRein (formerly DeepCopy) attempts to balance evolutionary constraints with observing true rare events. We thank the reviewer for the suggestion to assess sensitivity to cell-specific CNAs. We have added substantial additional analyses (together with clarification of existing results) to fully demonstrate our accuracy on cell-specific CNAs on both simulated and real data. Briefly, for simulated data with varying level of intra-tumor heterogeneity (ranging from very few cell-specific profiles to most cells having cell-specific profiles), we find that our method more accurately reconstructs cell-specific profiles than existing methods. On ovarian cancer patient OV2295, CNRein identified 408 out of 617 cells to have a cell specific copy number profile not observed in any other cell. Meanwhile, in breast cancer patient S0 there is much less heterogeneity, with only 113 out of 783 cells having cell-specific copy number profiles.

Major:

1. Why are larger clones more desirable in output on real data? Do you believe that many of the cells sequenced have truly identical copy-number profiles, or is it merely the case that the per-cell coverage and data quality limit recovery of cell-specific events? I think the authors should comment on this in the introduction and/or discussion, because while indeed inferring fewer larger clones is more parsimonious, it is not obvious that it is a desirable goal or ultimately a more accurate description of the underlying biology.

Yes, we believe that per-cell coverage and data quality limit the recovery of cell-specific events. We have included the following sentences in the Background (formerly Introduction):

*“Our key premise is that one should not infer CNAs in the absence of clear evidence from data and rather opt for shared clonal structure. In particular, one should only infer cell-specific CNAs when there is strong evidence in the read depth or BAF data.”*

With this being said, our method does detect cell-specific events when there is sufficient evidence. On simulated data, we accurately reconstruct the number of cell-specific copy number profiles both in cases where cell-specific copy number profiles are rare and in cases where nearly all copy number profiles are cell-specific (Fig. 2d and S5). On ovarian cancer patient OV2295, 408 out of 617 cells have cell-specific copy number profiles. By contrast, in breast cancer patient S0 CNRein identifies less heterogeneity, with only 113 out of 783 cells having cell-specific copy number profiles. Breast cancer patient TN3 has an even lower level of intra-tumor which is consistently seen with both sequencing technologies. Specifically, only 54 out of 1101 cells sequenced with ACT technology are predicted to have a cell-specific copy number profile and only 36 out of 1070 cells sequenced with 10x sequencing technology are predicted to have a cell-specific copy number profile.

2. Does DeepCopy perform any correction for errors in the reference-based haplotype phasing within bins? If not, what enables the subsequent steps to be robust to switch errors? Using SHAPEIT with the 1000 Genomes reference panel, SNPs within 100kb of each other can be phased incorrectly up to ~10% of the time (Fig. 3A from Choi et al., PLoS Genetics 2018).

Yes, CNRein performs corrections for noisy input signals, including BAFs obtained from reference-based haplotype phasing. However, we do not explicitly have a step in the algorithm for targeting SHAPEIT within-bin phasing errors. What may aid in robustness is that, unlike some existing methods, we did not fix the size of haplotype blocks. Instead SHAPEIT automatically phases into variable sized haplotype blocks typically smaller than 100kb (average length 25.91kb on ovarian cancer patient OV2295). Thus, for OV2295 our 100kb bins typically contain 4 haplotype blocks. We phase these haplotype blocks into 100kb bins using our SVD-based haplotype phasing algorithms. The benefit of phasing small haplotype blocks with SHAPEIT is a low rate of incorrect phasing within haplotype blocks (as shown by Fig. 3A from Choi et al.). However, this does place substantial importance on correctly phasing haplotype blocks together to form allele specific counts in bins. Despite this, there are likely some cases of incorrect phasing either due to SHAPEIT or due to our phasing algorithm. Incorrectly phasing some haplotype block within a bin would result in allele-specific counts that are distorted from their true values. This happening in an individual bin is unlikely to shift the overall BAF of a segment substantially enough to influence copy numbers. This is especially the case since changes of BAF in individual bins of a segment increase our noise estimate of the segment, and thus decrease our sensitivity to the BAF measurement. If incorrect phasing occurs systematically (rather than in a few specific bins), this would slightly shift BAF values towards 1/2 (and away from either 0 or 1), since purely random phasing would result in a BAF of 1/2 everywhere. This effect could become problematic and result in missed LOH if one used a binomial model in which LOH is assumed to never generate reads on the minor allele (or at least very rarely generate reads on the minor allele if some correction is added). Instead, we use a Gaussian model of BAF so that slight distortions towards a BAF of 1/2 have very little effect on our copy number estimates.

3. The SVD-based haplotype phasing algorithm is very interesting but it is not clear to me why a rank-10 matrix approximation is necessary, what information it encodes, and overall how the algorithm accomplishes the goal of aligning phasing blocks across the chromosome. Are the "groups" of 100 haplotype blocks overlapping? Why an exponential moving average?

A rank-1 matrix assumes that a single linear combination of cell allelic imbalances allows phasing across the chromosome, which is sufficient in chromosomes with simpler variation. On the other hand, a rank-*k* matrix contains *k* > 1 different linear combinations of cells’ allelic imbalances (or more precisely z-values of allelic imbalances), allowing for different signals to be used for phasing different parts of the chromosome. This is necessary to accommodate large variations across the chromosome that result in different subsets of cells (or different linear combinations) being useful for phasing. To phase blocks across a chromosome, we first phase groups of 100 (non-overlapping) blocks using the 10 singular values (by reducing them to a single singular value within that subset of blocks), and then phase consecutive groups using an exponential moving average. We use an exponential moving average because it phases each group not only considering its previous neighboring group but also boosts the signal using earlier groups (in case the previous group is highly noisy with low read counts). The exponential moving average specifically balances signal from the closest previous groups with signal from groups with higher read counts by assigning exponentially lower weight to groups further back in the chromosome.

While in most cases, phasing with a rank 1 matrix and avoiding all other complexities (rank 10 matrices, groups of haplotype blocks, and exponential moving averages) would be fine, in certain scenarios with large changes in the copy number signal used for phasing across the chromosome, these additional complexities may be useful. Here is a convenient hypothetical for demonstrating the point. Imagine that the first 50 cells have the A allele duplicated across all of chromosome 1. Imagine all 2000 remaining cells have a duplication of the A allele on the first fourth of chromosome 1, and a duplication of the B allele on the last fourth of chromosome 1. A rank-1 based analysis would primarily use the imbalance on all 2000 cells for phasing, and would incorrectly phase the 2000 cells to have a duplication of the A allele on both the first fourth and last fourth of the chromosome. In fact, any reasonable procedure that attempts to phase all the haplotype blocks together at once without considering the relative location of different haplotype blocks would make this mistake. Algorithms like SIGNALS use HMMs to calculate phasing while taking into account the position of bins within the chromosome, and thus could also avoid this issue.

Instead, our procedure would work as follows. First, the rank-10 SVD would determine two primary components for phasing. (1) The imbalance of the second 2000 cells. (2) The imbalance of the first group of 50 cells. The additional 8 components would likely only capture noise and have small variations. Phasing individual haplotype blocks within groups of 100 becomes much easier, since the phasing only needs to consider 10 singular value components, rather than the individual values of all 2050 cells. Specifically, it considers the sum of imbalance of the first 50 cells and the sum of imbalance of the next 2000 cells. Phasing groups of 100 haplotype blocks using these numbers should provide the correct phasing in each group of 100. These groups of 100 haplotype blocks are not overlapping. We assume major changes in copy number that dramatically affect phasing do not occur within individual groups of 100 haplotype blocks. Finally, each group of 100 has a much stronger signal than individual haplotype blocks, providing a strong enough signal that phasing these groups is relatively easy as long as one uses some reasonable algorithm that considers the relative position of these groups. We use an exponential rolling average for simplicity (given the complexities of the rest of our phasing algorithm), however, we believe an HMM would also work well.

In our example, the first fourth of the chromosome is easily phased using the strong signal of 2000 cells. Then, the first 50 cells provide a signal to correctly phase the middle half of the chromosome with the beginning of the chromosome. Finally, the first 50 cells allow for correct phasing of the middle half with the final forth of the chromosome. Then, the strong signal of the 2000 cells provides a strong signal for easily phasing of the final fourth of the chromosome.

Although this hypothetical is artificial, the general point is that using a higher rank matrix (rather than rank 1) allows for better phrasing when there is substantial variation across the chromosomes in which cells have allelic imbalance and in which direction. This is the case while maintaining the advantage of the SVD-based phasing, namely, reducing noise on cells by simplifying to a few singular components that meaningfully indicate phasing across the chromosome.

4. What evidence is there that the fewer copy-number profiles inferred by DeepCopy represent a better fit to the data? Can you contrast the cell-specific events inferred by DeepCopy (i.e., in clones of size 1) with those inferred by other methods to show that DeepCopy only combines cells into clones where there truly is insufficient evidence for cell-specific events?

On simulated data, we have added an additional analysis to demonstrate that the cell-specific copy number profiles produced by our method are legitimate, whereas other methods produce illegitimate cell-specific copy number profiles. This is done in the Supplementary Section “Accuracy in detecting small clones”. Specifically, we now analyze the predicted clone size according to each method for cells with a ground truth clone size ranging from 1 to 10, where clone size 1 indicates a cell-specific copy number profile. For each ground truth clone size N, we calculate the set S_N of cells that belong to clones of size N in the ground truth. We then observe the predicted clones according to our method, SIGNALS, and CHISEL. The predicted clone size for a cell s is equal to the size of the clone that cell s belongs to on the predicted copy number profiles. Using this, we calculate the median predicted clone size for each set S_N. If a method perfectly reconstructs the clonal structure then S_N would be equal to N for all N. If a method randomly reconstructs clones unrelated to the ground truth clones, then S_N would be uncorrelated with N. The below plot this median value for N ranging from 1 to 10 for each method (pooled across all 20 simulation instances).

As shown in the plot, our method very accurately reconstructs these small clone sizes, including cell-specific events (N=1). In contrast, for SIGNALS and CHISEL the median predicted clone size is 1 (indicating a cell-specific event) independent of the ground truth clone size. This very strongly demonstrates the accuracy of our method at detecting small clone sizes including cell-specific events on simulated data. To show more information beyond the median, we also generated the below boxplots of predicted clone sizes for our method, SIGNALS, and CHISEL.

On real data, this is much trickier to demonstrate since we do not know the ground truth cell-specific events. It is a complex problem to determine if two cells truly have sufficient differences in read counts to justify inferring distinct copy number profiles. As a proxy, we compute for each cell the L1 distance to its closest cell in terms of normalized read depth. Then, we compare the distributions of these distances for cells that are designated as having cell-specific copy number profiles vs. those that have shared copy number profiles. We applied this analysis to the ovarian cancer patient OV2295, as it had by far the largest proportion of cell-specific copy number profiles according to CNRein’s predictions (408 out of 617 cells [66.1 %]). We find statistically significant larger L1 distances for cells with cell-specific copy number profiles in OV2295, indicating distinct patterns of read depth justifying their cell-specific profile. This analysis is included in supplementary section “Comparing copy numbers with read depth”.

5. Previous analysis of OV2295 (see Funnell 2022, Extended Data Fig. 3), showed scattered WGD in the non-WGD sample. Does DeepCopy predict the same? If not, why not?

Yes, our predictions also show this. The non-WGD sample is SA1090 and of 220 cells, 10 cells are predicted to have WGD (as indicated by a ploidy above 3). Below is a heatmap of copy number profiles for cells in SA1090 (the non-WGD sample). At the top one can see a distinct subset of WGD cells.

Zooming into the WGD cells gives the below heatmap.

6. How does the use of ZCNT interact with clonal or subclonal WGD in simulations and real data?

In our original analysis, we specifically modified the ZCNT distance to properly consider WGD. In terms of how our parsimony approach interacts with WGD we previously used a modified ZCNT* distance between methods. Specifically, this modified distance between two profiles P and Q takes the minimum of ZCNT(P, Q), ZCNT(P, 2Q) + 1 and ZCNT(2P, Q) + 1. Thus, a WGD is considered to be a single event, giving a more evolutionarily realistic distance metric.

However, Reviewer 2 states a strong preference for simplifying our paper by using the existing method Lazac from the ZCNT paper. Lazac does not specifically consider WGD and therefore treats WGD as separate CNA events on each chromosome. However, due to the large scale of parsimony values in our analysis in comparison to the number of CNAs required to replicate a few WGD events, we empirically found very little difference between our parsimony scores and Lazac parsimony scores as shown in the below plot of parsimony values on our simulations profiles (including the ground truth profiles and predicted profiles).

Similar agreement also occurs on real data as shown below.

7. The DeepCopy profiles for OV2295 look more smoothed over than those for chisel or especially signals (ignoring allelescope which seems to be having trouble). Can the authors be sure those cell specific CNVs are not real? Showing the raw read depth alongside the inferred copy number state will help show whether the cell specific events are spurious.

We have now performed a general analysis of fit to raw read depth with details given in response to comment 8, providing an overall metric demonstrating that our predictions do not have an overall deficit in terms of fitting the read depth data. In addition to this evaluation, we now specifically consider fit to read depth in the case of cell-specific CNAs on ovarian cancer patient OV2295.

A CNA is defined by its start position, end position and allele specific copy number. Two cells are said to have the same CNA if they share the same region of constant copy number with both the same copy number and the exact same start and end position. Using this definition, we find all CNAs that only occur in one cell and are thus cell-specific CNAs. To compare with read depth, we scale the read depth for each cell to have the same average value as the average total copy number of that cell (according to the prediction of whichever method we are analyzing). Before statistically analyzing whether inferred cell-specific CNAs are spurious or valid, we first provide examples. Below are some arbitrarily chosen examples of cell-specific CNAs for SIGNALS, where the read depth is scaled to have the same average value as SIGNALS average total copy number. Predictions according to CNRein are also included.

Visually, the top left plot and top right plot appear to be spurious deletions and amplifications respectively. The bottom left plot could be either spurious or legitimate. If there was a consistent increase in read depth across many cells, it would seem legitimate. However, since this CNA event is cell specific, it is very unclear whether it is spurious or legitimate. For the bottom right plot, SIGNALS infers a small total copy number 3 region between the larger total copy number 4 and total copy number 2 regions, which seems to be spurious.

Visually analyzing these plots is helpful for building intuition, but it is also entirely subjective. To objectively quantify spurious cell-specific CNAs one must consider signals in the read depth that would be likely to occur for spurious cell-specific CNAs but not for legitimate cell-specific CNAs. Applying L1 distance with read depth is not helpful since calling more cell-specific CNAs in higher noise cells does not necessarily imply spurious CNAs. Instead, we note that legitimate cell-specific CNAs with some copy number *C* will on average have read depth signals centered around *C*. In contrast, if the true total copy number is *C* but the read depth is closer to *C’* due to noise, the read depth is more likely to be biased towards being between *C* and *C’* rather than being centered exactly at *C’* on expectation. This type of effect may be difficult to detect for individual cell-specific CNAs. However, if spurious cell-specific CNAs appearing to have total copy number *C’* are systematically caused by noise in the read depth in some region with legitimate copy number *C* one generally expects to find a lopsided distribution in the read depth. More generally, if cell specific CNAs with copy number *C’* are frequently spurious and tend to have some other true copy numbers, this may be reflected in lopsided scaled read depths. In contrast, if cell-specific CNA events for copy number *C’* are generally legitimate, one does not expect a lopsided distribution.

We first calculate all cell-specific CNA events that result in a given total copy number *C*. For each such CNA, we identify all 100kb bins that intersect with the CNA and record the scaled read depth for each cell of those bins. Performing this for copy number *C* = 3 gives the below distributions for our method, CHISEL, and SIGNALS.

One can immediately see that SIGNALS has far more bins affected by cell-specific CNAs (216,510) than our method (26,362) or CHISEL (32,794) with total copy number *C* = 3. Additionally, one can see that the values for SIGNALS are lopsided towards values below 3, potentially indicating spurious cell-specific CNAs. For CHISEL, the distribution visually looks somewhat odd and lopsided, but is not too clearly biased. One potential metric is skewness, defined as the mean minus the median divided by the standard deviation. Skewness generally measures how far the mean is shifted towards one side or the other relative to the median. However, rather than measuring relative to the median we can measure relative to the true value the distribution should theoretically be centered on, namely the copy number. Doing so gives the modified skewness metric of mean minus copy number divided by standard deviation. For copy number 3 our method, SIGNALS and CHISEL have modified skewness values of 0.11, 0.19, and 0.12, respectively.

We analyzed all copy numbers from 1 to 10, and provide histograms for some common copy numbers (for which there are at least 10,000 modified bins in cell-specific CNAs for all methods), as well as a table of all skewness values. Below are the histograms.

Below is a table of modified skewness values together with total bin counts for copy number for our method, CHISEL, and SIGNALS for copy numbers 1 through 10.

|  | Modified skewness | | | Total number bins | | |
| --- | --- | --- | --- | --- | --- | --- |
| Copy number | CNRein | SIGNALS | CHISEL | CNRein | SIGNALS | CHISEL |
| 1 | **0.71** | 0.84 | 0.73 | 23,448 | 120,245 | 6,649 |
| 2 | 0.27 | **0.09** | 0.25 | 23,983 | 227,944 | 17,373 |
| 3 | **-0.08** | -0.29 | 0.09 | 26,362 | 216,510 | 32,794 |
| 4 | **0.03** | -0.09 | 0.06 | 30,668 | 146,715 | 27,142 |
| 5 | **-0.18** | -0.26 | -0.27 | 35,441 | 90,178 | 29,958 |
| 6 | **-0.24** | -0.53 | -0.44 | 22,723 | 58,172 | 18,751 |
| 7 | **-0.42** | -0.58 | -0.58 | 15,821 | 23,520 | 7,573 |
| 8 | **-0.36** | -0.63 | -0.82 | 8,010 | 10,859 | 5,599 |
| 9 | **-0.44** | -0.64 | -0.61 | 5,542 | 5,395 | 465 |
| 10 | **-0.28** | -0.61 | -0.34 | 3,563 | 3,289 | 1,450 |

One can see that SIGNALS has the largest modified skew for most copy numbers (1, 3, 4, 6, 7, 9, and 10) and our method has the smallest modified skew for all copy numbers other than 2. It is also apparent that SIGNALS has by far the most bins affected by cell-specific CNAs. While our method and CHISEL have similar numbers of bins affected by cell-specific CNAs, it is worth noting that cell-specific profiles can also be caused by unique combinations of CNAs even if each individual CNA is not cell specific. This analysis is included in Supplementary Section “Comparing copy numbers with read depth”.

8. The claim that "DeepCopy avoids spurious CNAs producing a more parsimonious solution than SIGNALS or CHISEL" is not supported by the evidence. Using parsimony cannot assess whether DeepCopy smooths over cell specific events, or whether signals or chisel predicts spurious CNA". Instead, an appeal to each method's fit to raw read depth signal may help here.

Yes, using parsimony cannot assess whether CNRein smooths over cell-specific events or SIGNALS/CHISEL predicts spurious CNAs. To account for this, we included an SNV-based orthogonal validation. However, we agree with your suggestion and now also measure how well each method fits read-depth data. Specifically, as described in the Supplementary Section “Comparing copy numbers with read depth”, we scale both the total copy number and the read depths to have an average value of 1 across the genome to make them comparable. Then, we compute the average L1 distance between the scaled predicted total copy number and the scaled read depth (across all bins) for each cell and for each method. Below is a boxplot showing these results for all four methods on ovarian cancer patient OV2295.

Applying the t-test, we observe that our method matches the read depth data better than CHISEL (p = 7 * 10^-30), and Alleloscope (p < 10^-277). Our method and SIGNALS match the data nearly identically well with no statistically significant difference (p value 0.314). Additionally, below is a box plot showing this analysis for breast cancer patient S0.

Our method matches the read depths better than CHISEL (p=2.5 * 10^-6), and slightly better than Alleloscope (p=0.0043). There is no statistically significant difference in matching the read depth between our method and SIGNALS (p value 0.093).

This read depth analysis is a helpful addition to our paper working with evaluations. However, one should note that simply rounding read depths to the nearest integer would give the best copy number caller by this metric. Thus, it’s important to keep in mind the context of other evaluations when observing these values.

9. One of the main methods by which the authors evaluate copy number, and indeed a major contribution of the paper, is to construct a tree and then calculate a parsimony score. If the copy number is overly smoothed, removing cell specific CNAs, building a phylogeny may be more difficult. A worse parsimony score may thus be due to the increased difficulty in tree building rather than inaccuracies in copy number estimation. In other words, the parsimony evaluation method may bias against calling a higher amount of true cell specific CNA. Can the authors provide evidence this is not the case? Applying tree building plus parsimony scoring to true copy number from simulations with varying amount of cell specific CNAs would help rule out this phenomenon or at least identify the degree to which it is a problem.

Yes, we completely agree that parsimony is only part of the picture and that a method could achieve a good parsimony score by oversimplifying and smoothing over cell-specific CNAs. On real data, we account for this via SNV-based orthogonal validation and now additionally evaluate fitting read depth following your suggestion.

We strongly agree that simulations with varying amounts of cell-specific CNAs are essential evidence for ruling out this phenomenon. Consequently, we have now further clarified in the text that this is the precise motivation for the construction of our existing simulation instances. Our simulations are constructed with varying levels of cell-specific CNAs (as a consequence of varying levels of clonal fitness) in order to show how well methods fits varying levels of intra-tumor heterogeneity and to ensure our method neither smooths over legitimate CNAs nor fabricates spurious CNAs. Figure 2d shows the true number of unique copy number profiles as well as the predicted number of unique copy number profiles. Note that few unique copy number profiles imply few cell-specific profiles and many unique copy number profiles imply many cell-specific profiles. For instance, one unique copy number profile would imply zero cell-specific profiles and 1000 unique copy number profiles (on 1000 cells) would imply all cells have cell-specific profiles. In Figure 2d, one can observe that SIGNALS and CHISEL predict unique profiles for nearly all cells. In contrast, our method accurately reconstructs the ground truth number of unique copy number profiles (as indicated by the bold red line). Our method does not smooth over legitimate cell-specific CNAs, as that would result in systematically fewer unique copy number profiles in our predictions than the ground truth. Additionally, in the below plot (now included in the supplementary materials) we have directly measured the number of cell-specific copy number profiles (not the total number of clones indicated by the total number of unique profiles).

Additionally, isolating to only CNRein, SIGNALs and CHISEL for clarity gives the below

This demonstrates the accuracy of our method in reconstructing the ground truth number of cell-specific copy number profiles.

In terms of parsimony, Figure 2e shows that our method matches the simulation ground truth parsimony much more accurately than SIGNALS or CHISEL. However, we still tend to produce slightly less parsimonious solutions than the ground truth, indicating there still exists some room for improvement. In summary, our simulation results indicate that we do not smooth over legitimate CNAs in order to produce overly parsimonious solutions.

The below text has been added to clarify this purpose of our simulations.

*“On simulated data, we directly evaluate the accuracy of our method as well as its effective reconstruction of the ground-truth level of intra-tumor heterogeneity.“* (added to the beginning of the Results section).

*“The probability of fitness increases controls the relative growth rates of clones and thus the overall level of intra-tumor heterogeneity including the number of cell-specific CNAs.*

*This varying heterogeneity helps us evaluate if methods underestimate heterogeneity by missing legitimate CNAs or overestimate heterogeneity by inferring spurious CNAs.”* (added to the simulation results section).

10. The authors claim that "DeepCopy avoids spurious CNAs by producing a far more parsimonious solution" (line 240). This does not seem to be well supported by their current benchmarking. Specifically, it is not clear that using SNV likelihood based evaluation, a great idea in general, will be sufficiently sensitive to discern between methods that predict spurious cell specific CNAs, identify true cell specific CNAs, or smooth over true cell specific CNAs.

Thank you for your appreciation of our SNV likelihood based evaluation. We agree that it is not sufficiently sensitive to determine very minor differences in accuracy, such as those between SIGNALS, Alleloscope and our method on breast cancer patient S0 or between SIGNALS and our method on ovarian cancer patient OV2295. To evaluate the sensitivity of our SNV likelihood based evaluation to cell specific CNAs, we created a very crude smoothed-over post processing of our methods predictions, removing all cell specific CNAs on ovarian cancer patient OV2295. We then determined the log likelihood score between these overly smoothed post-processed predictions and our original predictions. Specifically, the smoothed-over post processing was done by taking all cells with cell-specific copy number profiles and assigning them to the closest (L1 distance) copy number profile that exists in multiple cells. This resulted in 58 unique copy number profiles in total. We found a log likelihood ratio of 3,239 between this modification and our original prediction (p-value < 10^-5). This is much larger in scale than the difference between our method and SIGNALS (-1,033), but similar to the log likelihood ratio between our method and CHISEL (3,418). This provides some evidence that SNV likelihood based evaluation can detect if a method consistently smooths over legitimate cell-specific CNAs. However, since this meaningful difference in the log likelihood required crudely removing all cell-specific profiles, we agree that our SNV likelihood based evaluation is not sensitive to missing a few true cell specific CNAs. To supplement this, we also implemented the suggested read depth based evaluation described in our previous comment, as well as adding text clarifying how our simulations provide additional evidence for avoiding spurious CNAs.

Minor:

1. The input format requires cells in the BAM to be labeled by read group instead of CB tag. Labeling cells by CB tag is preferable. Droplet based methods produce too many barcodes to be stored as one barcode per read group. Also a merged bam with many read groups is incompatible with pseudobulk SNV and breakpoint calling. Please add support for CB tag based BAM input.

We have now added this feature.

2. In the segmentation algorithm, did you experiment with setting Gamma smaller than 10 to recover smaller copy-number segments? How are the 6 evidence values combined for each candidate breakpoint?

Setting Gamma smaller than 10 tends to not determine statistically meaningful segments. For such small segments, it is difficult to determine meaningful CNAs. Additionally, increasing the number of segments increases the computational cost of our method. To check that this is the case, we have now additionally run our method with Gamma = 5 included on ovarian cancer patient OV2295 and breast cancer patient S0. Overall we find the predictions on the ovarian cancer patient are slightly (but not substantially) worsened and the predictions on the breast cancer patient are not meaningfully affected. Below are heatmaps of the original predictions together with the predictions with Gamma = 5 included for both patients.

We also performed the SNV-based analysis on these predictions with Gamma = 5 included. Below are the results on ovarian cancer patient 2295.

Although the relative ordering of methods stays the same, the accuracy of our method was somewhat diminished. The original log likelihood with SIGNALS, CHISEL and Alleloscope were -1,033, 3,418 and 12,635. However, with Gamma = 5 included, the log likelihood ratios were -2418, 2033, and 11,582 respectively. On breast cancer patient S0 we performed the SNV based orthogonal validation and found very little change from our original results. Below is the histogram.

Specifically, the log likelihood ratio with SIGNALS, CHISEL and Allleloscope in the original results were 145, 1,093 and -35. However, with Gamma = 5 included, the log likelihood ratios became 169, 1,175 and -35 respectively. In addition to performing the SNV-based orthogonal validation, we calculated parsimony scores. On the ovarian cancer dataset, the parsimony values increased to a slightly higher level than CHISEL as shown below.

In contrast, on the breast cancer dataset there was no substantial increase in the parsimony score as shown below.

In terms of combining evidence across the 6 values, we determine a breakpoint to exist if the evidence value exceeds the threshold for any of the 6 evidence values. The evidence is not summed across the values. The reason for this is that some of the same information is being used to calculate the evidence for different values of Gamma and thus it is statistically dubious to add up the evidence. For instance, half of the window for Gamma = 20 is the window of Gamma = 10. With this technical justification being said, we believe that other methods such as summing the evidence would likely not give any practical issues. We have added the following sentence to the text to clarify this (in Supplementary Section “Estimating segments and noise levels”).

“*We determine a breakpoint to exist if there is sufficient evidence for any of these Gamma values individually and do not sum evidence across multiple gamma values.*”

3. In what sense is the relaxation to a Gaussian distribution using the central limit theorem valid in single-cell sequencing data when each bin may have fewer than 50 reads, with much fewer reads covering SNPs within the bin? Does this refer to a relaxation at the segment level?

Yes, this refers to a relaxation at the segment level for which read counts are higher. Specifically, we have the below histogram of the average number of reads per segment for all cells in the DLP+ ovarian cancer dataset.

Isolating to only allele-specific reads which cover SNPs, we have the below histogram.

The read counts are much lower, but still sufficiently high for the central limit theorem to reasonably apply. Moving on to breast cancer patient S0 (10X CNV) we have the following histogram of read counts (on the original set of cells including normal cells).

Then isolating to reads that cover SNPs we have the below histogram.

This again shows reasonable read counts for applying the central limit theorem at the segment level, as pointed out by the reviewer.

4. The choice of name DeepCopy may not be ideal, as it is also a commonly used computer science term which could make the method much harder to find. Also, there is already a method DeepCNA (Liu et al., BMC Genomics 2024) for inferring CNAs from scDNA-seq, which could be confusing.

We agree. In fact, we were uncertain of the ideal name to use for these exact reasons. We have now changed the name to CNRein, which stands for copy number reinforcement learning.

5. Some sections, especially methods, are complex without adequate explanation. For instance "B.2 Detection of small CNAs" relies on formulas almost exclusively and a few sentences would help the reader understand what the formulas mean.

We have added more explanatory and intuitive sentences throughout our methods content. In the section “B.2 Detection of small CNAs”, we have added easily understandable intuitive sentences before each of our mathematical definitions.

6. "vast majority of truncal SNVs seem to precede their coinciding CNAs" -> I would add CNAs and WGD here if that in fact is true.

We have now modified the text to include WGDs.

7. Line 213 " small CNAs in SIGNALS predictions likely due to noise", be specific about these CNAs. what are their sizes and cell specificity?

We agree that “small CNAs” is too ambiguous and have added “(around 1Mb or smaller)” to the text.

For cell-specificity, things are a bit more complicated because tiny single-bin CNAs are likely to line up in multiple cells even if they occur purely due to noise. For instance, if 1% of cells randomly have a CNA in a single bin due to noise, this would still result in multiple cells having an identical CNA (and thus not cell-specific). Additionally, some bins have a higher level of noise than other bins which may exacerbate this problem, causing spurious CNAs to not be cell-specific. However, this is easily counteracted by observing overall copy number profiles. Having multiple different random CNAs due to noise will nearly certainly generate a unique copy number profile even if some individual one-bin CNAs also exist in other cells by random chance. This is because there is no reason for multiple random CNAs to be shared on the same set of cells. Additionally, random CNAs will not nicely correspond to a true, resulting in high parsimony scores. Consequently, we believe it is cleaner to quantify noise using parsimony scores (and to a lesser extent the number of unique copy number profiles) and to avoid a more complex digression into quantifying cell-specific CNAs while accounting for small shared CNAs resulting from random chance.

Typos:

Supplement page 31 line 745 "moving exponential moving"

We have now corrected this.

Supplement page 36 line 33 "minimizing the blow"

We have now corrected this.

**Reviewer 2:**

Reviewer #2: General comments

This is a very interesting article describing a significant effort regarding allele-specific copy number calling for single cancer cells or subclones. Unfortunately, the article has several severe problems which are intertwined. The first two are concerned with the evaluation of the results and comparisons with other methods, which is related to the question about the purpose of the method: is the purpose copy number calling for individual cells or cancer subclones? After having read the submission, it is impossible to know if the proposed method is better than earlier methods. The third and fourth severe problems are that the authors have developed almost everything in the article independently. So, previously developed standard methods are not used, and many details are provided that make a better organization desirable.

CNRein (our method which was originally called DeepCopy but renamed in response to Reviewer 1) performs copy number calling for individual cells not cancer subclones. Depending on the data, the joint optimization procedure may result in subsets of cells having identical copy number profiles, or may result in assigning each cell a unique copy number profile. Importantly, our procedure does not utilize post-hoc clustering to generate subclones.

In response to the 2nd major comment about insufficient benchmarking, we have conducted new benchmarking analyses in addition to our existing comparison to ground-truth on realistic simulations and orthogonal validation on real data. In our simulations, we demonstrate that our method reconstructs ground-truth copy number profiles as well as levels of intra tumor heterogeneity better than existing methods. We have now also added CNAsim simulations (as suggested by the reviewer), which confirms the accuracy of our method over existing methods. On real data, we believe the combination of parsimony based analysis and orthogonal SNV-based analysis provide evidence that our method outperforms existing methods in terms of detecting legitimate CNAs and avoiding spurious CNAs. In response to the reviewer, we have also now clarified the difference between germline SNPs and SNV mutations in the tumor to help make clear the orthogonality of our SNV-based analysis (which are based on somatic point mutations that are not used during inference, not germline SNPs). In addition to this analysis, we have now additionally analyzed fit to read depth (as suggested by Reviewer 1), finding that our method continues to outperform existing methods.

In terms of using standard methods, we now compute parsimony using Lazac rather than our previously used novel approach, simplifying the writing and better standardizing our benchmarking. We also provide further explanation of our new techniques in comparison to existing methods. For instance, a new supplemental section explaining the similarities and differences between our reinforcement learning approach and GFlowNets (a relevant machine learning method mentioned by the reviewer).

Copy number calling for cancer clones regards clustering single cells into sub-clones and assigning all single cells to the same copy number profile in one sub-clone. When constructing a single tree from the copy number profiles, one can expect to get a lower parsimony score if each cell has been assigned a unique copy number profile. So the comparison between Deepcopy due to its regular ratio, induced by the RL framework, and copy number calls for individual cells made by the other method, for instance, Chisel, is unfair. Moreover, Chisel's original publication describes it as a method in which one of the final steps constitutes a clustering of the single cell copy number profiles and trees constructed in the publication or trees on sub-clones. In addition to these problems, there are other methods that I've been using over the last couple of years, such as Conet, Sitka, and VICTree. One course argued that they are not allele-specific, but they may still perform better than the allele-specific method presented here! So, a comparison is necessary.

As explained in response to Reviewer 1, our key premise is that one should infer cell-specific profiles only if sufficiently supported by the data. CNRein (just like VICTree and SCICoNE) uses an evolutionary model to balance fitting the data of each cell with forming a coherent evolutionary solution across cells. This is a fundamentally different approach than post-hoc clustering of copy-number profiles (as done in CHISEL). Regarding CHISEL, the default usage of CHISEL is determining copy number profiles for each cell and therefore these are the publicly available CHISEL predictions. These copy number profiles are generated by aggregating information **across cells** via “global clustering of genomic bins into copy number states”. However, we agree that it is worth comparing with CHISEL’s alternative clonal predictions for the sake of completeness. As shown in the below plot (reproduced from Fig. S7), clonal CHISEL performs similarly well as the original CHISEL on our simulations in terms of accuracy and L1 error (continuing to perform worse than CNRein).

Additionally, clonal CHISEL still does not accurately reflect intra-tumor heterogeneity. Specifically, as in the below plot, the number of predicted unique copy number profiles is independent of the ground truth number of unique copy number profiles.

In terms of parsimony, the parsimony values of clonal CHISEL’s copy number profiles are negatively correlated with the ground truth parsimony, in contrast the CNRein’s parsimony values which have a *r* = 0.95 Pearson correlation with ground truth parsimony.

Note, the best fit lines are curved due to the log axes of the plot. From these results we conclude that CHISEL’s clonal predictions are more parsimonious as a result of clustering cells but that these parsimonious solutions do not match the ground truth. On breast cancer patient S0, we found that clonal CHISEL produces a more parsimonious solution (parsimony value 2,187) than the original CHISEL prediction (parsimony value 9,877) but a less parsimonious solution than CNRein (parsimony value 630). Additionally, clonal CHISEL still scores much worse than CNRein on the orthogonal SNV-based analysis with a log likelihood ratio of 638.74 with a p value of 0.00241. The below plot compares clonal CHISEL’s results on this test with SIGNALS and Alleloscope.

Regarding methods for predicting total copy number, we did not originally include any such methods due to certain difficulties in comparing methods, however, we have now added such a comparison. On real data, our SNV based orthogonal validation is essential for determining if methods’ miss legitimate CNAs, since as the reviewer later points out, parsimony based evaluation is insufficient on its own to evaluate this. However, SNV based orthogonal evaluation cannot be used on methods that predict total copy number rather than allele-specific copy number. Due to this limitation and the fact that allele-specific copy number calling is a somewhat different task than total copy number calling, we originally did not include comparisons with such methods. With this being said, it is still worthwhile to compare a total copy number calling method to our method on simulated data for which evaluation is clearly possible.

The three total copy number methods the reviewer mentioned all require additional input information beyond raw read counts. Sitka appears to be a tree inference method requiring copy number inputs rather than a stand alone copy number calling method. As stated in Figure 1a of the Sitka paper, “(a) Sitka takes copy number calls data from a heterogeneous single-cell population.“ The method VICTree requires ploidy corrected read counts rather than raw read counts as stated in the paper “Bin counts are the only required input data and have to be corrected for GC content and ploidy (neutral CN equals 2).” However, this requires an estimation of ploidy which is very much non-trivial. Conet additionally requires a set of candidate breakpoint loci with the paper specifically stating the following. “Joint inference procedure of CONET and integer CNs. A CONET input consists of the corrected counts in bins (per-bin data) and set of candidate breakpoint loci; the data needs to be pre-processed with GC and mappability correction and normalization methods best suited for a given scDNA-seq technology.”

Taking all of this into consideration, we decided to run VICTree as a state of the art representative total copy number calling method, noting that the VICTree paper provides comparisons with CONET. Additionally, VICTrees ploidy correction on simulations is easy to provide by giving VICTree read counts correct with ground truth ploidy values. This gives VICTree a slight unfair advantage over other methods (since ploidy estimation is non-trivial), yielding an upper bound of VICTrees performance in comparison to other methods. We ran VICTree on CNAsim-based simulations as well as our own simulations. Details are provided in supplemental section “CNAsim simulations”, and we discuss these results in response to the reviewers comment suggesting CNAsim. Overall on CNAsim simulations, we find VICTree performs poorly in cases with whole genome duplications but performs only slightly worse than CNRein on simulations without whole genome duplication.

We also ran VICTree on our original simulations as shown in Supplementary Section “VICTree and CHISEL with clonal predictions”, and conclude that VICTree underestimates intra-tumor heterogeneity in contrast to SIGNALS and CHISEL which overestimate intra-tumor heterogeneity, and CNRein which accurately reconstructs intra-tumor heterogeneity. As a consequence, CNRein outperforms VICTree on 19 out of 20 of our simulation instances (in terms of both accuracy and L1 error). These results are shown in the below plots (which are included in the supplementary materials).

There are, in particular, three lines of research that the submission would have benefited from taking advantage of. The first is the so-called G flow nets (GFNs) described in several papers, mainly from Bengio's group. GFNs have been used to construct various types of bio sequences. The RL framework used in this article seems to be a less mature version of GFNs. One of the sampling steps used to find a normalizing constant could be better handled using GFNs.

Moreover, it would make the method presentation shorter and more standard.

GFlowNets (GFNs) have some conceptual similarities with our method and so we have now improved the text with several references to GFNs. However, GFNs do not directly apply to our problem. In terms of similarities, both our method and GFNs learn a distribution over trajectories to overall fit the data rather than simply trying to maximize the expected reward as is done most typically in reinforcement learning. In our setting, trajectories correspond to sequences G of copy number events starting from a normal cell. In contrast, GFNs do not have the same ultimate objective as our problem statement, namely maximizing the joint probability Pr(R, B | \theta) of all *N* cells’ read count data R and B. Given rewards R(P) for each terminal state, or copy number profile P, GFNs learn flows F such that the probability Pr(P | F) of reaching each terminal state is proportional to the reward R(P) for that state. Importantly, these rewards have to be specified ahead of time, which would not allow for solving our problem statement. (As a technical aside, rewards can also be given for non-terminal states, but that aspect of GFNs is not relevant for copy number calling.) To solve our problem statement, we evaluate the overall probability Pr(R_s, B_s | \theta) of a cell s given the probabilities Pr(P | \theta) assigned to copy number profiles P. We then optimize trajectory probabilities Pr(G | \theta) in order to maximize the product of cell probabilities Pr(R_s, B_s | \theta) across all cells s. Adaptive rewards are used together with policy learning to ensure gradients are in the direction of maximizing the joint probability Pr(R, B | \theta). While GFNs have the advantage of not requiring the estimation of a normalization factor Pr(R_s, B_s | \theta) as the review points out in other comments, calculating this normalization factor via sampling is not a major difficulty in our problem setting due to the limited number of cells and simple estimation procedure for each cell.

In addition to the specific similarities between GFlowNets and our method, there is also the general similarity that transition probabilities through a network of states are learned, forming probabilities for various trajectories. However, this also applies to standard policy learning. To further clarify, below is a table comparing and contrasting GFlowNets, our method, and the standard (fixed rewards) policy gradients approach.

| Method | GFlowNets | Our Method | Standard Policy Learning |
| --- | --- | --- | --- |
| Goal | Terminal state probabilities being proportional to rewards | Maximizing the product of cell probabilities, where each cell probability derives from a weighted sum of trajectory probability. | Maximize the expected reward. |
| Optimization method | Optimizing flows | Policy gradients with changing rewards | Policy gradients with fixed rewards |
| Learned structure | Flows proportional to transition probabilities between states | Transition probabilities between states learned via action probabilities given a state. | Transition probabilities between states learned via action probabilities given a state. |

It may be possible that some future work adaptation on top of standard GFNs would allow them to apply to our problem statement. However, for our current method we chose to instead adapt the very standard policy learning approach to apply to our problem definition. To highlight the possibility of alternatively trying to adapt GFNs to our problem statement, we added the following statement to our discussion section. “Another possible modification to our methods optimization procedure could be adapting GFNs towards our problem statement rather than our current procedure of adapting policy gradients.”

Additionally, we have added a supplementary section “Comparison of our reinforcement learning method to existing approaches” to address similarities and differences between our approach, GFlowNets, and standard (fixed reward) policy gradients.

Second, a technique called CNAsim was published by Weiner and Bansal, which can be used to simulate copy number evolution in cancer cells. Using this framework would shorten the presentation and facilitate a better comparison with other methods. For the site, the paper is. "A zero-agnostic model for copy number evolution in cancer." That paper describes a parsimony approach that could've been exploited in the submission rather than, as it's now done, using a few results from there to construct a new parsimony approach. Again, using the previously published method would have improved the submission's presentation and facilitated a better comparison with other methods.

We have extensively discussed the capabilities of CNAsim with the first author. Together with him, we have determined there are certain limitations that prevent it from fully replacing our simulations. One limitation is that they cannot effectively span the spectrum of intra-tumor heterogeneity which is an essential feature for our paper and essential to our responses to the other reviewers. Additionally, the allele-specific read counts do not take into account that only a small fraction of reads cover SNPs. Instead, all reads are assigned to one of the two alleles. Nevertheless, we have added analyses with the existing simulator CNAsim to the paper.

For these simulations, we utilized 5 different levels of coverage (0.2x, 0.1x, 0.05x, 0.02x and 0.01x) and the two placement types for simulating CNAs. Together with enabling or disabling whole genome duplications, this yields 20 parameter combinations which we have generated with 20 simulation instances. We measured the L1 accuracy for allele specific copy numbers for CNRein, SIGNALS and CHISEL. For VICTree we measured the L1 accuracy in terms of total copy numbers which is equivalent to assuming VICTree always optimally assigns the total copy number between the two alleles to minimize the allele specific L1 error. Additionally, as previously mentioned we provide VICTree with the ground truth ploidy by scaling read depths based on the ground truth ploidy (since this scaling is required by VICTree). Using these metrics, we have the below boxplot of average L1 error values on the 20 simulation instances for each method.

Our method has the lowest median L1 error of 0.049 somewhat outperforming CHISEL (median L1 error of 0.123) and greatly outperforming SIGNALS (median L1 error of 0.471) and VICTree (median L1 error of 0.990). We additionally have the below box plot of accuracy across the 10 simulation instances without whole genome duplication (noting that VICTree struggles with whole genome duplication but otherwise has a very nice performance).

CNRein continues to have the lowest median L1 error of 0.041, slightly outperforming VICTree (median L1 error of 0.056) and greatly outperforming SIGNALS (median L1 error of 0.183) and CHISEL (median L1 error of 0.213). It is also worth noting that the VICTree L1 error only includes total copy number errors while the CNRein L1 error also includes allele-specific errors. To investigate sensitivity to coverage, we generated the below boxplot of L1 error across simulation instances for different levels of coverage for each method.

Although our method has the lowest median error for all coverage values, one can see a rise in the error for very low coverage. We also analyzed these simulations using the median accuracy metric (in addition to L1 error), finding that CNRein has the highest median accuracy across all simulations, as well on subsets with and without whole genome duplication, and on subsets of each coverage level.

Regarding “A zero-agnostic model for copy number evolution in cancer”, we have now replaced our parsimony estimate with the Lazac algorithm from this paper. Our original algorithm was a slight modification of their Lazac-NJ algorithm by explicitly including whole genome duplication events in our modification of their ZCNT distance. Empirically, we find that using Lazac has very little impact on our results, while making our benchmarking more standardized (see our response to point 6 of Reviewer 1).

Finally, the submission requires a better presentation and organization. The multilayered organization, that is, with references to the method section, which contains references to supplements, etc., makes the paper very hard to read. Consequently, more material has to be presented in the same section. There's no reason to first introduce the measure probability and then later on actually define it., etc.

We have now modified the text to improve this. For instance, we have moved substantial amounts of information material from the supplementary materials sections to the methods. For instance, we moved the definition of the read count probability measure to the point in the methods text in which it is introduced. Additionally, we have moved the sections “Generating copy number profiles'' and “neural network architecture” from the supplement to the methods section. We have also removed the section “Data processing and NaiveCopy '' since this section mostly contained references to the supplement, making the text more multilayered. In addition to moving content, we have added a substantial amount of text with intuitive explanations of methods content to make the paper more readable without the reader having to move to check the supplement for clarifications. We have tried to make the text sufficiently clear that a reader does not need to look at the supplemental references in order to have an understanding of the results or methods content being discussed. Rather, the reader can use the supplement if they wish to have a highly detailed understanding of the technicalities of the content being discussed.

We also greatly appreciate all of your help with specific organization/writing points and suggestions given below. We have now improved the organization/writing following your below suggestions.

Specific comments

Evolution-Aware Deep Reinforcement Learning for Single-Cell DNA Copy Number Calling

Draft-- M

" evolution- 11 ary constraint"

10: You need a better intuitive description or what your evolutionary constraints are. It shouldn't go into the abstract but the introduction.

We added the below text in the Introduction to be more explicit about evolutionary constraints. Copy number profiles are constrained by evolutionary trajectories of CNA events, which are guided by a neural network.

*“CNRein constrains predicted copy number profiles to result from a sequence of amplifications and deletions of copies of specific alleles in contiguous regions within chromosomes. These sequences of CNA events form trajectories, starting from the normal cell, with a neural network constraining the likelihood of each CNA occurring. We use a deep reinforcement learning procedure to train the neural net to produce copy number profiles that maximize the probability of the observed read depth and BAF data. The predicted copy number profile for each cell balances fitting that cell’s read depth and BAF values with forming coherent evolutionary trajectories across all cells.”*

" A key limitation of these data is that 38 we do not measure read depth and BAF in individual clones but rather obtain composite measurements of 39 millions of cells in the bulk sampl"

37: This is basically saying that the limit of bulk data is that it's bulk data.

We agree and changed the sentence to:

*“Rather than providing measurement of individual cells, bulk DNA sequencing provides composite measurements of read depth and BAF of millions of cells in the bulk sample.”*

"verview of DeepCopy."

Fig 1: This has hardly any information.

We modified the figure caption to say “CNRein uses evolutionarily aware deep reinforcement learning to perform single cell copy number calling.”

" haplotype-specific copy number profiles P "

Fig 1d: For clones or cells? This should be made clear as early as possible.

The copy number profiles are for each cell. We have now clarified “haplotype-specific copy number profiles P for each cell” in Figure 1d. Additionally, to further clarify that these predictions are for each cell, we have added the following sentence to the Introduction:

“*The predicted copy number profile for each cell balances fitting that cell's read depth and BAF values with forming coherent evolutionary trajectories across all cells. Thus, the level of intra-tumor heterogeneity predicted by CNRein results from training on the data rather than the imposition of clonal structure via post-hoc clustering of inferred copy-number profiles.*”

" copy-neutral loss of heterozygosity and mirrored-subclonal 60 CNA"

59: I guess that this means that different alleles occur with number two in different cells. Please formulate this carefully.

Yes, it means that the copy number occurs on different alleles in two subsets of cells. We have clarified this as follows:

*“These algorithms have detected additional patterns on single-cell sequencing data not observable from total copy numbers such. Such patterns include copy-neutral loss of heterozygosity as well as mirrored-subclonal CNAs in which two subsets of cells contain the same number of gains or losses in the same genomic regions but on different alleles.”*

" have not "

60: Do not take

We have now corrected this.

" evolutionary process "

66: You need to supply more details about the model.

As previously mentioned, we have addressed this as follows:

“*We introduce CNRein, an evolution-aware deep reinforcement learning algorithm for haplotype-specific copy number calling. CNRein constrains predicted copy number profiles to result from a sequence of amplifications and deletions of copies of specific alleles in contiguous regions within chromosomes (Fig.1). These sequences of CNA events form trajectories, starting from the normal cell, with a neural network constraining the likelihood of each CNA occurring. We use a deep reinforcement learning procedure to train the neural net to produce copy number profiles that maximize the probability of the observed read depth and BAF data. The predicted copy number profile for each cell balances fitting that cell's read depth and BAF values with forming coherent evolutionary trajectories across all cells.*”

" on simulated data, "

71: What model is used for the simulation? Again I suggest CNAsim.

In addition to our own simulations (detailed in Supplementary Section “Simulation set-up”), we have now also added CNAsim simulations. Briefly, in our own simulations, a tree is formed by starting with a normal cell and sequentially adding CNA mutations to generate new nodes on the tree. Each new mutation has a probability of increasing the fitness of the clone (which is controlled by a parameter differing across simulations). Higher fitness clones have a higher prevalence and therefore have a higher likelihood of new clones forming from them. Additionally, once the tree has been generated, cells are sampled from clones proportional to their fitness. After the ground truth copy number profiles are generated, read counts are generated by mimicking the coverage and noise levels of breast cancer patient S0. Our simulations allow for a wide spectrum of intra-tumor heterogeneity by controlling the fitness increase probability parameter. However, we agree that it is also worth including independent simulations and thus we have added CNAsim simulations. For a discussion of differences/similarities between our own simulation procedure and CNAsim, please see our previous response on page 30.

" final copy number estimation via deep reinforcement learning. "

84: GFNs should be discussed in the intro.

We now state the following discussing GFlowNets in the Background section (previously Introduction).

*“Our deep reinforcement learning procedure has similarities with GFlowNets in that both methods learn a distribution over trajectories to overall fit the data rather than simply trying to maximize the expected reward as is done most typically in reinforcement learning.*

*However, GFlowNets do not enable the optimization of our objective, namely maximizing the product of all cells' read count probabilities rather than setting profile probabilities proportional to fixed, pre-specified rewards (further discussed in Supplementary Section A.3.8).”*

We also further discuss similarities with GFlowNets in the methods and go into details in Supplementary Section “Comparison of our reinforcement learning method to existing approaches”.

" perform bias correction and segmentation."

89: GC bias correction, right? What do you mean by segmentation? And how is it performed and used?

Yes, GC bias and mappability bias correction. We have now modified the text to state this. By segmentation, we mean grouping the bins into segments that are of constant copy number for all cells. We have now added an additional reference to the supplementary section in which the details of segmentation are described. In short, segmentation is performed by detecting breakpoints in the genome for which there is meaningful evidence for a change in copy number. Segmentation is used in order to split the genome into larger segments (relative to very small 100kb bins) which are more practical for downstream copy number calling. The revised manuscript now states (in Results Section “CNRein algorithm”):

*“To do so, we perform phasing and initially split the genome into K bins of length 100kb in order to perform GC bias and mappability correction and segmentation.”*

"uses low-noise regions to estimate the cell-specific scaling factor"

91 How and where is this described?

The procedure is described in supplementary materials “Estimating cell-specific scaling factor” and we have now added an additional reference to this supplementary section in Results Section “CNRein algorithm”.

In short, the variance in read-depth in regions is used to determine regions with a consistent low noise read depth that must correspond to some integer copy number. The cell-specific scaling factor is then determined by prioritizing those regions lining up with integer total copy numbers.

" based on a 96 generative model for copy number profiles that sequentially adds CNAs to clones starting from the normal 97 clone"

95: So paths are generated rather than trees? You need to explain this to the reader.

Yes, paths are generated rather than trees. We have modified the text here to say “Our algorithm generates paths to CNA profiles using an evolutionary model for copy number profiles that sequentially adds CNAs to clones starting from the normal clone.”

" In contrast to existing methods, "

95: Again, this is incorrect there are other evolutionary aware methods.

Thank you for the correction as we were not considering total copy number calling methods when writing that sentence. We have modified the text to “In contrast to existing allele-specific methods”.

" views "

97: Less anthropomorphisms please

We have now changed this to “implements”.

"r a single simulation instance with a whole genome duplication."

Fig 2. where in the tree is the WGD?

The WGD is at the trunk of the tree. We have now corrected the text to clarify this. This is also how CNAsim simulates WGD.

"A small amount of jitter was added to predictions with exactly 1,000 unique copy number profiles for clarity"

Fig 2 d: Jitter. Please explain.

Our use of jitter refers to adding a small amount of randomness (from a uniform distribution ranging from -25 to 25) to the y-axis of the plot to make data points not overlap in order to be visually distinct. This jitter is just for improving the visualization. We now state the following in the text.

*“A small amount of jitter (drawn from Unif(-25,25)) was added to predictions (y-axis) with exactly 1,000 unique copy number profiles for clarity.”*

" a truncal "

104 Define truncal and why only truncal?

By a truncal WGD we meant a WGD that occurred in an ancestor of all tumor cells (thus present in the trunk of the phylogeny). Truncal WGDs are very common in the data we are analyzing, including on breast cancer patient S0, breast cancer patient TN3, and on the WGD samples within ovarian cancer patient 2295. To follow this common (though not absolute) pattern, some of our simulations include truncal WGDs. This pattern is also used in CNAsim, generating only truncal WGD when considering WGDs.

"starting with either a normal clone or a whole genome duplicated clone,"

105: Why would a WGD happen before all other events? Why does it produce interesting data?

Although in reality other CNA events may precede a WGD event, in practice the same level of copy number variation can be simulated by multiple CNA events occurring after the whole genome duplication. Thus, our simulator is capable of generating realistic levels of copy number variation found in real data. Consequently, this assumption is used by other existing simulators such as CNAsim.

As for including WGD itself, WGD increases the difficulty of copy number calling in multiple ways. First, whole genome duplications increase the difficulty of ploidy estimation since higher integer copy numbers are percentagewise closer. Thus small shifts in the estimated ploidy may result in read depths aligning with different integer copy numbers rather than non-integer values (which is not the case for small ploidy values). Second, having a higher overall ploidy (as a result of WGD) makes the percentage variation in read depth and BAF smaller for a single deletion or amplification.

"CNAs to existing clones,"

We have now modified the text to say “generates CNAs on existing clones”.

" accuracy as the average 114 proportion of bins across all cells for which the predicted allele-specific copy number matches the true "

We modified the text to state the following.

*“First, we defined accuracy as the average proportion of bins across all cells for which the ground-truth allele-specific copy numbers were inferred (ignoring the ordering of alleles; see Section 5.3).”*

" For the shown simulation instance "

115: In fig 2a

We have now changed the text in Section “Evaluation on simulated scDNA-seq data” to state “For the simulation instances in Fig.2a”.

" Note that high parsimony values may be re- 138 flective of homoplasy, i.e., identical CNAs occurring independently at many locations on the tree, due to a 139 lack of evolutionary structure in the predictions even if the predictions do not look very visually noisy as is 140 the case for CHISEL's prediction."

137: Hard to parse.

We agree and have broken this sentence up into the following two sentences.

*“Note that high parsimony values may be reflective of homoplasy, i.e., identical CNAs occurring independently at many locations on the tree, due to a lack of evolutionary structure in the predictions. This can lead to high parsimony values even if the predictions do not look very visually noisy as is the case for CHISEL's prediction. “*

" We note that Alleloscope achieved an even lower parsimony 173 score of 3,651, however, we believe this is due to Alleloscope incorrectly predicting a lack of WGD"

173: So, final parsimony is not a great measure of success.

There is no one, single metric for evaluating performance on real data. Parsimony evaluates one aspect of a method's predictions, but it does not give the full story on its own. Parsimony values are effective for determining if a method yields overly complex predictions with spurious CNAs and no coherent evolutionary explanation. However, parsimony itself gives no information about whether the predicted profiles are missing true CNAs. To evaluate if a method is missing legitimate CNAs we used SNV-based orthogonal validation. As shown in Figure 3g, Alleloscope performs extremely poorly in terms of matching orthogonal SNV data on ovarian cancer patient OV2295. To make the mismatch between SNVs and CNAs more intuitive, Figure 3f shows that truncal SNVs that occur on copy number {2, 1} according to Alleloscope’s predictions have VAF values concentrated around 1/2 indicating they occur on half of the 3 copies which is not possible. Instead, they must occur on some integer number of copies, yielding integer multiples of 1/3 VAFs. Figure3e shows this is the case for our method’s predictions.

As an additional simple analysis (as suggested by Reviewer 1), we now measure fit to read depth in supplementary section “Comparing copy numbers with read depth”, finding Alleloscope fits the read depth on ovarian cancer patient OV2295 far worse than other methods.

In general, there is a false positive and false negative tradeoff between inferring spurious CNAs and missing legitimate CNAs. Parsimony only evaluates one side of the tradeoff (avoiding spurious CNAs) and SNV-based orthogonal validation supplements the other side of the tradeoff. We agree that parsimony alone would not be a good measure of success.

"Alleloscope did not predict WGD for any cell."

Fig 3: what does predicting WGD mean?

We appreciate you bringing our attention to the lack of clarity here. We utilized a ploidy above 3 as an indicator of WGD and have now added this to the text.

" predictions are far noisie "

Fig 3 b: How are the cells ordered? if it is based on a clustering based on the deepcopy copy number calls, then others may look more noisy. Anyway, noisiness is a so subjective term that it cannot be used unless turned into something quantifiable.

Yes. We agree that this is subjective and have removed it from the caption. We use parsimony to evaluate spurious CNAs and noise, thus there was no reason for us to subjectively make this point in Figure 3b. In the visualization, the cells are ordered based on our method's predictions, however, that does not impact objective metrics that capture noise such as parsimony.

" are likely truncal, i.e."

176: The term truncal is used earlier.

We have now removed “i.e. present in the most recent common ancestor of all tumor cells”

" likely "

We use the term likely since we do not know the ground truth and have to estimate which SNVs are truncal.

" must have a variant allele frequency (VAF) on those cells equal to some integer multiple of 1/(XA +XB 179 )"

179: This is true not only for truncal and a stronger statement can be made. What are you assuming regarding the order between the first duplication in the genomic segment of the SNV and the introduction of the SNV? In S8 The SNV is present before the duplication. In fact you make the stronger assumption below. Consequently, right here the assumption of truncal SNV is unnecessary. Moreover, the text should be better organized.

You are correct that if you measure the VAF in all cells with some SNV and with copy number (X_A, X_B), then the VAF will be an integer multiple of 1/(X_A + X_B) regardless of whether the SNV is truncal. However, in practice, one does not know ahead of time which cells have the SNV. In fact, on low-coverage data, many cells will only have a single read in the position of the SNV which may be either a variant read with the SNV or a reference read. Thus, one must instead measure the VAF on all cells (with the appropriate copy number) in the sample which is only valid for SNVs present on all cells. Therefore, we specify truncal SNVs.

We assume the SNV occurred prior to any CNAs on that segment for the stronger assumption that yields a VAF of X_A/(X_A + X_B) or X_B/(X_A + X_B). We specifically state “To better quantify differences between methods, we developed a statistical test for goodness of fit with the SNV data, exploiting the fact that the vast majority of truncal SNVs seem to precede their coinciding CNAs (Supplementary Section A.4.3).“

"each truncal SNV's "

191: How do you determine whether an SNV is truncal?

We determine an SNV to be truncal if it occurs on all sections/samples of some patient. When introducing the term truncal we state “SNVs that are likely truncal, i.e., present in the most recent common ancestor of all tumor cells (details provided in Section 4.4).”. To make sure it is very clear that the definition is in Section 4.4, we now modified the text to explicitly state that the definition in Section 4.4: “SNVs that are likely truncal, i.e., present in the most recent common ancestor of all tumor cells (as defined in Section 4.4).”

" avoids spurious CNAs producing a more parsimonious solution than SIGNALS or 201 CHISEL while retaining a high accuracy towards legitimate CNAs "

200: This core argument is not convincing.

We agree that the parsimony score alone would not be very convincing, since it only addresses spurious CNAs without addressing accuracy towards legitimate CNAs. However, we hope that our various responses demonstrate how other evaluations such as the SNV-based orthogonal validation on real data, and the accurate reconstruction of intra-tumor heterogeneity on simulations help to supplement the parsimony results. Specifically, they provide evidence that our method avoiding spurious CNAs is not a result of missing legitimate CNAs and smoothing over intra-tumor heterogeneity.

We have added the following text to the start of the results section.

“*On simulated data, we directly evaluate the accuracy of our method as well as its effective reconstruction of the ground truth level of intra-tumor heterogeneity. On real breast cancer and ovarian cancer data, it is impractical to fully encapsulate accuracy with a single test. Instead, we first evaluate avoiding spurious CNAs with parsimony based scoring. Then we evaluate sensitivity to legitimate CNAs with an SNV-based orthogonal validation (as well as an evaluation of matching read depth in supplemental section “Comparing copy numbers with read depth”). The combination of these tests reflects both precision and recall, giving a more meaningful evaluation of methods than either test in isolation.*”

" a slightly worse but not significant fit than Alleloscope (LLR −35.24 and p = 0.205). VAF 235 values are plotted in Fig. S17, showing only minor differences between methods. Additionally in Fig. S18, 236 we show that DeepCopy, SIGNALS, and CHISEL predict allelic mirroring on copy number {2, 1}, but this 237 prediction is supported by orthogonal SNV data only for DeepCopy and SIGNAL"

234: The SNV based test can only distinguish between certain pairs of copy number configurations.

Yes, there are certain copy number pairs that can not be distinguished by the SNV based test. Specifically, the SNV based test can distinguish between any pair of copy numbers that do not differ precisely by a constant multiple. More mathematically, the SNV based test can distinguish between (X_1, X_2) and (Y_1, Y_2) so long as there is no constant c such as X_2 = c X_1 and Y_2 = c Y_1.

However, all other cases can be differentiated. For instance, if one method predicts (2, 1) for all cells while another method predicts (2, 1) for some cells and (1, 2) for some cells (allelic mirroring), this can be differentiated by the SNV-based orthogonal validation. We have added the following text to the end of the evaluation subsection of the methods section.

*“It is worth noting that there are certain copy numbers that this SNV-based test can not differentiate. Namely, the copy numbers (X_1, X_2) and (Y_1, Y_2) can not be differentiated if they differ by a constant factor c such that X_2 = c X_1 and Y_2 = c Y_1.”*

"ons are far noisier "

Figure 4: Noise is not well fine

We agree and have now removed this.

" we have shown that DeepCopy avoids spurious CNAs by producing a far more parsimo- 240 nious solution with fewer unique copy number profiles than "

239: This has to be rephrased

We have rephrased and simplified the sentence. If the new sentence is still not ideal, please let us know. The sentence was previously:

*“In summary, we have shown that DeepCopy avoids spurious CNAs by producing a far more parsimonious solution with fewer unique copy number profiles than SIGNALS, CHISEL, and Alleloscope, which is in line with the clonal structure commonly observed in breast cancer that results from punctuated evolution [40].“*

The sentence is now:

*“In summary, CNRein avoids spurious CNAs, producing a far more parsimonious solution than SIGNALS, CHISEL, and Alleloscope, in line with commonly observed punctuated evolution in breast cancer [40].“*

" DeepCopy models 265 the probabilities of trajectories starting on the normal cell and ending on some copy number profile"

264: This should be explained much much earlier

We agree. We have now included the sentence

“*Specifically, it generates paths to CNA profiles using a generative model for copy number profiles that sequentially adds CNAs to clones starting from the normal clone.*”

earlier in the results section (in response to your earlier comment).

" more realistic clone sizes than existing methods"

269: This ambiguity concerning whether the methods are single cell number calling or clonal cop number, must be straightened out

Our method performs single-cell copy number calling. We have now corrected the confusing language (which seemed to indicate clonal copy number calling). We used the term “clone” simply to describe the observed pattern in our single-cell copy numbers. The sentence now states “more realistic numbers of cells with shared profiles (clones)” to make it clear that the term “clone” just describes an observed phenomenon in our single-cell copy number predictions.

"NPs"

312: use both the terms SNP and SNV. If you define them in the same way, use only one and provide the definition. Otherwise, give both definitions.

They are different which is vital to the orthogonality of our SNV-based validation and we have now clarified this in the text. By “SNP” we refer to a single nucleotide polymorphism of natural germline variation in the human genome between individuals. These are used to derive the BAF signal. The term “SNV” refers to single nucleotide variant mutations as a result of the tumor. SNV mutations are not used for constructing the BAF since they can not be phased into haplotype blocks because they are not naturally occurring in reference genomes' germline DNA. Meanwhile, SNP mutations are filtered out when creating the SNV signal since they do exist in reference genomes' germline DNA, indicating they are likely not somatic mutations caused by the tumor. Thus, these two signals are independent. The SNP signal is used for copy number calling. In contrast, the SNV signal is not used by our method or any method in our comparisons, and therefore we utilize it to form an orthogonal validation metric. We have now added the following text to the first mention of SNPs (in Results subsection “Evaluation on an ovarian cancer dataset sequenced with DLP+ technology”).

“*By SNVs we refer to somatic mutations, which, in contrast to germline SNPs, are not used by any method for inference.*”

"This then produces the B-allele frequencies (BAFs) 314 B = [B 1,...,B N ]⊤ ∈ [0, 1]N × L , which measure the proportion of reads coming from the second haplo-315 type (typically set to be the minor haplotype), i.e., B s,i ≈ P(1) s,i / (P(1) s,i + P(2) s,i )"

314: This is in conflict with the above definition that has one of P^(1)_i and P^(2)_i has the paternal, i.e., uses 1 or 2 to indicate paternal.

The definitions we gave were ambiguous and somewhat confusing giving the apparent contradictions which we have now corrected. The reviewer states “This is in conflict with the above definition that has one of P^(1)_i and P^(2)_i has the paternal, i.e., uses 1 or 2 to indicate paternal.” We have now modified this sentence referenced by the reviewer to remove the mention of maternal and paternal as below.

*“Additionally, the copy numbers are phased across each chromosome such that copy numbers P^(1)_i,P^(2)_i) = (2, 1) and (P^(1)_j,P^(2)_j) = (1, 2) for two different positions i and j on the same chromosome indicate two distinct copy numbers.”*

The other definition was indicating how one orders the two haplotypes for a chromosome based on copy numbers. We have clarified the text to say “typically set to be the haplotype with a lower average copy number in that chromosome”.

"Pr(B s | ΣB s ,P)"

326: why isn't this conditioned by R?

It is not conditioned on R because the probability of observing different BAF values given the copy number is not dependent on the total number of non-allele specific reads (i.e. reads that do not contain SNPs for phasing to alleles). To help make things more clear in the text we added the following plate diagram to Figure 6a as well as text referencing it.

"Supplementary Section A.2.1."

328 This overly distributed organization hinders the reader's understanding. Please define the distributions here.

We have now added this.

" we define Pr(P | θ) 332 as the probability of the copy number profile P according to a model with tumor-specific parameters θ "

331: Give an intuitive explanation of the parameter theta

The parameters θ are all of the weights and biases of our deep neural network. We now state the following in the text.

*“For our method CNRein, the parameters theta are the weights and biases of our deep neural network which we use in combination with an evolutionary model to calculate Pr(P | theta).”*

"P∈P"

336 (2): Why sum over P when you use argmax in (4)?

The Equation 4 (formerly Equation 2) gives the total probability given the parameters theta summing over all possible copy number profiles. Equation 6 (formerly Equation 4) gives the probability given a single copy number profile. Equation 4 is used to train the parameters of the model theta. Equation 6 is used to predict maximum likelihood copy number profiles given the trained model.

" counts for K bins of size ℓbin "

348: Before there was a discussion about segmenting now the segments seem to be fixed

There exist initial bins which are then formed into segments. In order to determine the breakpoints of segments, we calculate the read depth and BAF values on small initial bins. We modified the text to say “initial” bins to clarify the distinction from final segments.

" The genome is then split into L segments consisting of many 100kb bins, as 354 described in Supplementary Section A.1.3"

Yes, these are the final segments used, each consisting of many initial 100kb bins.

353: Each consist of many bins? Why is this done like this? Why don't you just work with the bins?

Segmentation or clustering of fixed-size bins is a typical procedure in copy-number calling used also by CHISEL and SIGNALS. The initial bins have a very weak BAF (and to some extent read depth) signal and especially do not have enough signal to estimate the noise within a bin. However, small (100kb) consecutive bins are very likely to have the same allele-specific copy number unless there is a breakpoint, or clear shift in the read depth or BAF data. CNRein (as well as other copy number callers) creates these initial, fixed-size bins specifically to process them into larger segments with better BAF and read depth signals together with noise estimates of these signals.

To illustrate the issue of the small number of reads, on breast cancer patient S0, the mean number of allele-specific reads per 100kb bin is 1.53. Below is a histogram of the number of reads per 100kb for all cells (including normal cells) for this patient.

For ovarian cancer patient OV2295 the coverage is a bit higher with the mean number of allele-specific reads per 100kb bin being 12.94. Below is a histogram of the number of reads per 100kb for all cells for this patient.

"eural network."

Fig 6 d: What is the input?

The below diagram of the network is now provided in Figure 6d.

The first input is the copy number profile. From this, the start location of the CNA is determined. Then, using both the copy number profile and the CNA start location, the end location is determined. Finally, using these variables the network outputs the copy number change via amplification or deletion. A precise description of the network architecture is given in the “Neural network architecture” section (in Methods)).

"hese"

Fig 6 d: Unclear reference.

We changed the text to say “The probability of CNA events are determined by a neural network.”

"he set ̃ P of copy number profiles returned by NaiveCopy is used to help guide 362 DeepCopy's optimization. Fig. 6a-b shows a diagram of this pipeline."

362: It doesn't provide a lot of insight.

An explanation for how the profiles of CNNaive guide CNRein are provided in Supplementary Section A.3.5. We have now modified the sentence to include a reference to this section “The set P of copy number profiles returned by CNNaive is used to help guide CNRein’s optimization as described in Supplementary Section A.3.5.”

"ach cancer is trained on independently,"

367: Rephrase.

We have improved the phrasing. It now states “The model is trained on each patient”.

"This process repeats unit terminating"

375: Spelling.

It has been corrected to say “until”.

"e define the reward function as 411 r(G) = NX s=1 Pr(R s,B s | θ)− 1 Pr(R s,B s | G)."

411: This resembles Gflow nets (GFNs). Using the developed GFN machinery, this paper could be made much more readable and elegant.

We agree that there is some conceptual similarity with GFlowNets as we have discussed in response to a previous comment. However, GFlowNets are solving a different problem statement. We have now added the following text and added a reference to the new supplementary section comparing our method with GFlowNets.

*“A theoretical alternative to avoid calculating this normalization factor via sampling would be to use GFlowNets. However, GFlowNets optimize for terminal state probabilities being proportional to rewards which would not allow for our problem statement of maximizing the overall probability of the observed dataset”*

We have now added text explaining differences with GFlowNets in supplementary section “Comparison of our reinforcement learning method to existing approaches”.

"To do so, rather 427 than looking at segments, we evaluated the K bins with a fixed size of 100kb."

427: explain

We clarified the text to say “*Since each method may yield different segmentations, we compare the predictions of each method with the ground truth on the fixed-size 100kb bins*.”.

"a,b,a′,b′)"

439 (10): Here, you don't seem to keep track of maternal and paternal.

Yes. We do not simulate the complexities of linkage disequilibrium and haplotype blocks and instead generate already-phased inputs. Thus, we do not distinguish maternal and paternal alleles for this evaluation metric. We now state the following in the text (in Section “Methods” subsection “Evaluation”).

*“Since we do not simulate the complexities of linkage disequilibrium and haplotype blocks and instead generate already-phased inputs, we define accuracy using unordered allele-specific copy numbers rather than distinguishing between maternal and paternal haplotypes.”*

"Finally, parsimony values are calculated with edge 439 lengths determined by the ZCNT distance."

439: But the standard parsimony score does not depend on edge lengths.

We now utilize Lazac for calculating parsimony and have therefore removed this text as it no longer applies.

"amplification on one allele"

693: understandable but probably not correct terminology

We have modified the term “amplification” to “gain”.

"The probability of observing values of CA s,η,CB s,η assuming there is no imbalance comes 700 from a binomial distribution which can be approximated by a Gaussian. Specifically, CA s,η − CB s,η has a mean 701 of zero, and a variance equal to the total number of counts CA s,η + CB s,η"

700: These probabilities have not been introduced yet, so a better organization is desirable. Furthermore, when introduced, the definitions are slightly different. Finally, I didn't get this variance, please explain.

We have now added substantial additional clarification to the text. In Supplementary Section “Determining haplotype-specific read counts”, we first state the following, explaining the binomial distribution without any Gaussian approximation.

“*If there is no imbalance, then read counts on either allele are equally likely and so the counts C^A_{s,\eta} and C^B_{s,\eta} come from a binomial distribution with p = 1/2.Thus, assuming no imbalance, the probability of observing the values C^A_{s,\eta} and C^B_{s,\eta} is*”.

We then provide the below equation.

We then state

“*Given this distribution, the imbalance quantity C^A_{s,\eta} - C^B_{s,\eta} has a mean of zero and a variance equal to the total number of counts (C^A_{s,\eta} + C^B_{s,\eta}).*”

This also clarifies that the variance comes from the variance of a binomial distribution with p = 1/2 and a total of C^A_{s,\eta} + C^B_{s,\eta} counts. Then, after explaining this we state the Gaussian approximation prior to giving the Gaussian equation.

“*Additionally, this binomial distribution can be closely approximated with a Gaussian distribution where the imbalance quantity C^A_{s,\eta} - C^B_{s,\eta} has a mean of zero and a variance equal to the total number of counts (C^A_{s,\eta} + C^B_{s,\eta}).*”

"For any two haplotype blocks η and η′ in the same chromosome, we want to phase the "

710 This depends on the above assumption.

Yes, it assumes that an imbalance in the allele counts reflects evidence for an imbalance in the copy number.

"Additional phasing algorithm details:"

728: This section must be better explained.

We have now modified the text to improve the explanation. This includes adding a figure with an example, and repeatedly describing aspects of the algorithm more intuitively in terms of the example.

"2ΣR s,i"

878: The denominator must be explained. The total copy number is often used here.

The denominator is twice that variance following the equation for a Gaussian. We have now clarified this in the text by adding “the following product of Gaussians with variances Sigma^R_{s, i}.”

"Pr(R s,B s | θ)− 1"

1003 (57): GFNs would most likely assist with estimating the normalizing factor.

Estimating the normalization factor is not difficult for our specific objective since it simply requires estimating probabilities for each cell in a finite list of cells. However, if instead we needed to normalize by calculating the probability of every copy number profile encountered during training then a tool like GFlowNets would be required. We agree that GFlowNets provide a great general tool for estimating such normalizing probabilities in a more diverse set of problems for which calculating the normalization factor is more difficult. In fact, although GFlowNets do not directly apply to our problem definition, it’s possible that some aspects of GFlowNets could be useful for future work extensions of our algorithm. We appreciate you bringing this to our attention.

"as defined in Supplementary Section A.3.8."

1008: This layer-after-layer organization makes reading and understanding hard.

We have modified the text to give the below intuitive definition. The mathematical technicalities in Supplemental Section A.3.8 are not helpful for understanding the section but are references for the sake of completeness.

*“Intuitively, generative sequences in G \in \mathcal{A}(P) generate the profile P (g(G) = P) without needless additional steps such as adding CNA events only to remove those exact same CNA events (mathematical technicalities of this definition are provided in Supplementary Section A.3.8)”*

"Efficient generative sequences"

1068: The content of A.38 can be expressed more efficiently and readable.

We believe this section was difficult to read primarily due to us stating definitions without giving proper motivation and context for the reader to intuitively understand the definitions. We have now modified the text to correct this.

"such that the distance from P to P′ is 1135 equal to the distance from P′ to P, i.e., ZCNT(P,P′) = ZCNT(P′,P)"

1135: inefficient description.

This has now all been removed since we now use Lazac as suggested by the reviewer.

"ZCNT(P(1) ,Q(1) ) + ZCNT(P(2) ,Q(2) )."

1137 (69): This requires keeping track of maternal and paternal. Do all methods do that?

This has now all been removed since we now use Lazac as suggested by the reviewer. Yes, only haplotype-specific callers have been used in this analysis (SIGNALS, CHISEL and Alleloscope).

"Once all internal node copy number profiles are determined, 1147 we can calculate the parsimony score of the tree by again applying ZCNT distances. Specifically, the length 1148 of each edge is equal to the ZCNT distance, as defined in equation (69), between the copy number profiles 1149 of the nodes connected by the edge. Then, the parsimony score is the sum of distances on all edges."

1147: This is an entirely reasonable approach. Still, it is also not standard, and it is hard to judge how well it fits the CN profile reconstruction methods.

We agree that this is a reasonable consideration and now use Lazac as suggested by the reviewer.

"As discussed in the main text, and shown in Supplementary Section B.1, the 1182 majority of truncal SNVs are of the first type in our data"

1182: But will the fraction of the second type influence your results?

The small number of SNVs of the second type would add some SNVs with a VAF equal to some integer multiple of 1/(X_1 + X_2), which would slightly worsen the log likelihoods for each method. The VAF plots indicate that the small number of SNVs do not substantially bias the VAF distribution away from the expected distribution and so the small number of such SNVs do not greatly bias the results of our statistical analysis.

"max(Pr( v, r | X (1) , X (2) , A-allele), Pr(v, r | X (1) , X (2) , B-allele))."

1194: This means that you don't distinguish between maternal and paternal. When and how must be better organized.

We do not know whether the SNV occurs on the maternal or paternal allele and thus we take the maximum of the two probabilities. However, we do distinguish between maternal and paternal in the sense that the SNV must be either consistently on the maternal or consistently on the paternal allele across all cells. Pr( v, r | X (1) , X (2) , A-allele) gives the probability assuming the SNV is consistently on the A-allele across cells, and Pr( v, r | X (1) , X (2) , B-allele) gives the probability assuming the SNV is consistently on the B-allele across cells. We clarify this as follows (in Supplementary Section “Calculating the probability of SNV counts given copy numbers”).

“*Thus, while we do not know if the SNV occurs on the maternal or paternal haplotype, we do enforce that the SNV must be either consistently on the maternal or consistently on the paternal haplotyple across all cells.*”

"VAFs of truncal SNVs overlapping with various copy numbers inferred by DeepCopy on ovarian cancer patient OV2295 [20]. VAFs are shown for all the top-11 copy numbers with the most SNVs (or equivalently, all copy numbers with at least 140 S"

Fig S10: SO Chisel and Signals do really well earlier. It would be interesting to see how well they do wrt this test.

We have now included CHISEL and SIGNALS. The purpose of this analysis was just to demonstrate the VAFs or rare copy numbers rather than to specifically comparatively test the quality of different methods. With that being said, there is no reason to not include the results of SIGNALS and CHISEL and so we have now added these results in the supplemental materials. Specifically, below are the relevant plots as well as the original plot for our method. These plots are not sufficiently sensitive to subtle differences in copy number profiles to demonstrate major differences between methods. For this reason, we provided the SNV based statistical analysis which can determine more subtle mismatches between SNVs and CNAs.

SIGNALS:

CHISEL:

Our method:

"and the color of the nodes matches the UMAP clusters"

Fig S20: This means that those clusters are far from monophyletic; that is, either the trees or the clusters are far from correct.

DBSCAN clustering of CNA profiles was used in the Minussi et al. paper. We used a similar procedure here. Since the clustering does not use an evolutionary tree, it might not correspond to clades of the tree. Nevertheless, the clusters do allow us to get a rough idea of the clonal structure of the tumor, and evaluate consistency between the two sequencing technologies.

**Reviewer 3:**

Reviewer #3: The authors proposed DeepCopy for haplotype-specific copy number calling on scDNA-seq data. DeepCopy applies a deep reinforcement learning system to correct and estimate the final copy number profile from 'raw' copy number profiles initially estimated from low-noise regions by NaiveCopy. By comparing DeepCopy with CHISEL, Alleloscope and SIGNALS on simulated and real data, they claim that their evolution-aware model produces more realistic clones with identical CN profiles, leading to more parsimonious CNA phylogenies. I think the use of reinforcement learning for CNA calling is an excellent idea. However, there are still some issues that need to be addressed before it can be accepted for publication.

We thank the reviewer for their largely positive assessment and have now improved the paper by addressing the issues you point out.

Major:

1.	The method descriptions are too technical in Methods and Supplementary sections, while too simple in the Results section. It would benefit a broader audience if a more intuitive introduction to the DeepCopy model could be provided in the main text and Method sections. In particular, a brief description of the intuition behind the reinforcement learning framework and a more detailed explanation of how it is adapted to the CNA calling task would benefit readers unfamiliar with reinforcement learning.

We completely agree and have both modified and added to the discussion of our reinforcement learning system in the results section. Specifically, we now state the following.

*“In contrast to existing allele-specific methods, this joint estimation is evolution-aware.*

*Specifically, our algorithm generates paths to CNA profiles using an evolutionary model for copy number profiles that sequentially adds CNAs to clones starting from the normal clone.*

*The probability of each CNA event in this trajectory is determined by deep convolutional neural network. The reinforcement learning system implements the addition of mutations to clones as actions and utilizes a reward that maximizes the probability of the observed read counts given the model. Intuitively, high rewards are given to copy number profiles that fit cells read depth and BAF data, especially when existing high probability copy number profiles do not yet fit those cells well. After training, inference is performed by selecting copy number profiles with the highest overall probability for each cell given both the observed read counts and evolutionary model probabilities.”*

In the methods section, we concluded that the previous description of the reward function was too technical with no intuitive explanation given. Consequently, we have also added the below text to the methods section.

*“Intuitively, this function rewards trajectories that result in profiles that closely fit cells' read count data as indicated by Pr(R_s, B_s | G). Additionally, more reward is given if those cells do not yet have high probability profiles that fit them well as indicated by Pr(R_s, B_s | theta)^-1. Without the term Pr(R_s, B_s | theta)^-1, reinforcement learning would result in 100% probability given to the single best copy number profile rather than finding a set of copy number profiles that fit all cells.”*

In the problem statement section, we decided to add a sentence intuitively explaining the problem we wish to solve before getting into the technical definition of the problem. After the first sentence stating we wish to identify copy number profiles for each cell, we add the below intuitive description.

*“These copy number profiles should fit each cells' read count data well in addition to forming a coherent solution across all cells.”*

Towards the beginning of the reinforcement learning section, we originally stated that we used sampling and immediately transitioned to a technical description of this sampling. Instead, we now have the following transition sentence to improve clarity.

*“Intuitively, we generate copy number profiles and highly reward profiles that nicely fit the observed data, yielding parameter updates that increase the probability of the observed data given the model.”*

2.	Can you clarify the generating process. If I understand correctly, the process generates a new CNA tuple given the current state which is represented by CNA profiles Ρ. The process stops at a generated STOP state. However, it is not clear to me how the elements in CNA tuple are generated. In Line. 390-393 and figure 6d, they assign a probability to each element, how these element wise probabilities were joined to represent the probability of the new tuple? In addition, the probability for each element seems to be generated by a softmax function which gives a distribution of all possible output state, do you choose the best state for each element and combine them to form the final output tuple to be generated?

“If I understand correctly, the process generates a new CNA tuple given the current state which is represented by CNA profiles Ρ. The process stops at a generated STOP state.” this understanding is correct. In terms of the elements of the CNA tuple, their probabilities are generated autoregressively. Specifically, first, the start position is generated. Then, once a specific start position is selected, these are fed into the neural network for calculating the end position probabilities. Then, once the end position is selected, the start position and end position are fed into the neural network for calculating the probability of different changes in copy number (amplification and deletion). This is now shown more clearly in Figure 6d.

Using an autoregressive system rather than independently generating the start, end, and copy number change is essential. As a simple example, imagine the correct tree consists of only one clone with only an amplification of chromosome 1 haplotype 1 and one clone with only a deletion of chromosome 2 haplotype 1. Imagine each clone consists of the cells. Our model would first generate the position either being chromosome 1 or chromosome 2 each with 50% probability. Then, if the position was chromosome 1 it would generate an amplification with 100% probability and if the position was chromosome 2 it would generate a deletion with 100% probability. This gives a 50% probability of each clone. However, imagine if instead we independently generated the position and copy number change (not autoregressively). Then, it would assign 50% probability to chromosome 1 and chromosome 2 as well as 50% probability to amplification and deletion. Thus, we would be fitting a model where one fourth of the cells have deletion of chromosome 1, and one fourth of the cells have amplification of chromosome 2, despite there being no such cells in reality.

In terms of choosing the best copy number profile (for the final inference of copy number profiles for each cell), we do not need to take the best state for each element. Doing so would greedily estimate the highest probability profile according to our model, but would not consider which profile best fits the particular cell. Instead, we first take the plausible set of profiles across all cells generated during training and evaluate their probability Pr(P | theta) as in equation 6. Then, we evaluate the probability Pr(R_s, B_s | Sigma^R_s, Sigma^B_s, P) of each cell’s observed read depth and BAF measurements given each of these plausible copy number profiles as in equation 1. We then maximize the product of these two quantities in equation 4 to determine which profile has the overall highest probability for each cell.

We greatly appreciate you taking the time to understand the relatively complex inner workings of our method!

3.	Does the above generating model allow CNAs to be generated on overlapping regions on the same haplotype? Do you constrain the number of events to be generated?

Yes, it allows CNA to be generated on overlapping regions on the same haplotype as can occur in real cancer evolution. We now explicitly state that our model supports overlapping CNAs in Methods as in the below text.

*“Then, a CNA occurs producing a new clone. The new clone has a copy number profile in which the values in either P^(1) or P^(2) are modified to new values for some interval of segments within a chromosome. This interval is allowed to overlap with other existing CNAs.”*

We do not explicitly constrain the number of events to be generated. However, one technical aspect of our algorithm is “efficient generating sequences” as described in Supplementary Section A.3.8, which (together with a limited number of segments and a limited total copy number) limits the number of events that can be generated. Intuitively, “efficient generating sequences” prevent the algorithm from generating some copy number profile in a completely inefficient manner such as creating CNA events and then precisely undoing those same CNA events that were just created. This limits the number of events necessary to read any copy number profile. However, “efficient generating sequences” do not prevent the model from, for example, amplifying all of haplotype 1 on chromosome 1 and then deleting that extra copy of haplotype 1 in some segment within chromosome 1.

4.	How is the CNN structure related to the generating model? A graphical representation of the structure may be beneficial.

We originally had a small overview of the model in Figure 6d, but have now substantially expanded it to better demonstrate the CNN structure. Additionally Figure 6c now shows how the model is used to generate a copy number profile. The convolutional layer processes the copy number profile into a vector used for calculating CNA probabilities. Other inputs (such as the start position for determining the end position, etc) are encoded as a vector, transformed with a fully connected layer, and then added to the profile vector. Output probabilities are determined from this vector using a fully connected layer and the softmax operator. The new Figure 6d is shown below.

5.	In the simulation set-up how do you assign fitness values?

The fitness value of a clone is either equal to the fitness of its parent clone or doubles the fitness of its parent clone. The probability of the fitness doubling is a parameter of the simulation instance and is set to 1/6, 1/8, 1/10, 1/12, 1/14, 1/16, 1/20, 1/50, 1/100, or 1/1000. For high probabilities of fitness increases, there are frequent clonal expansions and clonal sweeps, resulting in many shared mutations across clones and less intra-tumor heterogeneity. For low probabilities of fitness increase, there is a large amount of intra-tumor heterogeneity. The spectrum of intra-tumor heterogeneity is reflected in the number of unique copy number profiles, shown in Figure 2d. We select a constant increase in the fitness (doubling) for simplicity since it allows a single parameter (probability of fitness increase) to control intra-tumor heterogeneity. These and other additional details of the simulation setup are in supplementary section “Simulation set-up”.

6.	Is DeepCopy sensitive to the estimates of R, B and \Sigmas? Will unreliable estimates of R, B and \Sigmas, distort the results of DeepCopy?

Unreliable estimates of the read depth and BAF (R and B) will distort the results of DeepCopy as well as all other methods. The quality of a method’s outputs is always limited by the quality of input signals. For unreliable estimates of \Sigmas, the answer is more subtle. Dramatic underestimations of some Sigma values may cause our method to fit noisy read depth of BAF values in some segments and predict spurious CNAs. Alternatively, dramatic overestimations of Sigmas could result in our method missing some legitimate CNA. Moderately inaccurate estimates would only create this issue in borderline cases, for instance, CNAs with barely enough evidence to be detected (since most CNAs have much more than the minimum statistical evidence required for detection). To demonstrate this empirically, we ran our algorithm with all variance value in Sigma_R and Sigma_B artificially multiplied by 10. This moderate change in sigma values significantly alters the tradeoff between avoiding spurious CNAs and detecting legitimate CNAs. Specifically, it puts a much greater emphasis on avoiding spurious CNAs at the sacrifice of detecting legitimate CNAs. On ovarian cancer patient OV2295, we ran the SNV-based test yielding the following plots showing a slight reduction in accuracy for the case with sigmas multiplied by 10.

On the left are the results with sigmas multiplied by 10, and on the right are the original results. The original log likelihood ratios with SIGNALS, CHISEL, and Alleloscope are -1033.4, 3418.6, and 12635.3 respectively. Using incorrect sigmas resulted in slightly worse log likelihood ratios of -1346.1, 3106.0, and 12403.7 for SIGNALS, CHISEL, and Alleloscope respectively. We also measured parsimony values, and found that multiplying sigma values by 10 decreases the parsimony value as shown below.

The parsimony values of SIGNALS, CHISEL, and Alleloscope are 34,529, 13,769, and 4,149 respectively. The original parsimony value of our method is 7,163. With sigma values multiplied by 10, spurious CNAs as well as legitimate uncommon CNAs are removed, resulting in a parsimony value of 2,100.

On breast cancer patient S0, the SNV-based test similarly shows a slight reduction in accuracy in the case where sigma values are multiplied by 10. The difference is sufficiently small to be relatively difficult to see visually in the below plots.

However, with the incorrect sigma values the log likelihood ratios between our method and SIGNALS, CHISEL, and Alleloscope are 79.9, 1018.9, and -61.4 respectively. In contrast, the original log likelihood ratios (with the correct sigma values) for SIGNALS, CHISEL, and Alleloscope are 145.3, 1093.8, and -35.2 respectively. Thus, using incorrect sigma values causes our model to fit the orthogonal validation slightly worse. We also analyzed the effect on parsimony, finding that the parsimony values decreased as expected with artificially high sigma values.

Note that the parsimony value for our method with sigma values multiplied by 10 is so much lower than that of SIGNALS that it is very difficult to see in the bar plot. The parsimony values for SIGNALS, CHISEL, and Alleloscope respectively are 40,222, 8,506, and 2,076 respectively. The original parsimony value of our method is 397. The parsimony value of our method with artificially increased sigma values is 209.

All of these results indicate that inaccuracies in sigma values result in a modified tradeoff between avoiding spurious CNAs and detecting legitimate CNAs. However, our method is not overly sensitive to moderate inaccuracies in calculated sigma values.

7.	DeepCopy samples from the initial estimates from NaiveCopy. How different is the final profiles P compared to P-tilde?

The median L1 distance between the predictions of CNRein (formerly DeepCopy) and CNNaive (formerly NaiveCopy) on ovarian cancer patient OV2295 is 0.0868. For comparison, the median L1 distance between CNRein and SIGNALS is 0.08769. Note, that an L1 distance of 1 would indicate an average difference in the copy numbers of 1 across the genome. Differences between all these methods are typically relatively subtle. This information is now included in Supplementary Section “Benchmarking modifications of CNRein”.

Minor:

8.	The prior based on the generating process in DeepCopy seems to improve the estimation of CNAs at the trunk or in large subclones. Would it miss or be less sensitive to low frequency CNA?

We have now added an additional analysis in Supplementary Section “Accuracy in detecting small clones”, to directly address this question (as well as a similar concern of Reviewer 1).

Our existing simulations show that CNRein accurately reconstructs copy number profiles on simulated data with varying level of intra-tumor heterogeneity, including accurately reconstructing the number of unique copy number profiles (Fig.2d). However, the existing results did not specifically address whether CNRein accurately reconstructs the low frequency clones (resulting from low frequency CNAs). We now analyze the clonal structure on simulated data to ensure CNRein reconstructs small clones accurately (as opposed to only accurately reconstructing truncal CNAs or large subclones). Specifically, we computed cells belonging to small clones of 1 to size 10 in the ground truth. We then observed the predicted clones according to CNRein, SIGNALS and CHISEL to compare the true clone size for these cells with the predicted clone size. More specifically, as stated additionally in our response to Reviewer 1, we do the following.

For each ground truth clone size N, we calculate the set of cells S_N which belong to clones of size N in the ground truth. We then observe the predicted clones according to our method, SIGNALS, and CHISEL. The predicted clone size for a cell s is equal to the size of the clone that cell s belongs to on the predicted copy number profiles. Using this, we calculate the median predicted clone size for each set S_N. If a method perfectly reconstructs the clonal structure then S_N would be equal to N for all N. If a method randomly reconstructs clones unrelated to the ground truth clones, then S_N would be uncorrelated with N. The below plot this median value for N ranging from 1 to 10 for each method.

As shown in the plot, our method very accurately reconstructs these small clone sizes, including cell-specific events (N=1). In contrast, for SIGNALS and CHISEL the median predicted clone size is 1 (indicating a cell-specific event) independent of the ground truth clone size. This very strongly demonstrates the accuracy of our method at detecting small clone sizes including cell-specific events on simulated data. To show more information beyond the median, we also generated the below boxplots of predicted clone sizes for our method, SIGNALS, and CHISEL.

To conclude, CNRein accurately reconstructs intra-tumor heterogeneity, including low frequency CNAs rather than only accurately reconstructing truncal events or events on large clones.

On real data, CNRein continues to predict low frequency CNAs, however, in the absence of ground truth data one can specifically determine if these predicted low frequency CNAs are correct. Specifically, on ovarian cancer patient OV2295 CNRein predicts a large amount of intra-tumor heterogeneity with 408 out of 617 cells having cell-specific copy number profiles. On breast cancer patient S0, CNRein predicts much less intra-tumor heterogeneity with only 113 out of 783 cells to have cell-specific copy number profiles.

9.	Does other methods also make consistent estimates across sequencing technologies in section 2.5?

These datasets for breast cancer patient TN3 are so low coverage that other methods struggle to produce meaningful results. For instance, the ACT sequencing technology dataset has a coverage in the range from 0.015x to 0.005x, in contrast to around 0.1x for breast cancer patient S0 and around 0.2x for ovarian cancer patient OV2295.

SIGNALS contains noise based filters on cells and bins. After filtering overly high noise bins, SIGNALS filtered out cells that have too few bins with sufficient signal. Specifically, on the ACT sequencing data, SIGNALS stated in the terminal “Total number of cells after removing cells with < 100 bins: 3”. This number should be closer to 1,000 in order to produce a meaningful output. After attempting to perform clustering, SIGNALS crashed without producing copy number outputs.

For CHISEL, we were able to produce an output file but predicted copy number profiles seem unreliable. Below is a heatmap of CHISEL’s predictions.

The large amount purple indicates copy numbers exceeding 6. The results using ACT sequencing are substantially noisier than the results using 10x sequencing due to the low coverage of the ACT sequencing data (0.005x to 0.015x) relative to the 10x sequence data (coverage of 0.01x to 0.07x). As a minor technical note, in order to successfully run CHISEL, we provided CHISEL with the intermediate data format of read counts rather than starting with BAM files (to avoid some errors that occurred when running CHISEL from BAM files). This technical note is not relevant for the other real data results since we used publicly available CHISEL predictions for those datasets. Isolating a low noise cluster of cells for the higher coverage 10x technology does show some agreement between CHISEL’s predictions and the largest cluster in our predictions as in the below comparison.

Applying UMAP to the full set of CHISEL predictions on both sequencing technologies gives the below plot.

There are no meaningful shared clusters to perform downstream analysis on.

10.	Fig 3b and 4b, the region with small CNAs can be circled or highlighted in the figure for better understanding.

Thank you for the helpful suggestion. We have now added this (using a box with a red outline).

11.	In the method description DeepCopy and NaiveCopy apply an initial bin size of 100kb. Is it necessary to adjust this bin size when applied to ultra shallow technologies? Say ACT.

Extremely noisy input data is acceptable for our segmentation step, which then groups bins into larger segments. Even if some 100kb bins have zero allele-specific reads, this is not problematic for our segmentation algorithm. Therefore, even on ultra-shallow (coverage 0.005x to 0.015x) ACT sequencing, it was acceptable to use an initial bin size of 100kb. Very low coverage data will likely result in high noise estimates for each segment. This will help the algorithm avoid inferring spurious CNAs from high noise fluctuations in read counts.

12.	The author used many mathematical notations and symbols in Methods and Supplementary sections. It is recommended to include a glossary table with the list of notations.

Thank you for the helpful suggestion, and we have now included this (Table S1 “glossary of notation”).

**Second round of review**

**Reviewer 1**

The authors have satisfied my concerns. Great work!

**Reviewer 2**

The submission is too long with respect to content, and in particular with respect to results. It is now a 30-page paper with a 65-page supplement (and a rebuttal consisting of around 60 pages).
It is hard not to get the impression that the authors aim for quantity, not quality; the high-quality parts are insufficient. Most differences in comparisons to other methods are, in one way or another, interpreted to the advantage of DNRein. It falls short of providing reasonable arguments for biological data. Instead, we get a sequence of opinions that “seems to be spurious” without proper argumentation. In answer 7 to reviewer 1, there is now a multi-page argument resting on the statement: “In contrast, if cell-specific CNA events for copy number C’ are generally legitimate, one does not expect a lopsided distribution.” However, many distributions have a skew, i.e., are lopsided. So, the underlying assumption is simply wrong. In answer 8 to reviewer 1, there is no significant difference to Signals,i.e., the read-depth analysis in the rebuttal doesn’t show any advantage compared to Signals. Also, the previous results showed a similar trend.

Moreover, I do not see the value of comparisons with clone methods, given that the authors now write that their method reconstructs single-cell profiles. Comparing apples and pears is not useful. Measuring how well a clone method reconstructs a single-cell tree is not useful. In addition, in a real data set, each cell will have its own copy number profile, so reconstructing a smaller number of profiles is not a feature. Moreover, with fewer profiles, the parsimony score will be lower, and with cruder profiles, the score will also be lower, so parsimony can not be considered a sufficient evaluation criterion (not even when combined with SNVs). In summary, the advantages of the proposed method can only be shown on synthetic data, which is not sufficient, particularly given the length, the remaining poor readability, and the ad hoc nature of many of the new components.

**Reviewer 3**

The manuscript has been significantly improved in this revised version. The authors have addressed my previous concerns.

**Authors Response**

**Point-by-point responses to the reviewers’ comments:**

Our central premise is that one should avoid inferring excessive variation in copy number across cells when not sufficiently supported by the data. In that light, CNRein’s findings of fitting the data at least as well as (and often better than) existing methods while inferring more shared clonal structure is a preferable outcome. In other words, CNRein is based on the generally agreed upon principle in computational biology that in the absence of contrary evidence one should prefer a more parsimonious solution. Upon reading Reviewer 2’s comments, we realized that this point was not made sufficiently clear in the Results section. Therefore, we made minor textual updates to the sections “Evaluation on an ovarian cancer dataset sequenced with DLP+ technology” and “Evaluation on a breast cancer dataset sequenced with 10x Chromium CNV technology”. In addition, we included two previous supplemental figures (showing fit to read depth) as panels in main text Figures 3 and 4.

Reviewer 2:

The submission is too long with respect to content, and in particular with respect to results. It is now a 30-page paper with a 65-page supplement (and a rebuttal consisting of around 60 pages).

We do not agree that the length of our paper is excessive. Single-cell DNA copy number calling from BAM files is a complex problem, especially when calling allele-specific copy numbers rather than total copy numbers. Consequently, the paper for the method CHISEL is 27 pages with a 57 page supplement, the paper for the method Alleloscope is 31 pages with a 39 page supplement, and the paper for the method SIGNALS is 30 pages with a 31 page supplement. Given the double spacing of our text, we believe the length of our paper is in line with existing work on this problem.

It is hard not to get the impression that the authors aim for quantity, not quality; the high-quality parts are insufficient. Most differences in comparisons to other methods are, in one way or another, interpreted to the advantage of DNRein. It falls short of providing reasonable arguments for biological data. Instead, we get a sequence of opinions that “seems to be spurious” without proper argumentation. In answer 7 to reviewer 1, there is now a multi-page argument resting on the statement: “In contrast, if cell-specific CNA events for copy number C’ are generally legitimate, one does not expect a lopsided distribution.” However, many distributions have a skew, i.e., are lopsided. So, the underlying assumption is simply wrong.

We disagree with the statement that we have not provided strong biological arguments for the advantages of CNRein. We believe there is a general misunderstanding behind the key premise of our method. Briefly, our paper argues that one should not construct unique copy number profiles for cells when there is not sufficient evidence to support these unique copy number profiles. Indeed, our simulations confirm that only CNRein produces accurate levels of copy number variation (as reflected by the number of unique copy number profiles matching the ground truth) in contrast to all existing allele-specific methods, which infer spurious CNAs. Our real data analyses demonstrate that existing methods have a tendency to infer an excessive variation in copy numbers across cells, often producing unique copy number profiles for each cell, without sufficient data support. On the other hand, CNRein provides simpler explanations of CNA evolution, with more clonal structure (as quantified by parsimony score) and shared copy number profiles, without sacrificing data fit (as reflected by fit to read depth and VAFs of truncal SNVs).

Reviewer 2 is referring to a secondary analysis requested by Reviewer 1 who wanted us to verify that CNRein does not smooth over legitimate CNAs inferred by other methods. To that end, the reviewer requested us to focus on cell specific CNAs, i.e., CNAs inferred to be unique to a single cell, and assess whether these are supported by read depth data. The quote “seems to be spurious” comes from a description of an illustrative example shown to Reviewer 1 who asked to see “the raw read depth alongside the inferred copy number state”. After showing this, we define more objective metrics based on the skew of scaled read depth distributions. Reviewer 2’s statement that “many distributions have a skew” does not imply that a skew indicates nothing in this particular case. In our case, perfectly inferring cell specific CNAs would result in a distribution centered on the predicted copy number. In the absence of ground truth, we believe this secondary analysis suggested by Reviewer 1 to be valuable, indicating that CNRein does not smooth over legitimate CNAs relative to existing methods.

In answer 8 to reviewer 1, there is no significant difference to Signals,i.e., the read-depth analysis in the rebuttal doesn’t show any advantage compared to Signals. Also, the previous results showed a similar trend.

CNRein is based on the premise that one should only infer variation in copy number across cells when sufficiently justified by evidence in the data. Thus, CNRein matching SIGNALS’ fit to read depth and truncal SNV support while producing a vastly more parsimonious solution (with more shared clonal structure) is a positive result. One should disfavor a more complex solution when a simpler explanation is equally supported by the data.

To better illustrate this point, we made minor textual updates to the sections “Evaluation on an ovarian cancer dataset sequenced with DLP+ technology” and “Evaluation on a breast cancer dataset sequenced with 10x Chromium CNV technology”. In addition, we included two previous supplemental figures (showing fit to read depth) as panels in main text Figures 3 and 4.

Moreover, I do not see the value of comparisons with clone methods, given that the authors now write that their method reconstructs single-cell profiles. Comparing apples and pears is not

useful. Measuring how well a clone method reconstructs a single-cell tree is not useful.

We would like to point out that the methods SIGNALS, CHISEL, and Alleloscope, which we compare against, infer single-cell CNA profiles. CHISEL includes an additional post-processing step to produce clonal copy number profiles whereas the method VICTree by design outputs only clonal copy number profiles. We included these additional two methods at the explicit request of Reviewer 2.

A key advantage of our method is the prediction of more parsimonious clonal structures so it is useful to also compare against clonal methods that explicitly enforce clonal structure. We do not evaluate where individual cells are placed on the tree but rather evaluate overall parsimony, which results from the predicted clonal structure of the set of unique copy number profiles. We find that the clonal method VICTree produces excessive parsimony and inaccurate profiles (compared to the simulated ground truth) in contrast to the insufficient parsimony of single-cell methods as well as clonal CHISEL. On the other hand, CNRein produces an accurate clonal structure.

We believe that these additional comparisons are valuable for the sake of completeness, and decided to keep them in the manuscript.

In addition, in a real data set, each cell will have its own copy number profile, so reconstructing a smaller number of profiles is not a feature. Moreover, with fewer profiles, the parsimony score will be lower, and with cruder profiles, the score will also be lower, so parsimony can not be considered a sufficient evaluation criterion (not even when combined with SNVs). In summary, the advantages of the proposed method can only be shown on synthetic data, which is not sufficient, particularly given the length, the remaining poor readability, and the ad hoc nature of many of the new components.

The reviewer states “In addition, in a real data set, each cell will have its own copy number profile, so reconstructing a smaller number of profiles is not a feature.” Again, a central premise of our paper is that one should not construct unique copy number profiles for cells when there is not sufficient evidence to support these unique copy number profiles. In other words, we believe that CNRein’s ability to infer fewer profiles without sacrificing fit to read depth and orthogonal SNVs is a key feature. This cannot be trivially achieved by crudely reducing the number of unique copy number profiles as Reviewer 2 indicates. In fact, in answer 10 to Reviewer 1, we show that crudely reducing the number of unique copy number profiles substantially worsens the fit to SNVs. On real data, we find that CNRein’s solutions provide simpler explanations of CNA evolution without sacrificing data fit. Moreover, our simulations (based on real data of patient S0, as well as using the simulator CNAsim suggested by this reviewer) demonstrate CNRein’s improved accuracy relative to existing methods. Therefore, we believe that CNRein is a valuable contribution to the field.

**Third round of review**

**Reviewer 1**

I believe that the latest round of complaints raised by reviewer 2 are unfounded.

1. I am satisfied that the authors adequately addressed our comments with additional analyses, method details, and discussion. There need be no additional consternation regarding the responses to my comments.

2. The length of the paper is, as the authors point out, consistent with the lengths of similar single-cell copy-number calling methods as the problem is complex. Frankly, we should expect a longer paper from a later method (such as this) that is tasked with presenting a novel contribution and juxtaposing its merits with a more crowded field.

3. The reviewer appears to have a fundamental disagreement with the main argument of the paper: that single cells should not be assigned unique copy-number profiles from each other unless there is evidence to separate them, and that assigning unique profiles in absence of such evidence could lead to spurious conclusions. While this may or may not be an accurate description of the true biology (perhaps all cells would be unique if we could observe them perfectly), it is an exceedingly reasonable principle to use when solving a problem that involves very sparse data, like copy-number calling from low-pass scWGS. In this regard it is very similar to maximum parsimony principles that are widely adopted in evolutionary biology and tumor genomics.

**Reviewer 3**

I have no further questions.
